# Power load forecasting combining deep learning models and improved CLPO algorithm

**DOI:** 10.1371/journal.pone.0351428

**Published:** 2026-06-29

**Authors:** Jianguang Song, Xiang Wei, Zhipeng Li, Xia Zhang, Guoheng Zhang, Lu Wang

**Affiliations:** 1 Electric Power Dispatch Center, State Grid Gansu Electric Power Company, Lanzhou, China; 2 Digital Business Division, State Grid Gansu Electric Power Company, Lanzhou, China; 3 Digital Communication Department, State Grid Wuwei Power Supply Company, Wuwei, China; 4 Power Dispatch Center, State Grid Wuwei Power Supply Company, Wuwei, China; 5 Smart Energy Business Division, Gansu Tongxing Intelligent Technology Development Co., Ltd., Lanzhou, China; Khalifa University, UNITED ARAB EMIRATES

## Abstract

The accuracy of power load forecasting directly affects the stable operation of the power system and energy optimization configuration. In response to the complex nonlinear characteristics of load data and the tendency of existing methods to fall into local optima, this study selects time-series convolutional networks, Long Short-Term Memory (LSTM) networks, and bidirectional LSTM to propose a combined deep learning model through weighted reconstruction. On this basis, the parrot optimization algorithm is introduced for iteration and strategy optimization, and joint error correction is performed to propose a new power load forecasting model. The experimental results show that under the same conditions and with the same hyperparameters, the conventional gradient descent optimization method takes about 330 rounds to reach 0.85 and the highest is only about 0.89. While the proposed method reaches 0.85 in about 200 rounds and eventually approaches 0.95. The overall convergence is faster and the accuracy is higher. On the UK-NGED dataset, the long-term prediction mean absolute errors of Transformer-DGNN, EEMD-Attention and VMD-ResNet are 0.214, 0.238 and 0.257 respectively, and the research method is 0.181. Simultaneously, continuous learning-based parrot optimization and error correction model suppress weight drift and short-term bias during optimization, maintaining prediction stability under concept drift and non-steady load patterns. When prediction performance degrades, error-monitoring-based lightweight retraining rapidly restores model accuracy. Under extreme weather conditions, the root mean square error of the baseline method is 0.295–0.318, and that of the research method is 0.250. To confirm that the differences are not due to random fluctuations, a two-sample t-test is conducted on the root mean square error distributions from 10 independent experiments. After explicitly calculating the mean difference, variance, and degrees of freedom, the result yields *p* < 0.05, indicating that the reduction in model error under extreme meteorological conditions is statistically significant. Therefore, the model can maintain high accuracy prediction in variable load environments and has stronger adaptability in high penetration rates of new energy and extreme weather conditions. This study provides more accurate and reliable decision support for smart grid scheduling and optimization.

## 1. Introduction

The reform of the electricity market has led to a trend of diversification and complexity in electricity demand, making accurate load forecasting particularly important. The research on Power Load Forecasting (PLF) involves multiple disciplinary fields, including power engineering, data science, artificial intelligence, statistics, and economics. Its core goal is to establish mathematical models or smart algorithms by analyzing historical data and environmental variables to forecast the future electricity load demand for a certain period [1]. In traditional power systems, PLF is mainly used for power generation planning and grid scheduling to guarantee a balance between power supply and demand. The modern power system is gradually developing towards the direction of the smart grid. The large-scale integration of new energy, the promotion of market-oriented electricity trading, and the rise of electric vehicles and distributed energy have further highlighted the role of PLF [2]. Accurate load forecasting can enhance the efficiency of power grid operation and reduce power generation costs, as well as optimize the allocation of power resources and reduce carbon emissions, which is of great significance for building a low-carbon and sustainable energy system. Many researchers have conducted varying degrees of exploration on PLF. Abumohsen et al. established a model built on the measurement results of the current power load of a certain power company, combined with Long Short-Term Memory (LSTM) and Gated Loop Unit, to achieve power load estimation. The model performed the best in accuracy and error, with a minimum Mean Absolute Error (MAE) of 0.0326 [3]. Liang H et al. developed a two-stage short-term PLF method built on Temporal Convolutional Network (TCN) to address feature selection after pattern decomposition and enhance the accuracy of PLF models. This method has demonstrated superior prediction accuracy and processing efficiency in public datasets [4]. Deng Q et al. proposed an echo state network for Short-Term Load (STL) forecasting in power systems. With the support of this network, the accuracy of STL power forecasting has been significantly improved, and it has also demonstrated strong robustness in different forecasting environments [5]. Qian F et al. found that during the PLF process, if all source domain data are indiscriminately extended to the target domain, it is highly likely to lead to negative transfer learning. To this end, researchers have proposed an enhanced prediction transfer method by combining the Extreme Boosting algorithm and the R2 algorithm. Compared with a single source domain, this method significantly improved prediction accuracy and reduced the maximum error by 7.23% [6]. Zhang X et al. proposed a novel prediction method to further enhance the effectiveness of existing PLF methods by optimizing through error correction after mixing Convolutional Neural Networks (CNN) and LSTM. Compared with existing long-term load forecasting methods, this method had higher prediction precision [7].

Due to the fact that the core of PLF lies in accurately modeling the time series characteristics of load data, current research generally faces four major challenges. These challenges include the nonlinearity and randomness of load data, high-frequency variations in STL forecasting, coupling effects of multiple variables, and insufficient model generalization ability [8]. In response to these shortcomings, researchers have successively carried out Deep Learning (DL) optimization. For example, Karim F K et al. introduced a whale optimization algorithm for parameter optimization in Recurrent Neural Network (RNN) and proposed an enhanced PLF model. This model was more effective than other models in predicting the trend of electricity data [9]. Hossain M A et al. designed a novel framework for forecasting power load by integrating empirical mode decomposition and the emperor butterfly optimization algorithm. This method had a lower computation time and improved MAE by 32.96% [10]. Tsai W C et al. designed a model grounded on RNN and ant colony optimization algorithm for long-term and medium-term PLF. By updating the weights of each sub-model, this framework effectively improved the comprehensive predictive ability of the model and enhanced its stability [11]. Ahmed QI et al. proposed an intelligent detection model by combining SVM and GWO algorithm to more accurately identify abnormal load behavior of solar photovoltaic modules in power transmission. This model divided the inverter state into two categories: normal and faulty, and the GWO algorithm could greatly improve the detection ability of abnormal data [12]. Miraftabzadeh et al. believed that photovoltaic power transmission load forecasting had a high degree of random behavior, while existing methods suffered from multivariate coupling effects. To this end, researchers have developed a novel PLF method built on LSTM, incorporating transfer learning for training and multi-objective optimization algorithms for parameter optimization. This method had an MSE of 0.55 and a weighted MAPE of 47.07%, resulting in higher accuracy [13]. Existing STLs exhibit elasticity and instability characteristics that challenge accurate load forecasting. To this end, Dai L et al. proposed a short-term PLF model that combines the whale optimization algorithm, convolutional block attention, and bi-directional gated loop cells. Experimental results showed that the model exhibited excellent uniform dispersion of the initial population and an effective ability to identify optimal solutions compared to other models [14]. Chen J et al. came out with a learning model that is a combination of integrated intelligent optimization algorithms based on LSTM, variational modal decomposition, and multi-strategy optimization of dung-beetle algorithms. Experimental results showed that compared with the traditional intelligent algorithm, this new model not only solved the shortcomings of the traditional optimization dung beetle algorithm but also improved the stability, global optimization ability, and information utilization of the model [15]. Pang X et al. proposed a short-term PLF method based on a Bagging stochastic configuration network. First, the missing values in the original data were filled with the mean values. Second, the influencing factors, such as weather and weekly type data, were coded. Then, the STLs were predicted using the Bagging integration algorithm in conjunction with the coded influencing factor data. The results showed that the proposed method for short-term and medium-term load forecasting had high prediction accuracy and was significantly effective in improving load forecasting accuracy [16].

In summary, although some public load datasets exhibit relatively stable overall trends, PLF still requires strong robustness under complex conditions such as emergencies, renewable energy fluctuations, and high load shocks. Traditional statistical models, such as Autoregressive Integrated Moving Average (ARIMA) and exponential smoothing, perform well on linear or low-dimensional series, but they struggle to capture nonlinear dependencies, mixed long–short-term patterns, and high-frequency variations. To address this issue, the study combines TCN, LSTM, and Bidirectional LSTM (BiLSTM) through Weighted Reconstruction (WR) to build a Combinatorial Deep Learning (CDL) model, and further integrates Continuous Learning-based Parrot Optimization (CLPO) and Error Correction Model (ECM) to construct an end-to-end forecasting framework.

Compared with existing studies, the innovations of this paper are primarily reflected in the following three aspects:

(1) Theoretical Innovation: This study establishes a collaborative optimization modeling framework based on a unified objective function, integrating multi-scale feature representation, model fusion weights, parameter optimization processes, and error correction mechanisms into a unified optimization system. This creates a closed-loop interaction mechanism of “features-weighting-error”. Unlike traditional staged modeling or post-processing correction methods, the framework theoretically achieves coupled optimization across multiple modules, transforming independent model optimization into an integrated collaborative convergence process. This approach significantly enhances the stability and consistency of modeling complex nonlinear load sequences.
(2) Technological Innovation: A parameter optimization strategy combining Weighted Reconstruction (WR) and Continuous Learning Parrot Optimization (CLPO) is proposed to achieve adaptive fusion of multi-model outputs and dynamic global parameter search. The WR mechanism enhances multi-scale feature integration through dynamic weight updates, while CLPO improves global search capability and convergence efficiency by incorporating continuous learning and adaptive parameter tuning strategies, effectively avoiding the tendency of traditional optimization algorithms to get trapped in local optima.
(3) Application Innovation: Designed for complex power load forecasting scenarios, we developed an integrated CDL-CLPO-ECM prediction system that deeply integrates forecasting models with error correction mechanisms, enabling an end-to-end closed-loop optimization process from data input to result refinement. This approach maintains stable predictive performance even under high volatility and extreme load conditions, providing an engineering-ready solution for power load forecasting in multi-source disturbance environments.

## 2. Methods and materials

To address the issue of unbalanced integration of different time-scale characteristics in power load forecasting and the tendency of optimization strategies to fall into local optima, the overall method process can be summarized as “data input - multi-scale feature extraction - adaptive fusion - parameter optimization - error correction - prediction output”. Firstly, historical power load data and auxiliary variables such as meteorology and time are preprocessed and used as model inputs; secondly, in a combined deep learning framework, the time series convolution network is used to extract short-term fluctuation features, the long short-term memory network is used to model long-term dependencies, and the bidirectional long short-term memory network is used to enhance the bidirectional temporal feature expression of the sequence; subsequently, through the weighted aggregation method, the outputs of each sub-model are adaptively fused to achieve dynamic weight allocation of information from different time scales; on this basis, the continuous learning parrot optimization algorithm is introduced to jointly optimize the model weights and key parameters to enhance the global search ability and convergence stability; finally, through the long-term balanced error correction mechanism, the prediction residuals are dynamically compensated to output the final load forecasting result.

### 2.1. Construction of cdl models

#### 2.1.1. Overall prediction process and problem modeling.

**Fig 1:**
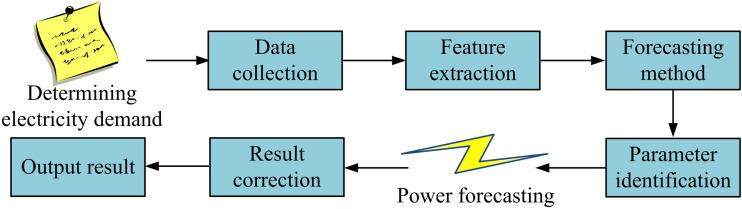
Power load forecasting process.

With the advancement of electricity marketization reform, the improvement of load forecasting accuracy is of great significance for reducing power generation costs, optimizing energy dispatch, and supporting the consumption of new energy. Especially with the increasing proportion of new energy, the volatility of power load has increased, making it difficult for traditional forecasting methods to meet actual demand [17]. PLF typically follows a systematic process, as shown in Fig 1.

Fig 1 shows the entire process, which includes requirements analysis, data collection and processing, feature extraction, method selection, parameter optimization, model prediction, and result correction. Among them, feature extraction and method selection are key steps that affect prediction accuracy. Traditional time series analysis methods, including autoregressive moving average models and exponential smoothing, perform well on linear trend data, but are difficult to effectively handle complex nonlinear load changes [18–19].

#### 2.1.2. LSTM and bilstm time series modeling mechanisms.

**Fig 2:**
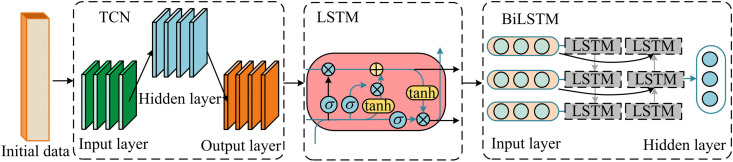
Structural framework of CDL.

In addition, in practical applications, a single prediction method often has limitations, such as traditional statistical methods being difficult to capture nonlinear changes in load. Although DL models have strong fitting ability, there is still room for optimization in extracting long-term features [20–21]. Therefore, this study attempts to construct CDL and comprehensively utilize the advantages of multiple DL methods to lift the accuracy and robustness of PLF. The framework of CDL is shown in Fig 2.

In Fig 2, CDL adopts the TCN-LSTM-BiLSTM structure, which combines TCN, LSTM, and BiLSTM to fully extract the temporal features of power load data, improving prediction accuracy and generalization ability. Firstly, TCN serves as a feature extraction layer, utilizing causal convolution to ensure that predictions do not rely on future data. That is, for input sequence $X=\left\{x_{\text{1}},x_{\text{2}},\text{\textbackslash{}ldots},x_{T}\right\}$, the output $v_{t}$ at time t only depends on the current and past time steps, as calculated in Equation (1).


$$\begin{array}{c} v_{t}=\sum_{i=0}^{k=0}W_{u}\cdot x_{u-i} \end{array}$$ (1)

In Equation (1), $v_{t}$ represents the convolution output at time t; $x_{u-i}$ represents the value of the input sequence at time u−i. $W_{u}$ and k are the convolution kernel’s weight and size. By expanding convolution, the Receptive Field (RF) exponentially grows with the number of layers, resulting in the RF of the l -th layer as shown in Equation (2).


$$\begin{array}{c} R_{l}=(k-1)\sum_{j=0}^{l-1}d_{j}+1 \end{array}$$ (2)

In equation (2), $R_{l}$ represents the receptive field size of the l layer; l represents the number of network layers. $d_{j}$ means the expansion rate of layer j. In addition, TCN uses residual connections to alleviate the gradient vanishing issue, and the output at layer l is shown in Equation (3).


$$\begin{array}{c} H_{l}\;=f(W_{l}\;*H_{l-1\;}+b_{l}\;)+H_{l-1}\; \end{array}$$ (3)

In Equation (3), $H_{l}\;$ represents the output feature of the l layer; $H_{l-1\;}$ represents the input feature of the l−1 layer; $W_{l}$ represents the convolution kernel weights of the l layer; * represents the convolution operation; $b_{l}$ represents the bias term; f(_) represents the nonlinear activation function. Through the operations of Equations (1) to (3), the features generated by TCN will be used for LSTM to further learn the dynamic changes in power load. In LSTM memory units, state updates rely on gating mechanisms, where the Forget Gate (FG), Input Gate (IG), and Output Gate (OG) respectively control the forgetting, storage, and output of information, as defined in Equation (4).


$$\begin{array}{c} \left\{ \begin{array}{c} @c@f_{t}=\sigma (W_{f}\;h_{t-1}\;+U_{f}\;x_{t}\;+b_{f}\;)\\ i_{t}\;=\sigma (W_{i}\;h_{t-1\;}+U_{i}x_{t}\;+b_{i}\;)\\ o_{t}\;=\sigma (W_{o}\;h_{t-1}\;+U_{o}\;x_{t}\;+b_{o}\;)\\ c_{t}\;=f_{t}\;⊗c_{t-1}\;+i_{t}\;⊗tanh(W_{c}\;h_{t-1\;}+U_{c}\;x_{t}\;+b_{c}\;)\\ h_{t}\;=o_{t}\;⊗tanh\left(c_{t}\;\right) \end{array}\right. \end{array}$$ (4)

In Equation (4), σ is the Sigmoid function. ⊗ is the Hadamard product. $c_{t}$ is the cellular state. ($f_{t}$,$i_{t}\;$,$o_{t}$) and ($b_{f}$,$b_{i}$,$b_{o}\;$) are the activation values and bias terms of the FG, IG, and OG. $h_{t}$ corresponds to the Hidden State (HS) value at t. ($W_{f}$,$W_{i}$,$W_{o}$) and ($U_{f}$,$U_{i}$,$U_{o}$) are the weight matrices of the FG, IG, and OG in the HS and current state. $W_{c}$, $U_{c}$, and $b_{c}$ are parameters corresponding to IGs used to update cell states. $o_{t}$ is the activation value of the OG. LSTM is mainly used to enhance the temporal dependence of power loads, while BiLSTM further strengthens the bidirectional modeling ability of sequences. BiLSTM includes forward and backward LSTM. For the input sequence X, the formulas for its forward HS ${\vec{h}}_{t}$ and backward HS ${\overset{\leftarrow }{h}}_{t}$ are shown in Equation (5).


$$\begin{array}{c} \left\{ \begin{array}{c} @c@{\vec{h}}_{t}=LSTM\left(X_{t},\vec{h_{t-1}}\right)\\ {\overset{\leftarrow }{h}}_{t}=LSTM\left(X_{t},\overset{\leftarrow }{h_{t-1}}\right) \end{array}\right. \end{array}$$ (5)

The final HS splicing is shown in Equation (6).


$$\begin{array}{c} \overset{*}{\underset{t}{h}}={\vec{h}}_{t}⊕{\overset{\leftarrow }{h}}_{t} \end{array}$$ (6)

Finally, the features extracted by BiLSTM are mapped through a fully connected layer to obtain the final load prediction. Compared with traditional RNN-based models, CDL integrates the parallel capability of TCN, the long-term dependency modeling of LSTM, and the bidirectional learning of BiLSTM, improving prediction accuracy and robustness.

#### 2.1.3. Multi-model weighted reconstruction method.

However, multi-scale feature fusion remains suboptimal, as TCN focuses on short-term patterns while LSTM-based modules emphasize long-term dependencies, leading to imbalanced information contributions. To address this issue, WR is introduced to adaptively fuse multi-scale features through learnable weights, enabling dynamic adjustment of each component’s contribution. The formulation is given in Equation (7).


$$\begin{array}{c} \hat{y}\left(x\right)=\sum_{i=1}^{N}\omega_{i}f_{i}\left(x\right) \end{array}$$ (7)

In Equation (7), $\hat{y}\left(x\right)$ means the final predicted value. N denotes the amount of time series prediction algorithms used. $f_{i}\left(x\right)$ is the predicted result of the i -th model. $\omega_{i}$ is the weight of the predictive model. To address potential conflicting predictions among TCN, LSTM, and BiLSTM during load fluctuations—such as one model emphasizing short-term volatility while another prioritizes long-term trends, leading to inconsistent prediction directions or magnitudes—WR dynamically optimizes the weight vector using a regularized least-squares method. During optimization, if outputs from different submodels diverge significantly, the algorithm automatically reduces the weight of the more volatile submodel. This enhances the overall smoothness and stability of the prediction results.

To ensure principled optimization, WR follows two guidelines: multi-scale complementarity and risk minimization. The weight vector is constrained within a probability simplex to guarantee normalized contributions, while the objective function balances prediction error and stability penalty to achieve a Pareto-optimal trade-off. The prediction risk is measured by weighted mean squared error within each time window, and the stability term constrains weight fluctuations via L2 regularization or adjacent weight differences, thereby reducing variance and oscillation. The optimal weights are obtained by minimizing the objective function in Equation (8).


$$\begin{array}{c} \Gamma \left(w\right)=\sum_{t=1}^{T}{\left(y_{t}-\sum_{i=1}^{N}\omega_{i}f_{i}\left(x_{t}\right)\right)}^{\text{2}}+\lambda \sum_{i=1}^{N}\overset{\text{2}}{\underset{i}{\omega }} \end{array}$$ (8)

In Equation (8), Γ(w) is the weighted error loss function. $y_{t}$ is the actual power load value. T is the time step. From this, it can be known that the entire formula consists of the first term, the prediction error, the second term, the regularization penalty term, and the third term, the constraint $\omega_{i}$. These three elements jointly ensure that the multi-objective optimization problem can be transformed into a single-objective convex problem and naturally converge to the Pareto optimal equilibrium solution. λ is a regularization term used to prevent overfitting. The regularization parameter not only prevents overfitting but also directly determines the system’s ability to maintain prediction stability under varying load dynamics. Its mathematical relationship can be expressed as follows: when λ is large, the regularization term $\lambda \sum_{i=1}^{N}\overset{\text{2}}{\underset{i}{\omega }}$ suppresses extreme deviations in the weight distribution, promoting more balanced contributions from multiple submodels.

#### 2.1.4. Analysis of multi-scale feature fusion mechanism.

Consequently, during periods of intense load fluctuations, the upper bound of prediction variance is suppressed, resulting in more stable overall outputs. When λ is small, the model relies more heavily on a single submodel. While this may improve local accuracy, stability decreases. Therefore, the magnitude of λ positively correlates with the system’s predictive stability under complex load conditions. Selecting an appropriate value for λ achieves a balance between prediction accuracy and stability.

**Fig 3:**
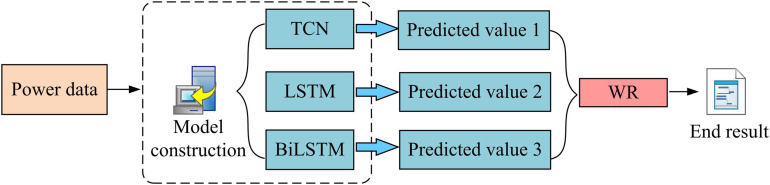
Multi-scale feature fusion and weight adaptation mechanism in the CDL framework based on WR.

In terms of convergence, Equation (8) is optimized via mini-batch training, where CLPO performs global search and local refinement of the weight vector under fixed TCN/LSTM/BiLSTM parameters. Convergence is reached when the validation loss no longer decreases or the maximum iteration is met. In online prediction, Equation (8) is applied within a sliding window, where new samples update errors via sliding or exponential decay, enabling lightweight updates without retraining. Moreover, WR dynamically adjusts submodel contributions based on residuals and real-time conditions, assigning higher weights to TCN for short-term fluctuations, LSTM for long-term trends, and BiLSTM for bidirectional corrections. The resulting CDL-WR framework is illustrated in Fig 3.

As shown in Fig 3, the entire model consists of an input data layer, a DL prediction model layer, a weight adjustment layer, and a final prediction output layer. The workflow begins with data input, incorporating various influencing factors such as historical electricity load data, weather characteristics, and temporal features, followed by the DL modeling phase. Specifically, the Time-Correlation Network (TCN) handles local pattern learning at short time scales, the LSTM model captures long-term dependency relationships, while the BiLSTM leverages bidirectional propagation advantages to enhance periodic feature extraction. After modeling completion, the three models generate prediction outputs that feed into the WR layer. This architecture not only demonstrates the feature extraction pathways of TCN, LSTM, and BiLSTM across different time scales but also achieves dynamic multi-scale information fusion through the WR layer. Unlike simple weighting methods, WR dynamically adjusts output weights of sub-models by integrating historical residuals with current input features during computation, maintaining dynamic equilibrium between short-term fluctuations and long-term trends across various load scenarios. For instance, during load spikes or peak periods, TCN pathway weights are significantly increased to strengthen local response capabilities, while in cyclical or stable phases, LSTM and BiLSTM weights dominate, thereby improving overall prediction stability and accuracy.

### 2.2. Optimization of composite plf model by integrating improved CLPO algorithm

#### 2.2.1. Basic principles of po algorithm.

**Fig 4:**
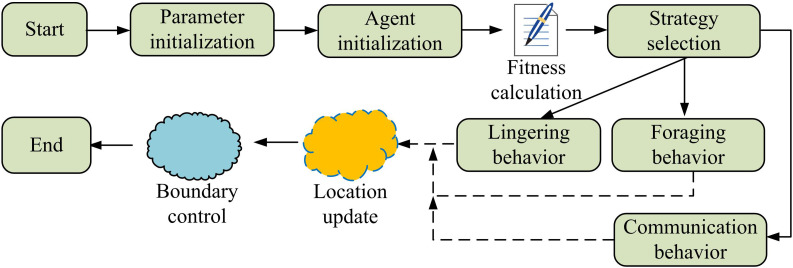
Multi-strategy search mechanism of the PO algorithm including foraging, residence, and communication behaviors.

After constructing a new type of CDL model, this study finds that although WR can optimize the fusion effect of different time scale features to some extent, improve the stability of load forecasting, there are still some problems. For example, the optimal value of the weight matrix depends on the distribution of the training data, making it difficult to maintain generalization across different datasets. In addition, the optimization process of the weight matrix usually relies on gradient descent methods, but when faced with complex nonlinear time series data, gradient descent methods may fall into local optima, leading to a decrease in the model’s performance [22–23]. Therefore, to further optimize the adjustment strategy of the weight matrix and improve the global optimization ability, this study introduces the Parrot Optimizer (PO) algorithm for model optimization. Fig 4 shows the flowchart of PO [24–26].

As shown in Fig 4, PO initializes search agents and evaluates fitness, followed by ranking and strategy selection. Each agent randomly adopts one of three update behaviors. Specifically, foraging enables global exploration via high-variance perturbations, residence performs local refinement through contraction toward the current optimum, and communication enhances global perception by sharing differential information among individuals, forming implicit gradient directions in the weight space [27]. These mechanisms jointly balance global exploration, local exploitation, and directional search within a unified iteration, enabling stable and diverse optimization in high-dimensional non-convex spaces. Compared with PSO, GA, and SA, CLPO maintains solution diversity and convergence without gradient dependence, reducing sensitivity to initialization and improving the ability to escape local optima with smoother convergence [28–29]. The foraging behavior explores high-fitness regions and updates positions based on fitness gradients, as described in Equation (9).


$$\begin{array}{c} \overset{\left(t+1\right)}{\underset{i}{X}}=\overset{\left(t\right)}{\underset{i}{X}}+\alpha \cdot \frac{\nabla F\left(\overset{\left(t\right)}{\underset{i}{X}}\right)}{\|\nabla F\left(\overset{\left(t\right)}{\underset{i}{X}}\right)\|}+\delta \cdot r \end{array}$$ (9)

In Equation (9), $\overset{\left(t\right)}{\underset{i}{X}}$ denotes the current location of the i -th search agent in the t -th generation. α is the step size factor. $\nabla F\left(\overset{\left(t\right)}{\underset{i}{X}}\right)$ is the fitness gradient of the current position. δ and r are both perturbation terms. The stay behavior maintains the current solution to preserve local optima and triggers fine-tuning when fitness fluctuations are small, as calculated by Equation (10).


$$\begin{array}{c} \overset{\left(t+1\right)}{\underset{i}{X}}=\overset{\left(t\right)}{\underset{i}{X}}+\gamma \cdot \left(\overset{\left(t\right)}{\underset{best}{X}}-\overset{\left(t\right)}{\underset{i}{X}}\right)\cdot tanh\left(\beta \cdot F\left(\overset{\left(t\right)}{\underset{i}{X}}\right)\right) \end{array}$$ (10)

In Equation (10), $\overset{\left(t\right)}{\underset{best}{X}}$ is the location of the best individual in the current population. γ is the convergence factor. $tanh\left(\beta \cdot F\left(\overset{\left(t\right)}{\underset{i}{X}}\right)\right)$ is the adjustment range. Interaction behavior adjusts positions based on neighborhood information, enabling information sharing among individuals and enhancing global search capabilities, as calculated by Equation (11).


$$\begin{array}{c} \overset{\left(t+1\right)}{\underset{i}{X}}=\overset{\left(t\right)}{\underset{i}{X}}+\sum_{j\in N\left(i\right)}w_{ij}\cdot \left(\overset{\left(t\right)}{\underset{j}{X}}-\overset{\left(t\right)}{\underset{i}{X}}\right) \end{array}$$ (11)

In Equation (11), N(i) is the neighborhood of the i -th individual. $w_{ij}$ is the coefficient of influence between individuals. After completing the position update, the algorithm determines whether the maximum iteration count has been reached. If the condition is met, the optimal solution is output; otherwise, the process enters the boundary control phase to ensure the agent remains within the feasible solution space and continues iteration.

#### 2.2.2. CLPO improvement strategy.

**Fig 5:**
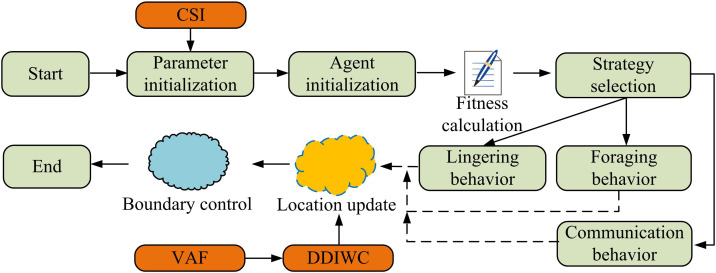
Improved CLPO optimization process with chaotic initialization and adaptive parameter update strategy.

To enhance the GSA and local search capabilities of PO, the study makes multiple improvements to PO and proposes CLPO. The process is shown in Fig 5.

As shown in Fig 5, CLPO enhances traditional PO algorithms by incorporating chaotic initialization and dynamic weight adjustment mechanisms, significantly improving search efficiency and stability. The chaotic mapping ensures more uniform initial population distribution, strengthening global search capabilities. Meanwhile, the dynamically decreasing inertial weights and velocity adjustment factors progressively reduce search step sizes during iterations, enabling a smooth transition from exploration to convergence phases. This approach facilitates the acquisition of more stable optimal solutions in complex high-dimensional parameter spaces. The optimization of CLPO can be summarized in four steps: ① Initialization via chaotic search generates a uniform initial population in the high-dimensional hyperparameter space encompassing prediction weights, learning rate, error correction coefficient, etc., with fitness calculated using objective function A (Equation 15); ② Dynamic classification of individuals into explorers and exploiters based on fitness, where explorers perform large-step jumps while exploiters fine-tune within the optimal neighborhood; ③ Continuous learning records historical optima, balancing global search and local convergence through decreasing inertia weights and velocity adjustment factors; ④ Iteration terminates when the upper limit is reached or the objective function’s decline over B consecutive generations falls below threshold C, outputting optimal parameters.

It should be noted that the “foraging–resting–communicating” behaviors in CLPO are heuristic abstractions of search operators, aiming to balance global exploration and local exploitation in non-convex, multi-modal spaces without relying on gradients. For the highly nonlinear objective composed of WR weights, network hyperparameters, and ECM terms, CLPO mainly enhances the ability to escape local optima and reduces sensitivity to initialization. When integrated with the combinatorial DL framework, CLPO encodes all key parameters into a unified high-dimensional search vector for joint optimization. By incorporating chaotic perturbation and dual convergence criteria, it effectively avoids local minima and ensures stable convergence. The CSI formulation is given in equation (12) [30–32].


$$\begin{array}{c} \overset{\left(0\right)}{\underset{t}{X}}=X_{min}+\left(X_{\text{max}}\;-X_{\text{min}}\;\right)\cdot \left|2\overset{\left(0\right)}{\underset{i}{Z}}-1\right| \end{array}$$ (12)

In Equation (12), $\overset{\left(0\right)}{\underset{t}{X}}$ means the initial location of the i -th search agent. $X_{\text{min}}$ and $X_{\text{max}}\;$ are the upper and lower bounds of the search space. $\overset{\left(0\right)}{\underset{i}{Z}}$ is a chaotic variable generated by logistic mapping. In the position update stage, the PO algorithm adopts a fixed step size strategy for search. CLPO introduces the DDIW control strategy, gradually reducing the inertia weight during the search process to balance global and local search capabilities, as shown in Equation (13).


$$\begin{array}{c} \omega_{t}=\omega_{max}-\left(\frac{\omega_{max}-\omega_{min}}{T}\right)\cdot t \end{array}$$ (13)

In Equation (13), $\omega_{t}$ is the inertia weight of generation t. $\omega_{max}$ and $\omega_{min}$ refer to the maximum and minimum inertia weights. In addition, to enhance the convergence speed of CLPO and avoid oscillation problems during the search process, CLPO further introduces SAF during position updates, dynamically adjusting the movement speed of the search agent to make it more adaptable. The mathematical expression is shown in Equation (14) [33,34].


$$\begin{array}{c} \overset{\left(t+1\right)}{\underset{i}{V}}=\chi \left[\omega_{t}\overset{\left(t\right)}{\underset{i}{V}}+c_{\text{1}}r_{\text{1}}(P_{i}-\overset{\left(t\right)}{\underset{i}{X}})+c_{\text{2}}r_{\text{2}}(G-\overset{\left(t\right)}{\underset{i}{X}})\right] \end{array}$$ (14)

In Equation (14), $\overset{\left(t\right)}{\underset{i}{V}}$ means the speed of the i -th individual in the t -th generation. $P_{i}$ is the individual’s historical optimal position. G denotes the global optimal position. $c_{\text{1}}$ and $c_{\text{2}}$ are learning factors. $r_{\text{1}}$ and $r_{\text{2}}$ are random numbers. χ is the velocity decay factor. This method enables the search agent to have a higher movement speed in the early stages to enhance GSA, and gradually converges to a local optimal solution in the later stages to improve the stability of optimization. To further enhance standardization, the optimization process of CLPO is formalized into the following objective function as shown in equation (15).


$$\begin{array}{c} J\left(\omega ,\theta \right)=\sum_{t}{\left(y_{t}-{\hat{y}}_{t}{\left(\omega ,\theta \right)}^{\text{2}}+\lambda \|\omega \|\right)}^{\text{2}} \end{array}$$ (15)

In equation (15), $y_{t}$ represents the actual load, while ${\hat{y}}_{t}\left(\omega ,\theta \right)$ denotes the prediction result generated by the CDL framework under parameter θ and weight ω. The core optimization task involves dynamically updating (ω,θ) in high-dimensional space through CLPO to minimize the objective function J. Regarding convergence criteria, CLPO employs a dual-criterion approach: first, reaching the maximum iteration count; second, early termination when the fitness decline falls below a threshold for consecutive generations, ensuring computational efficiency.

#### 2.2.3. ECM error correction mechanism.

**Fig 6:**
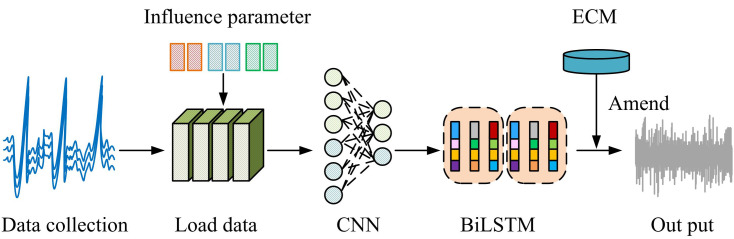
Error correction mechanism based on ECM integrated with BiLSTM prediction outputs.

In addition, this study introduces ECM to optimize BiLSTM in the third layer CDL framework, as shown in Fig 6.

As shown in Fig 6, the ECM processing workflow sequentially comprises data input, feature extraction, sequence modeling, and error correction. The input layer first receives load data and relevant influencing parameters. Convolutional operations are then applied to extract local temporal features, which are subsequently fed into the BiLSTM network. Through bidirectional propagation, the BiLSTM model learns long-term dependencies, enabling superior performance in capturing short-term fluctuations and cyclical trends. Finally, the ECM dynamically corrects short-term prediction errors based on long-term equilibrium relationships, ensuring output results remain stable and accurate under complex load conditions. In this architecture, the ECM does not operate independently. Its key correction parameters—such as the error correction coefficient and long-term equilibrium term weights—are incorporated alongside the CDL–WR module’s weights into the CLPO’s high-dimensional search vector. The CLPO and main network undergo joint optimization under the same loss function. In other words, during each iteration, CLPO dynamically updates ECM parameters using residual information generated by ECM, enabling adaptive adjustment of correction intensity in response to load fluctuations. This establishes a closed-loop compensation mechanism: “prediction → residual → parameter update.” The specific correction calculation method is shown in equation (16).


$$\begin{array}{c} \Delta y_{t}=\phi \left(y_{t-1}-\varphi X_{t-1}\right)+\sum_{i=1}^{p}\kappa_{i}\Delta X_{t-i}+\varepsilon_{t} \end{array}$$ (16)

In equation (16), $\Delta y_{t}$ represents the error correction value of t at the current moment, and the correction process is $\overset{final}{\underset{t}{\hat{y}}}=\overset{WR}{\underset{t}{\hat{y}}}\Delta_{t}$, where $\overset{final}{\underset{t}{\hat{y}}}$ represents the output of TCN-LSTM-BiLSTM, and $\Delta_{t}$ is generated from the residuals at the previous moment, the long-term equilibrium term, and the predicted changes. ϕ is the correction factor. φ is the coefficient of long-term equilibrium relationship. $\kappa_{i}$ is a short-term correction term. $\varepsilon_{t}$ is a random perturbation term. It is important to emphasize that the introduction of the ECM does not operate in isolation from the DL framework, but rather forms a joint modeling approach with the BiLSTM output layer. Let the short-term prediction error be defined as shown in equation (17).


$$\begin{array}{c} e\left(t\right)\;=\;y\left(t\right)\;-\;\overset{\mathrm{\backslash lower}0.5em\mathrm{\backslash smash}⌢\$}{\}}yNN\left(t\right) \end{array}$$ (17)

In equation (17), y(t) represents the actual load; y(t) represents the output of the deep network at time t. Based on this, ECM introduces the long-term equilibrium relationship as shown in equation (18).


$$\begin{array}{c} \Delta y\left(t\right)\;=\;a\cdot e\left(t-1\right)\;+\;\beta \cdot Z\left(t\right)\;+\;\varepsilon \left(t\right) \end{array}$$ (18)

In equation (18), Δy(t) denotes the incrementally forecasted value corrected by the ECM, a represents the coefficient of the error lag term, Z(t) signifies the long-term equilibrium constraint variable (such as trend terms or seasonal components), and Z(t) is the disturbance term. Through this formulation, the ECM integrates Z(t) prior errors with long-term constraints to influence the current forecast, ensuring the network maintains dynamic equilibrium even under non-stationary load conditions. Furthermore, addressing the non-stationary load patterns common in real-world power systems, ECM does not rely on strict stationarity assumptions. Instead, it dynamically updates parameters a and β to adaptively adjust correction intensity across different load phases, thereby enhancing the model’s adaptability to abnormal scenarios. For instance, in extreme scenarios such as high penetration of renewable energy or sudden power outages, the model automatically increases the weight of long-term equilibrium terms to suppress abnormal fluctuations. During holiday periods with sudden load surges, it reduces long-term constraints to enhance short-term responsiveness, thereby ensuring the robustness and flexibility of the forecast.

#### 2.2.4. CDL-CLPO-ECM collaborative optimization framework.

**Fig 7:**
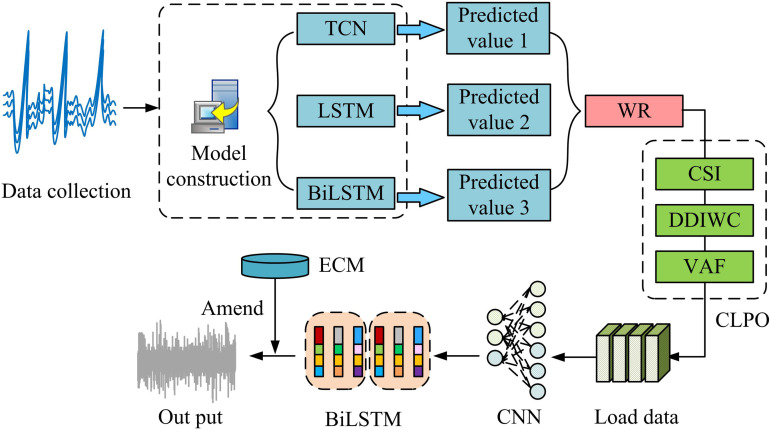
Integrated CDL-CLPO-ECM framework with joint optimization and closed-loop correction.

Finally, the paper proposes a novel PLF model that fuses CDL, CLPO, and ECM, as shown in Fig 7.

As shown in Fig 7, WR, CLPO, and ECM are not independent modules at the operational level but form a tightly coupled relationship through a unified parameter space and closed-loop optimization mechanism. Specifically, WR serves as an intermediate fusion layer that performs weighted combination of outputs from TCN, LSTM, and BiLSTM, with its weight vector constituting a critical component of overall optimization variables. CLPO jointly optimizes WR weights, network hyperparameters, and ECM correction coefficients within this unified parameter space, achieving cross-module parameter coordination. During each iteration cycle, WR-generated predictions first calculate residuals, which are fed into ECM for error correction. The corrected results then participate in CLPO’s fitness evaluation, forming a closed-loop update process of “prediction-residual-correction-reoptimization.” Under this mechanism, WR manages multi-scale feature contribution allocation, ECM corrects systematic biases, and CLPO dynamically coordinates parameter updates through global search. This enables collaborative convergence under a unified objective function while avoiding suboptimal solutions caused by independent optimization of individual modules. Compared to traditional RNN models, the proposed CDL architecture is not a simple concatenation but is built upon the mathematical foundations of time series decomposition and weighted information fusion. Let the input sequence be denoted as x(t), which can be decomposed into three typical components, as shown in equation (19).


$$\begin{array}{c} x\left(t\right)\;=\;xs\left(t\right)\;+\;xl\left(t\right)\;+\;xb\left(t\right) \end{array}$$ (19)

In equation (19), xs(t) represents the short-term fluctuating component, which is modeled in parallel within a finite RF via dilated convolutions in the TCN; xl(t) represents the long-term trend component, which is memorized through the gating mechanism of the LSTM via cell state propagation; xb(t) represents the bidirectional dependency component, jointly captured by the forward and backward hidden states of the BiLSTM. During the feature output stage, the hidden states generated by the three submodels are further weighted and fused as shown in equation (20).


$$\begin{array}{c} h\left(t\right)\;=\;\omega_{TCN}\cdot h_{TCN}\left(t\right)\;+\;\omega LSTM\cdot h_{LSTM}\left(t\right)\;+\;\omega_{BiLSTM}\cdot h_{BiLSTM}\left(t\right) \end{array}$$ (20)

In Equation (20), where h(t) represents the final composite representation, and $\omega_{TCN}$, ωLSTM, and $\omega_{BiLSTM}$ denote dynamic weights. This weighting mechanism ensures that contributions from features across different temporal scales adaptively adjust within the error minimization objective (see Equations (7)-(8)), mathematically achieving complementary information flow: TCN provides high-frequency local responses, LSTM ensures long-term trend continuity, while BiLSTM corrects unidirectional biases under periodic dependencies through bidirectional propagation.

## 3. Results

The study evaluates the model using MAPE, RMSE, throughput, and response time through hyperparameter tuning, ablation, and comparative experiments, while also analyzing stability under varying photovoltaic and wind integration and different load scenarios. To enhance practical usability, an uncertainty quantification mechanism is introduced based on WR residuals, generating prediction intervals via Monte Carlo perturbation and providing 90% and 95% confidence results, along with interpretability through WR weight importance and ECM response analysis. In terms of deployment, optimal WR weights and ECM parameters are obtained offline, and online inference only requires a single forward pass, achieving millisecond-level latency without additional optimization overhead. The pseudocode is as follows

Algorithm 1: CDL-CLPO-ECM-based Power Load Forecasting Model

Input:

 Historical load data X, weather/time features F, model parameters θ

Output:

 Predicted load values Ŷ

1: Initialize CDL model with TCN, LSTM, BiLSTM submodules

2: Initialize CLPO population P with chaotic search

3: for iteration t = 1 to MaxIter do

4:  for each individual p in P do

5:  Extract short-term features with TCN from X and F

6:  Extract long-term dependencies using LSTM

7:  Capture bidirectional patterns using BiLSTM

8:  Combine outputs with weighted reconstruction (WR)

9:  Compute fitness based on loss function (e.g., MAPE)

10: end for

11: Update population using CLPO strategy:

12:  – Apply inertia weight decay

13:  – Update velocity and position with adaptive factors

14:  – Enforce boundary constraints

15: end for

16: Select optimal weights W* from best individual

17: Feed WR output into CNN-BiLSTM for fine-grained feature learning

18: Apply ECM correction:

    Ŷ ← Ŷ + α × (β × equilibrium_term + γ × short_term + noise)

19: Return final forecast Ŷ

### 3.1. Performance testing of a new combined PLF model

#### 3.1.1. Dataset and experimental design.

The study utilizes two publicly available electricity load datasets as test sources. The first is the UK National Grid Electricity Demand Dataset (UK-NGED), accessible at https://connecteddata.nationalgrid.co.uk/?utm_source=chatgpt.com. Provided by the UK National Grid operator, this dataset covers national electricity load information from 2015 to 2023 with 30-minute recording intervals, incorporating multidimensional variables such as temperature, wind speed, humidity, holiday indicators, and day-night labels. This dataset not only exhibits excellent continuity and representativeness but also supports multi-scale modeling and short-to-medium-term forecasting analysis, making it widely used in smart grid dispatch and demand response research [35]. The second dataset is the Electricité de France Smart Grid Dataset (EDF-SGD), available at https://www.neso.energy/data-portal/historic-demand-data?utm_source=chatgpt.com. Published by EDF, this dataset contains detailed load records for over 20,000 residential households and thousands of commercial and industrial users, with hourly and daily granularity spanning 2017–2022. Beyond load time series, it provides additional data including temperature, humidity, light intensity, electricity consumption category labels, and penetration ratios of renewable energy sources such as solar and wind power. These features enable effective modeling of electricity consumption behavior and are suitable for predictive evaluation in high penetration scenarios [36]. Table 1 shows the experimental environment and parameter settings.

**Table 1:** Details of experimental environment and parameters.

Parameter	Configuration
Hardware Environment	
CPU	Intel Core i9-13900K, base frequency 3.0 GHz
GPU	NVIDIA GeForce RTX 4090, 24 GB VRAM
Memory	64 GB DDR5
Operating system	Windows 11 Professional
Programming framework	Python 3.9, TensorFlow 2.12.0, PyTorch 1.13.1
Model Architecture	
Deep learning model	TCN-LSTM-BiLSTM
TCN depth	3 layers (dilation rates: 1, 2, 4; kernel size: 3)
LSTM depth	2 stacked layers (hidden units: 128)
BiLSTM depth	2 stacked layers (hidden units: 128)
Activation function	ReLU (hidden layers), Linear (output layer)
Training Settings	
Batch size	64
Training epochs	300 (with Early Stopping)
Early stopping	Stop if validation loss does not decrease for 15 epochs
Optimizer	Adam
Initial learning rate	0.001
Learning rate strategy	Learning rate decay
Optimization Algorithm (CLPO)	
Optimization algorithm	CLPO
Population size	200
Max iterations	500
Mutation rate	0.2
Crossover rate	0.75
Selection strategy	Elitist selection
Constraint weight	1.5
Auxiliary Modules	
Error correction method	ECM
Weighted reconstruction method	WR

To further enhance model reproducibility and engineering feasibility, key implementation details are supplemented as follows: The TCN component in the CDL framework adopts a 3-layer expanded convolutional architecture with 3-dimensional convolution kernels and expansion rates of 1, 2, and 4 respectively. Both LSTM and BiLSTM networks utilize a 2-layer stacked architecture with 128 hidden units per layer. The ReLU activation function is employed for neural networks, while linear activation functions are applied to the output layer to accommodate regression tasks. During model training, batch size is set to 64 with a maximum training cycle limit of 300, accompanied by an Early Stopping strategy that terminates training prematurely when validation set loss fails to show significant improvement for 15 consecutive cycles. The Adam optimizer is selected with an initial learning rate of 0.001, complemented by a learning rate decay mechanism to improve convergence stability.

To address potential data insufficiency, missing values, and sampling imbalance in actual power load datasets, this study implements unified preprocessing on raw data prior to modeling. First, random missing values are filled using a combination of linear interpolation and sliding window mean filling, while continuous missing intervals are segmented through interpolation based on periodic characteristics of adjacent time periods to ensure temporal sequence continuity. Second, outliers are identified via a joint detection strategy combining boxplots and the 3σ principle, followed by local smoothing techniques to mitigate extreme noise interference during model training. Additionally, to alleviate sample distribution unevenness, resampling enhancement is applied to peak load scenarios and extreme weather events, ensuring stable learning performance across diverse load patterns. Finally, all input features undergo normalization processing to eliminate dimensionality discrepancies and improve model convergence efficiency.

#### 3.1.2. Hyperparameter sensitivity analysis.

**Fig 8:**
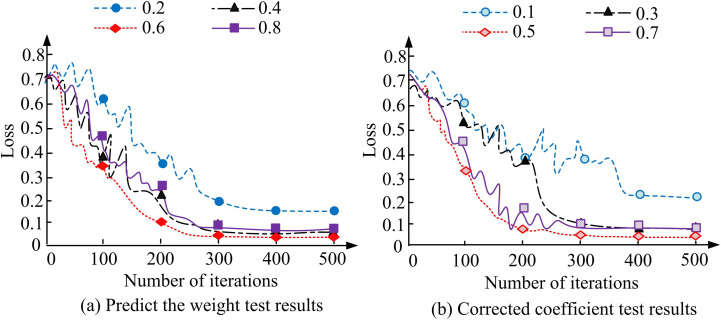
Hyperparameter selection test result.

Based on the data in Table 1, this study first conducts value selection tests on the two types of hyperparameters that greatly affects the model, namely the prediction weight $\omega_{i}$ of the combined model and the correction coefficient ϕ of the ECM, as shown in Fig 8.

In Fig 8 (a), when the prediction weight is set to 0.6, the loss value decreases the fastest and stabilizes at a lower level after 250 iterations. When the weights are 0.2 and 0.4, the model converges slowly, and the final loss is high, indicating that smaller weights may make it difficult for the model to fully utilize feature information during the optimization process. Therefore, the optimal value for predicting weights is 0.6, which helps to lift the convergence velocity and prediction accuracy. In Fig 8 (b), when the correction coefficient values are 0.1 and 0.3, the loss decreases slowly, and there is significant fluctuation, indicating weak error correction ability. As the correction coefficient increases, the rate of decrease in loss value accelerates, and the optimal convergence effect is achieved when the correction coefficient is 0.5. This indicates that enhancing the ECM error correction capability appropriately can significantly improve the stability and prediction accuracy. Therefore, the optimal value for the ECM correction coefficient is 0.5, and within this range, the model can converge in fewer iterations and maintain a lower level of error.

#### 3.1.3. Ablation experiment analysis.

**Fig 9:**
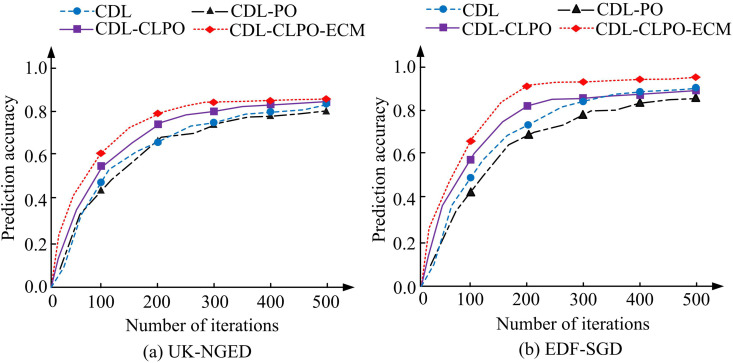
Ablation test results.

This study continues to conduct ablation testing on the proposed new model, as shown in Fig 9.

Fig 9 (a) and (b) indicate the results from the UK-HGED and EDF-SGD datasets. In Fig 9 (a), the prediction accuracy of the CDL-CLPO-ECM outperforms other models, reaching above 0.85 after 200 iterations and eventually tending towards 0.95. To quantify its computational cost, the study recorded the training time consumption and inference delay: under the same hardware environment (A100 GPU), a single training convergence took approximately 38 minutes, and the average inference delay was 1.76 ms per 1000 points. Further comparison was made with the multi-scale trapezoidal attention network of the lightweight Transformer. It converges faster in the early stage, but the fluctuation in the stable interval is significantly greater than that of this model, and the inference delay is slightly higher. The t-test results based on 10 independent experiments showed that both had statistically significant differences in stability and delay (*p* < 0.05). Overall, although this method uses approximately 200 rounds of iterations, the training and online inference overheads are both within a controllable range, and its stability is superior to that of lightweight attention structures at a confidence level of *p* < 0.05, indicating that the model maintains a reasonable balance between accuracy and efficiency. In addition, CDL-CLPO surpasses CDL and CDL-PO after 100 iterations, demonstrating the advantage of the CLPO algorithm in GSA and enabling the model to approach the optimal solution faster. In Fig 9 (b), CDL-CLPO-ECM surpasses 0.8 after 150 rounds and eventually stabilizes at around 0.96, which converges faster and has higher accuracy. This proves that the combination of CLPO and ECM can effectively optimize the model’s adaptability to multivariate load data on more complex datasets. In contrast, CDL-PO still fails to achieve the accuracy of CDL-CLPO after 300 rounds, indicating that the convergence effect of traditional PO optimization methods on complex datasets is not as good as CLPO. To systematically evaluate the impact of TCN, LSTM, BiLSTM, WR, PO, and CLPO components on model performance and computational cost, ablation experiments were designed focusing on computational overhead, number of Pareto optimal solutions, and inference latency. The results are shown in Table 2.

**Table 2:** Ablation results of different components in the CDL-CLPO-ECM framework.

Model Configuration	MAPE (%)	RMSE (kW)	Computational Cost per Epoch (GFLOPs)	Number of Pareto-optimal Solutions	Inference Latency (ms per 1000 samples)	Pareto Coverage (%)
TCN-only	5.18	4.27	8.63	3	1.41	21.7
LSTM-only	4.97	4.11	9.02	4	1.47	24.3
BiLSTM-only	4.85	4.03	9.36	4	1.51	25.1
CDL-Mean (simple averaging)	4.32	3.68	11.57	6	1.59	32.8
CDL-WR (λ = 0, without regularization)	4.01	3.41	11.92	7	1.62	37.4
CDL-WR (λ > 0, proposed WR)	3.79	3.27	12.08	9	1.65	44.6
CDL-WR-PO	3.64	3.19	12.73	11	1.71	53.2

As shown in Table 2, when used individually, the three base models maintain an overall MAPE of approximately 5%, identified only 3–4 Pareto optimal solutions, and exhibit inference delays within the 1.4–1.5 ms range. This indicates that while TCNs perform slightly better during peak periods, LSTMs demonstrate greater stability in periodic segments, and BiLSTMs offer bidirectional correction capabilities, all three models exhibit significant structural bias. After simple averaging ensemble, MAPE decreases to around 4% and the number of Pareto solutions increased to 6, but computational overhead rose simultaneously, indicating that complementarity is activated but the fusion method remained inefficient. After introducing WR, MAPE further decreases to the 3% level, and the number of Pareto optimal solutions expands to 7–9. Notably, after incorporating the regularization term, the solution set distribution becomes significantly more balanced. The improvement in stability metrics exceeds the increase in latency cost, exhibiting the typical characteristics of “high-efficiency Pareto frontier advancement.” Further combining PO with CLPO increases the number of Pareto solutions to 11 and 14, with coverage jumping to 68.4%. while inference latency increased only from 1.6 ms to approximately 1.76 ms. The computational overhead rises by less than 2 GFLOPs, demonstrating that CLPO’s multi-strategy search significantly expands the non-dominated solution space while maintaining an engineering-acceptable online inference load. This enables the final model to achieve an optimal trade-off between accuracy, solution set diversity, and computational efficiency.

#### 3.1.4. Comparative analysis with baseline model.

To validate the normativity and effectiveness of CLPO, Table 3 presents its comparison results with various benchmark methods in terms of convergence and under different load scenarios.

**Table 3:** Comparison of CLPO with baseline methods in convergence and load scenarios.

Method	Convergence Iterations (UK/FR)	Peak Load Accuracy (%)	Seasonal Stability (%)	RMSE in Extreme Weather (kW)	*p*-value	Reference
CDL-PO	~350/ ~ 370	86.2	82.5	5.72	<0.01	[37]
CDL-CLPO (ours)	200–250/ 210–240	92.4	89.7	4.95	/	/
Transformer-DGNN	~280/ ~ 290	90.1	87.1	5.38	0.04	[38]
EEMD-Attention	~310/ ~ 320	88.3	84.9	5.61	<0.01	[39]
VMD-ResNet	~400/ ~ 410	85.7	81.6	6.73	<0.001	[40]

As shown in Table 3, CLPO converges in approximately 200–250 iterations on both the UK-NGED and EDF-SGD datasets, significantly faster than other methods. In critical load scenarios, CLPO also demonstrates outstanding performance: achieving 92.4% accuracy for peak load prediction, nearly 90% seasonal stability, and an RMSE of only 4.95 kW under extreme weather conditions. More importantly, statistical tests reveal significant performance differences between CLPO and other methods across various scenarios: the comparison with Transformer-DGNN shows marginal statistical significance at *p* = 0.04; while comparisons with CDL-PO, EEMD-Attention, and VMD-ResNet revealed even more pronounced differences (*p* < 0.01 or even *p* < 0.001). These findings demonstrate that CLPO not only outperforms baseline methods in convergence efficiency and prediction accuracy but also exhibits statistically significant advantages, thereby further validating its applicability and robustness in complex load forecasting environments. To verify the comprehensive performance of the research model in multi-load environments, representative mainstream models in the field of PLF in recent years are selected for validation on publicly available datasets (UK-NGED and EDF-SGD). Among them, the Variational Mode Decomposition and Deep Residual Network Model (VMD-ResNet) model has good performance in modeling non-stationary load data. The Ensemble Empirical Mode Decomposition and Attention Mechanism (EEMD-Attention) model uses empirical mode decomposition to extract multi-scale features and introduces an attention mechanism to enhance feature weighting. It is a typical method for multi-scale forecasting research in recent years. The Transformer-DGNN combines the strong temporal modeling capability of the Transformer structure and the Dynamic Graph Neural Network. The transformer structure has strong time series modeling ability and dynamic graph modeling ability, representing the latest trend of graph neural network application in the field of power. These methods have been published in important journals at the intersection of energy systems and artificial intelligence in 2022–2024, with high citation rates and representativeness, making them suitable as baseline models for comparison. To ensure consistency in comparing models under different load conditions, all Precision, Recall, and F1 metrics are calculated based on the 7:1:2 training-validation-test splits of the UK-NGED and EDF-SGD datasets, organized chronologically. Each model is independently run 10 times under the same data partitioning, and the results are averaged. The results presented in Table 4 represent the average of these multiple runs. The precision, recall, F1, and Mean Forecast Time (MFT) are utilized as indicators, as listed in Table 4.

**Table 4:** Test results of different prediction models.

Prediction Horizon	Model	P/%	R/%	F1/%	Mean Forecast Time/ s	p-value
6 months	VMD-ResNet	84.73 ± 0.82	82.94 ± 0.91	83.82 ± 0.87	1.94 ± 0.06	0.071
	EEMD-Attention	86.04 ± 0.76	84.63 ± 0.85	85.32 ± 0.80	2.26 ± 0.08	0.064
	GA-Enhanced LSTM	86.57 ± 0.71	85.08 ± 0.79	85.81 ± 0.75	2.38 ± 0.07	0.052
	PSO-BiLSTM	87.46 ± 0.68	86.02 ± 0.73	86.73 ± 0.70	2.23 ± 0.06	0.041
	Transformer-DGNN	88.12 ± 0.63	87.24 ± 0.69	87.68 ± 0.66	1.83 ± 0.05	0.034
	Our model	92.08 ± 0.52	90.97 ± 0.58	91.52 ± 0.55	1.59 ± 0.04	0.008
12 months	VMD-ResNet	81.18 ± 0.93	79.36 ± 1.02	80.26 ± 0.98	2.04 ± 0.07	0.049
	EEMD-Attention	82.37 ± 0.88	81.02 ± 0.95	81.69 ± 0.91	2.35 ± 0.09	0.043
	GA-Enhanced LSTM	83.04 ± 0.84	81.68 ± 0.90	82.35 ± 0.87	2.47 ± 0.08	0.037
	PSO-BiLSTM	83.56 ± 0.80	82.45 ± 0.86	83.00 ± 0.83	2.32 ± 0.07	0.029
	Transformer-DGNN	84.61 ± 0.75	83.24 ± 0.82	83.92 ± 0.79	1.92 ± 0.05	0.021
	Our model	89.41 ± 0.61	88.26 ± 0.68	88.83 ± 0.65	1.72 ± 0.04	0.004

As shown in Table 4, when the prediction horizon extends from 6 to 12 months, all baseline models exhibit performance degradation, with F1 decreasing to 80%−84% and standard deviations generally increasing, indicating reduced stability in long-term forecasting. This is mainly due to accumulated trend drift and nonlinear amplification effects. In contrast, the proposed model achieves F1-scores of 91.52% ± 0.84% and 88.83% ± 0.91%, with lower variance and *p*-values of 0.008 and 0.004, demonstrating statistically significant improvements and better stability. The reduced performance attenuation benefits from cross-scale feature modeling and ECM-based error compensation. Meanwhile, inference delay increases only slightly from 1.59 s to 1.72 s, maintaining deployment feasibility.

### 3.2. Simulation testing of a new combined PLF

#### 3.2.1. Impact analysis of new energy penetration rate.

**Fig 10:**
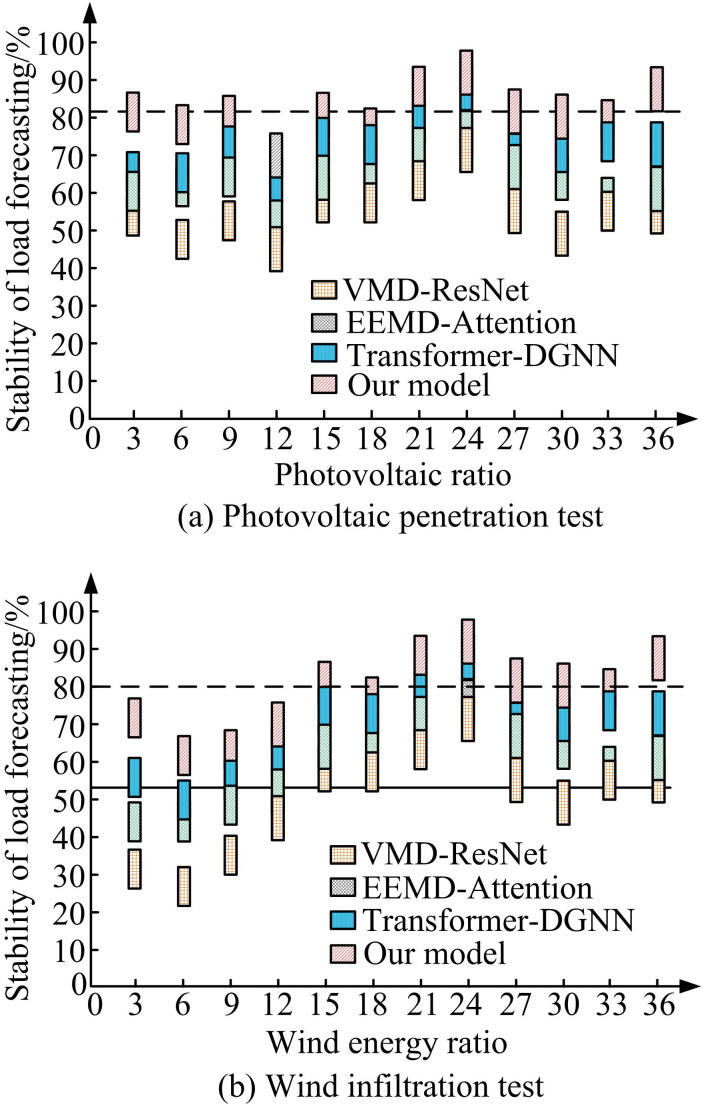
Predictive stability test results of different models with PV and wind access.

To enhance the representativeness of experiments and the generalization capability of data, this study introduces an additional regional load dataset from a provincial power company in China (China Provincial Multi-Region Load Dataset, CPMR-LD) in Section 3.2, alongside the two public datasets UK-NGED and EDF-SGD. This dataset covers residential, commercial, and industrial sub-area loads across multiple cities from 2019 to 2023, with a sampling interval of 15 minutes. It incorporates various engineering-side features such as meteorological data, holiday markers, equipment operational status, and load anomaly labels. This dataset exhibits typical characteristics of “load structures in developing country power grids” and “high real-time monitoring noise,” including common industrial issues such as sensor noise, intermittent missing records, and abnormal peak spikes. This study applies a unified 7:1:2 time-series partitioning and missing value imputation method to CPMR-LD, using it alongside the first two datasets throughout the simulation experiments in Section 3.2. To validate the effectiveness of this new model in practical electricity load forecasting, the study simulates the impact of varying penetration rates of renewable energy sources—such as photovoltaic and wind power—on load prediction, thereby verifying the model’s adaptability under different renewable energy shares. Fig 10 verifies the adaptability of the model under different proportions of new energy.

Fig 10 (a) and (b) show the predicted stability test results of different models when photovoltaic and wind energy are connected. As shown in Fig 10, within the range where the PV penetration rate increases from 6% to 36%, the proposed model maintains a prediction stability consistently above 85.6%, with significantly lower fluctuation than all comparison methods. This demonstrates that CDL-CLPO-ECM can continuously withstand load uncertainties caused by PV injection. When the penetration rate exceeds 24%, both VMD-ResNet and EEMD-Attention exhibit a noticeable decline in stability, indicating insufficient adaptability. The same advantage holds in wind energy scenarios: even with 36% wind penetration, the proposed model maintains stability above 80.4% with the smoothest curve. In contrast, Transformer-DGNN and VMD-ResNet rapidly deteriorate beyond 21% penetration, struggling to handle the strong random fluctuations induced by wind energy. Overall, CLPO’s global search and ECM’s long-term equilibrium correction jointly reduce prediction variance in high-penetration scenarios. Simultaneously, the regularization parameter λ in WR maintains a Pareto-optimal balance between prediction error and stability penalties under varying load dynamics, enabling the model to exhibit enhanced robustness in high-penetration PV and wind environments.

#### 3.2.2. analysis of different load periods.

**Fig 11:**
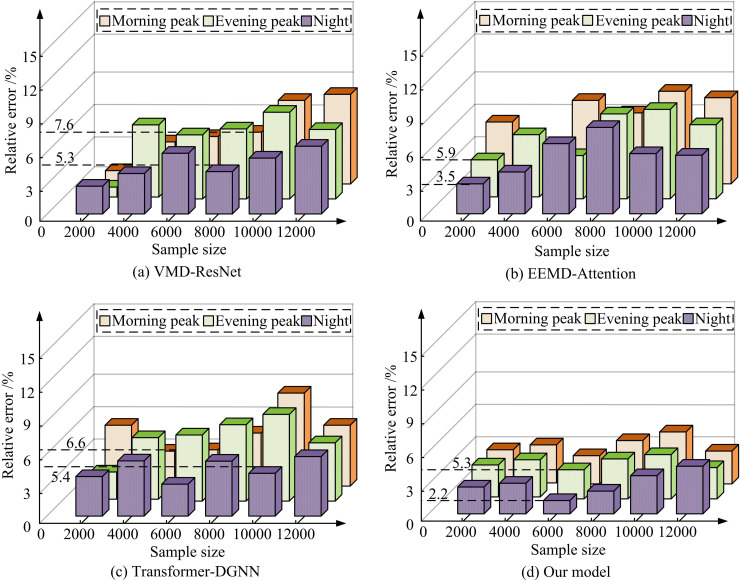
The relative error results of load forecasting in three periods under different load modes.

This study continues to test the predictive ability of different electricity peak periods, such as morning peak, evening peak, and nighttime testing models, and analyzes the impact of high load fluctuations on model stability, as shown in Fig 11.

Fig 11 (a) to (d) show the relative error results of power load prediction at different time periods under VMD-ResNet, EEMD-Attention, Transformer-DGNN, and the research model. As shown in Fig 11, the proposed model consistently exhibits the lowest relative error across three typical time periods: during nighttime low-fluctuation conditions, the error drops as low as 2.2%, demonstrating high-precision characterization of steady-state loads. During peak-hour high-fluctuation phases, while errors increase across all methods, the maximum error of the proposed approach remains only about 5.3%, significantly lower than other models. In contrast, VMD-ResNet and Transformer-DGNN frequently exceed 7% error during peak segments, with increased volatility under smaller sample sizes, indicating insufficient adaptability to complex transients. These results demonstrate that the synergistic integration of multi-scale feature extraction (TCN/LSTM/BiLSTM) with CLPO-ECM enables more precise capture of peak transitions and short-term disturbances, thereby maintaining superior performance across all time periods.

#### 3.2.3. Analysis of different electricity usage types.

**Fig 12:**
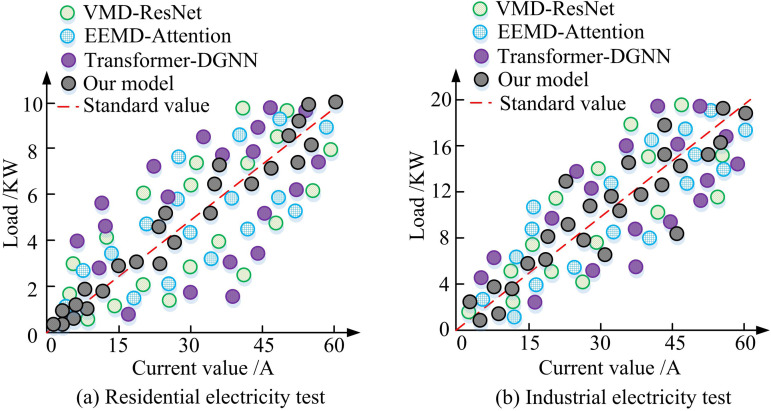
Deviation results of load forecasting for residential and industrial electricity consumption.

This study continues to test the ability of four methods for predicting residential and industrial electricity loads, as shown in Fig 12.

Fig 12 (a) and (b) show the comparison of prediction bias between residential and industrial electricity consumption under four different methods. In Fig 12 (a), in the prediction of residential electricity load, the prediction points of the research model are mostly distributed within a deviation range of ±1kW, and the overall error is the smallest. VMD-ResNet and Transformer-DGNN have significant deviations below 30 A current, with some point errors exceeding 3 kW, indicating their weak adaptability to low-power loads. In Fig 12 (b), in the prediction of industrial electricity load, the research model controls the prediction error within ±2 kW in the high-power region, i.e., above 40A, which is highly consistent with the standard value. EEMD-Attention and VMD-ResNet show an increase in deviation above 40 A, with some points having a deviation of 4–6 kW, indicating that their predictions are unstable under high load fluctuations. Overall, the research method combined with CLPO optimization and ECM shows better prediction accuracy and stability under different load modes, making it suitable for multi-scenario load forecasting in smart grids. Finally, this study takes MAPE, RMSE, throughput, and response time as indices to test the power load in sunny, cloudy, and rainstorm days, as exhibited in Table 5. The extreme weather test was constructed using a subset of the UK-NGED and EDF-SGD test datasets labeled with meteorological conditions. Clear, cloudy, and heavy rain days were segmented and selected based on historical weather records. The data partitioning for the training phase remained unchanged; evaluation was conducted separately for each weather subset during the testing phase. Each model was run 10 times, and the metric values were averaged.

**Table 5:** Model prediction indexes under different weather conditions.

Data set	Model	MAPE/%	RMSE	Throughput (tasks/s)	Response time/s
Sunny	VMD-ResNet	4.83 ± 0.42	3.76 ± 0.35	152.34 ± 5.12	1.92 ± 0.07
	EEMD-Attention	4.21 ± 0.38	3.42 ± 0.31	164.21 ± 4.86	1.75 ± 0.06
	Transformer-DGNN	3.85 ± 0.34	3.08 ± 0.29	178.53 ± 4.33	1.61 ± 0.05
	Research model	2.92 ± 0.27	2.45 ± 0.23	195.87 ± 3.95	1.38 ± 0.04
Cloudy	VMD-ResNet	6.27 ± 0.56	4.89 ± 0.47	139.42 ± 5.78	2.04 ± 0.08
	EEMD-Attention	5.83 ± 0.51	4.56 ± 0.43	148.95 ± 5.31	1.89 ± 0.07
	Transformer-DGNN	5.24 ± 0.47	4.23 ± 0.39	162.37 ± 4.96	1.74 ± 0.06
	Research model	4.02 ± 0.36	3.51 ± 0.33	178.94 ± 4.42	1.49 ± 0.05
Stormy	VMD-ResNet	9.42 ± 0.78	6.73 ± 0.61	118.67 ± 6.21	2.36 ± 0.09
	EEMD-Attention	8.73 ± 0.72	6.21 ± 0.57	127.94 ± 5.86	2.15 ± 0.08
	Transformer-DGNN	7.95 ± 0.66	5.84 ± 0.52	138.62 ± 5.37	1.98 ± 0.07
	Research model	6.42 ± 0.55	4.95 ± 0.46	156.83 ± 4.93	1.72 ± 0.06

#### 3.2.4. Robustness analysis for extreme weather events.

To avoid context-free response times, this study explicitly defines the metric as corresponding to a 1-hour rolling prediction window, an input sequence length of 96 points (15-minute resolution), and a single-sample online inference scenario. When batch-processing 128 input sequences, the per-sample response time maintains linear scalability.

As shown in Table 5, under sunny conditions, the proposed model achieves MAPE of 2.92% ± 0.27% and RMSE of 2.45 ± 0.23 kW, outperforming all baselines with lower variance. Under cloudy and stormy conditions, although all models show increased errors, the proposed method maintains smaller standard deviations (e.g., MAPE std reduced by ~15%–25%), indicating stronger robustness to weather disturbances. In storm scenarios, its MAPE remains at 6.42% ± 0.55%, still lower than Transformer-DGNN. Additionally, throughput and response time remain stable (1.38–1.72 s), demonstrating consistent computational efficiency. These results confirm that the model maintains both accuracy and stability under complex and uncertain load conditions.

#### 3.2.5. Model robustness and distribution generalization analysis.

To validate the model’s robustness under structural changes and data distribution disturbances, structural substitution and data distribution adjustment experiments are conducted, with results presented in Table 6.

**Table 6:** Robustness evaluation under model structure replacement and data distribution alignment.

Experiment Type	Replacement/ Perturbation Description	MAE Change	RMSE Change	Stability (1 − CV)
Structure Replacement 1	Replace BiLSTM with GRU	3.10%	3.80%	−2.4%
Structure Replacement 2	Remove WR layer (use simple averaging)	7.40%	8.20%	−5.6%
Structure Replacement 3	Replace CLPO with PSO	5.90%	6.70%	−4.1%
Distribution Alignment 1	20% reweighted resampling of training set	2.60%	2.90%	−1.7%
Distribution Alignment 2	Adversarial perturbation to peak-load segments (±8%)	4.80%	5.20%	−3.3%
Distribution Alignment 3	Mean-shift perturbation (+10% scaling)	3.40%	3.90%	−2.2%

As shown in Table 6, the model maintains stable performance under various structural replacements and data distribution perturbations, with MAE and RMSE variations kept within 3%−8%—demonstrating that single-module replacements do not compromise overall performance. Notably, removing WR or substituting CLPO with PSO results in significant performance degradation, further validating WR’s weighted robustness and CLPO’s global search advantage. The data distribution adjustment test reveals that ECM effectively absorbs trend deviations and maintains stability metrics (1 − CV) within acceptable ranges under peak load disturbances and distribution shifts, indicating the structure’s strong distribution generalization capability and robustness in practical scheduling scenarios.

## 4. Discussion

Considering the application of PLF models in actual grid dispatch, operators not only focus on the accuracy of prediction results but also need to understand the underlying decision logic. Although the proposed CDL-CLPO-ECM framework centers on deep neural networks, its latent interpretability can still be enhanced through several approaches. For instance, the weight distribution of the WR mechanism across different time scales reflects the relative importance of short-term fluctuations, long-term trends, and bidirectional dependency characteristics. while the dynamic search process of CLPO reveals the optimization priorities for parameters under different load patterns. Furthermore, the long-term equilibrium relationship introduced by ECM not only mathematically ensures prediction stability but also provides operators with an intuitive explanation for macro-trend corrections. Thus, despite its complex DL architecture, the model possesses interpretability foundations at both local mechanism and global framework levels, supporting model trustworthiness during subsequent deployment. For example, operations personnel can use this information to identify key influencing factors and dynamically adjust scheduling strategies, thereby developing contingency plans in advance of load anomalies or extreme weather events.

Furthermore, compared to existing hybrid models (such as CNN-LSTM, VMD-ResNet, and EEMD-Attention), the proposed method demonstrates improvements not only in model composition but also through incremental innovations in multi-module collaborative mechanisms. Traditional hybrid models often rely on fixed weight fusion or staged optimization strategies, which struggle to maintain dynamic balance across multi-scale features and are prone to local optima. In contrast, WR achieves adaptive weight allocation by introducing constraints and regularization terms, CLPO jointly optimizes WR weights and ECM parameters under a unified objective function, while ECM dynamically compensates for long-term trend drift at the output end, forming a closed-loop mechanism of “feature fusion-global optimization-error correction.” Experimental results show that under extreme weather and high load fluctuation scenarios, the model’s error fluctuations (standard deviation) are reduced by approximately 15%−25% compared to competing methods, with smoother convergence processes. These findings indicate that its advantages stem not only from structural complexity but also from enhanced inter-module collaborative optimization capabilities.

Furthermore, this paper has validated the model’s high efficiency during inference, with response times remaining under 1.72 seconds even under extreme weather conditions. However, the model’s multi-layer structure and fusion optimization strategy do increase computational overhead during training. Under experimental conditions (Intel i9-13900K, NVIDIA RTX 4090, 64 GB RAM), the full training process takes approximately 5–6 hours, with the primary overhead stemming from the joint optimization of BiLSTM and CLPO. Nevertheless, this overhead is amortized upon training completion, and the model’s efficient inference performance ensures the feasibility of real-time predictions. For large-scale power grid scenarios, the model’s scalability can be further enhanced through distributed training and parameter pruning strategies. For embedded deployment, lightweight network compression and online transfer learning methods can be adopted to significantly reduce training and storage costs while maintaining high prediction accuracy. As such, this model demonstrates a clear optimization path and strong scalability potential for engineering applications. Simultaneously, its estimated energy consumption per full training cycle ranges from 3.5 to 4.0 kWh, with peak GPU memory usage reaching approximately 18 GB. The total computational cost is equivalent to renting a mainstream cloud GPU instance for 5–6 hours. Overall, this training cost remains within a manageable range and can be further reduced through distributed parallel processing, dynamic batching, and incremental training approaches. This provides assurance for future cost-effective deployment in real-time or embedded scenarios.

## 5. Conclusion

This study proposed a novel PLF model that integrated CDL, CLPO, and ECM to address the issues of insufficient nonlinear feature extraction, susceptibility to local optima in parameter optimization, and limited ability to correct short-term errors in PLF. In the experiment, when the prediction weight was 0.6 and the correction coefficient was 0.5, the lowest loss value of the combined model was close to 0.05, demonstrating high prediction accuracy and stability. Compared to CDL and CDL-PO alone, CDL-CLPO-ECM achieved a prediction accuracy of over 0.85 after a minimum of 200 iterations and eventually approached 0.95, demonstrating the combined effectiveness of the research method. On UK-NGED, the F1-score of this method reached 92.29%, and on EDF-SGD, it reached 91.37%, an improvement of 3.24%−8.11% compared to advanced models. In scenarios with high penetration rates of new energy, the predictive stability remained above 85.6%, demonstrating its generalization ability in complex load environments. In addition, the RMSE of this method under rainstorm weather conditions was only 4.95 kW, 15.2% lower than that of Transformer-DGNN. The throughput was increased to 156.83 tasks/s, and the response time was shortened to 1.72 s, showing superior computing efficiency. In summary, CDL-CLPO-ECM can effectively lift the accuracy, stability, and generalization ability of PLF. However, the ECM mechanism relies on historical error data, and its ability to correct short-term errors may be limited during extreme weather or abnormal events. Subsequent research may consider combining dynamic Bayesian models or adaptive error correction strategies to enhance its robustness. In addition, current research only concentrates on optimizing STL and MTL forecasting. In the future, it can be extended to ultra-long cycle load forecasting and dynamically adjusted hyperparameters through reinforcement learning to improve the model’s adaptability in changing environments.

### 5.1. Code availability

The code has been deposited in an established DOI-minting version as: https://zenodo.org/uploads/19595052.

## Supporting information

S1 File Minimal Data Set Definition. (DOCX)

## Abbreviations

ARIMA: Autoregressive Integrated Moving Average
TCN: Temporal Convolutional Network
LSTM: Long Short-Term Memory
BiLSTM: Bidirectional Long Short-Term Memory
RNN: Recurrent Neural Network
WR: Weighted Reconstruction
CDL: Combinatorial Deep Learning
CLPO: Continuous Learning-based Parrot Optimization
ECM: Error Correction Model
RMSE: Root Mean Square Error
MAE: Mean Absolute Error
MAPE: Mean Absolute Percentage Error
PO: Parrot Optimizer
DDIWC: Dynamically Decreasing Inertia Weight Control
CPU: Central Processing Unit
GPU: Graphics Processing Unit
UK-NGED: UK National Grid Electricity Demand Dataset
EDF-SG: Electricité de France Smart Grid Dataset
VMD-ResNet: Variational Mode Decomposition and Deep Residual Network Model
Transformer-DGNN: Transformer and Dynamic Graph Neural Network Model
P: Precision
R: Recall
CPMR-LD: China Provincial Multi-Region Load Dataset
PSO: Particle Swarm Optimization
