# Supplementary material for: Power load forecasting combining deep learning models and improved CLPO algorithm
Source: PLoS One. 2026 Jun 29;21(6):e0351428. doi: 10.1371/journal.pone.0351428 (PMC13313379; doi:10.1371/journal.pone.0351428)
Supplement: S1 File — (DOCX) [file pone.0351428.s001.docx]

**Figure 8.Hyperparameter selection test result**

| Parameter | Converged Loss (a) Weight Test | Convergence Iterations (a) | Converged Loss (b) Correction Coefficient | Convergence Iterations (b) |
| --- | --- | --- | --- | --- |
| 0.2 | 0.28 | 350 | 0.32 | 400 |
| / |  | / | 0.08 | 220 |
| 0.4 | 0.12 | 220 | / | / |
| 0.5 | / | / | 0.02 | 180 |
| 0.6 | 0.02 | 150 | / | / |
| 0.7 | / | / | 0.03 | 200 |
| 0.8 | 0.01 | 180 | / | / |

**Figure 9.Ablation test results**

| Dataset | Method | Accuracy 200 iters | Accuracy 500 iters | Convergence Iterations | Final Accuracy |
| --- | --- | --- | --- | --- | --- |
| UK-NGED | CDL | 0.72 | 0.83 | 00 | 0.83 |
|  | CDL-PO | 0.65 | 0.82 | 450 | 0.82 |
|  | CDL-CLPO | 0.78 | 0.90 | 300 | 0.90 |
|  | CDL-CLPO-ECM | 0.85 | 0.95 | 200–250 | 0.95 |
| EDF-SGD | CDL | 0.70 | 0.87 | 400 | 0.87 |
|  | CDL-PO | 0.62 | 0.85 | 450 | 0.85 |
|  | CDL-CLPO | 0.80 | 0.91 | 300 | 0.91 |
|  | CDL-CLPO-ECM | 0.88 | 0.96 | 200–250 | 0.96 |

**Figure 10.Predictive stability test results of different models with PV and wind access**

| Penetration ratio (%) | VMD-ResNet | EEMD-Attention | Transformer-DGNN | Our model |
| --- | --- | --- | --- | --- |
| Photovoltaic test | / | / | / | / |
| 6 | 55–70 | 60–75 | 68–78 | 80–86 |
| 12 | 52–68 | 58–73 | 66–77 | 82–88 |
| 18 | 50–65 | 56–72 | 64–75 | 83–87 |
| 24 | 45–60 | 52–68 | 61–73 | 82–86 |
| 30 | 43–58 | 50–66 | 60–72 | 81–85 |
| 36 | 40–55 | 48–65 | 58–71 | 80–84 |
| Wind test | / | / | / | / |
| 6 | 48–62 | 53–68 | 63–74 | 78–84 |
| 12 | 45–60 | 51–66 | 61–72 | 80–86 |
| 18 | 42–58 | 49–64 | 60–71 | 81–87 |
| 24 | 40–55 | 47–63 | 58–70 | 80–85 |
| 30 | 38–52 | 46–61 | 57–69 | 79–84 |
| 36 | 35–50 | 44–60 | 55–68 | 78–82 |

**Figure 11. The relative error results of load forecasting in three periods under different load modes**

| Sample size | Load mode | VMD-ResNet | EEMD-Attention | Transformer-DGNN | Our model |
| --- | --- | --- | --- | --- | --- |
| 2000 | Morning | 6.8 | 6.5 | 6.1 | 3.2 |
|  | Evening | 7.1 | 6.7 | 6.3 | 3.5 |
|  | Night | 5.2 | 4.9 | 5 | 2.2 |
| 4000 | Morning | 8.3 | 7.9 | 7.2 | 4 |
|  | Evening | 8.6 | 8.1 | 7.4 | 4.2 |
|  | Night | 6.1 | 5.7 | 5.5 | 2.8 |
| 6000 | Morning | 9.2 | 8.8 | 8 | 4.5 |
|  | Evening | 9.5 | 9 | 8.3 | 4.7 |
|  | Night | 6.8 | 6.4 | 6 | 3.2 |
| 8000 | Morning | 10.3 | 9.8 | 8.8 | 5 |
|  | Evening | 10.6 | 10 | 9 | 5.2 |
|  | Night | 7.2 | 6.9 | 6.5 | 3.7 |
| 10000 | Morning | 11.2 | 10.7 | 9.5 | 5.6 |
|  | Evening | 11.5 | 11 | 9.7 | 5.8 |
|  | Night | 7.6 | 7.3 | 6.9 | 4 |
| 12000 | Morning | 12.5 | 12 | 10.8 | 6 |
|  | Evening | 12.8 | 12.3 | 11 | 6.2 |
|  | Night | 8.2 | 7.8 | 7.3 | 4.3 |

**Figure** **12. Deviation results of load forecasting for residential and industrial electricity consumption**

| Scenario | Model | MAE (kW) | RMSE (kW) | Max Deviation (kW) | Low load (0–20A) | Medium load (20–40A) | High load (40–60A) |
| --- | --- | --- | --- | --- | --- | --- | --- |
| Residential | VMD-ResNet | 1.85 | 2.47 | ±3.5 | 1.2 | 2 | 3.5 |
|  | EEMD-Attention | 1.62 | 2.18 | ±3.0 | 1 | 1.8 | 3 |
|  | Transformer-DGNN | 1.4 | 1.96 | ±2.8 | 0.9 | 1.6 | 2.8 |
|  | Our model | 0.92 | 1.35 | ±1.5 | 0.6 | 1 | 1.5 |
| Industrial | VMD-ResNet | 3.25 | 4.18 | ±6.0 | 2 | 4 | 6 |
|  | EEMD-Attention | 2.87 | 3.75 | ±5.5 | 1.8 | 3.5 | 5.5 |
|  | Transformer-DGNN | 2.41 | 3.12 | ±4.5 | 1.5 | 3 | 4.5 |
|  | Our model | 1.36 | 1.98 | ±2.0 | 0.9 | 1.5 | 2 |
