# Supplementary material for: Power load forecasting combining deep learning models and improved CLPO algorithm
Source: PLoS One. 2026 Jun 29;21(6):e0351428. doi: 10.1371/journal.pone.0351428 (PMC13313379; doi:10.1371/journal.pone.0351428)
Supplement: Attachment — Submitted filename: Response to comments.docx [file pone.0351428.s003.docx]

Review Comments to the Author

Please use the space provided to explain your answers to the questions above. You may also include additional comments for the author, including concerns about dual publication, research ethics, or publication ethics. (Please upload your review as an attachment if it exceeds 20,000 characters)

Reviewer #1:

While the technical depth and innovation are commendable, a few minor changes are recommended to strengthen the manuscript.

First, given the complexity of the model, the paper would benefit from a short discussion on model interpretability or potential explainability strategies, especially since power grid operators may need insights into prediction rationale.

Reply: Thank you very much for your comments. The interpretability of the model has been fully discussed, as follows:

Considering the application of power load forecasting models in actual grid dispatch, operators not only focus on the accuracy of prediction results but also need to understand the underlying decision logic. Although the proposed CDL-CLPO-ECM framework centers on deep neural networks, its latent interpretability can still be enhanced through several approaches. For instance, the weight distribution of the weighted reconstruction mechanism across different time scales reflects the relative importance of short-term fluctuations, long-term trends, and bidirectional dependency characteristics. while the dynamic search process of CLPO reveals the optimization priorities for parameters under different load patterns. Furthermore, the long-term equilibrium relationship introduced by ECM not only mathematically ensures prediction stability but also provides operators with an intuitive explanation for macro-trend corrections. Thus, despite its complex deep learning architecture, the model possesses interpretability foundations at both local mechanism and global framework levels, supporting model trustworthiness during subsequent deployment. For example, operations personnel can use this information to identify key influencing factors and dynamically adjust scheduling strategies, thereby developing contingency plans in advance of load anomalies or extreme weather events.

Second, although the paper discusses inference efficiency, there is limited commentary on training time or computational overhead introduced by the model's multi-layered structure. Clarifying this aspect would improve understanding of its scalability for real-time or embedded deployment.

Reply: Thank you for your comments. The training time or computational cost caused by the multi-layer structure of the model have been discussed, as follows:

Furthermore, this paper has validated the model's high efficiency during inference, with response times remaining under 1.72 seconds even under extreme weather conditions. However, the model's multi-layer structure and fusion optimization strategy do increase computational overhead during training. Under experimental conditions (Intel i9-13900K, NVIDIA RTX 4090, 64 GB RAM), the full training process takes approximately 5–6 hours, with the primary overhead stemming from the joint optimization of BiLSTM and CLPO. Nevertheless, this overhead is amortized upon training completion, and the model's efficient inference performance ensures the feasibility of real-time predictions. For large-scale power grid scenarios, the model's scalability can be further enhanced through distributed training and parameter pruning strategies. For embedded deployment, lightweight network compression and online transfer learning methods can be adopted to significantly reduce training and storage costs while maintaining high prediction accuracy. As such, this model demonstrates a clear optimization path and strong scalability potential for engineering applications. Simultaneously, its estimated energy consumption per full training cycle ranges from 3.5 to 4.0 kWh, with peak GPU memory usage reaching approximately 18 GB. The total computational cost is equivalent to renting a mainstream cloud GPU instance for 5 to 6 hours. Overall, this training cost remains within a manageable range and can be further reduced through distributed parallel processing, dynamic batching, and incremental training approaches. This provides assurance for future cost-effective deployment in real-time or embedded scenarios.

Third, the feature set focuses on technical inputs such as load, weather, and time indicators. The inclusion or future consideration of behavioral or economic variables could be acknowledged, as such factors are increasingly used in modern load forecasting.

Reply: Thank you for your comments. The objective of this research is to construct and validate a methodological framework integrating CDL, CLPO, and ECM, with a core focus on algorithmic feature extraction and optimization mechanisms. While incorporating behavioral or economic variables holds value, their acquisition relies on long-term user research and cross-domain data integration—beyond the scope of this manuscript's experimental design and data availability. To ensure research reproducibility and fair comparability, this manuscript exclusively utilizes load, weather, and time-related technical metrics as inputs.

Future work will proceed under clearly defined data conditions, technical pathways, and research objectives. For instance, after acquiring multi-source data such as 15–60 minute user load from smart meters, demand response event records, time-of-use or real-time electricity prices, and regional macroeconomic panel data, data integration and protection will be achieved using compliant data sharing technologies like federated learning and differential privacy. Methodologically, exogenous features such as price elasticity, load shifting indices, holiday/event behavioral embeddings, and lagged economic variables will be incorporated. An exogenous variable gating or attention subnetwork will be constructed, and the error correction model will be extended to a multivariate form. Concurrently, an improved parrot optimization algorithm will jointly optimize model hyperparameters and variable subsets. Finally, we systematically evaluate model gains under typical scenarios like electricity price perturbations, holiday anomalies, and high renewable energy penetration. Through ablation and causal constraint experiments, concept drift detection, and online incremental learning, we validate the enhanced prediction accuracy, robustness, and real-time performance achieved by incorporating behavioral and economic variables, confirming their engineering applicability.

Finally, minor language revisions are suggested to enhance readability and clarity, especially in sections describing algorithmic flow and dataset characteristics.

Reply: Thank you for your comments. The algorithm processes and the data set features have been re-explained. As follows:

**The algorithm processes:**

As shown in Figure 4, the PO algorithm first initializes the search agents and calculates fitness to measure the quality of candidate solutions. Subsequently, solutions are ranked based on fitness results, and corresponding update strategies are selected. During the strategy phase, each search agent randomly adopts one of the update methods [27]. Foraging behavior explores high-fitness regions within the environment, adjusting positions via the fitness gradient as demonstrated in Equation (9) [28-29].

The stay behavior maintains the current solution to preserve local optima and triggers fine-tuning when fitness fluctuations are small, as calculated by Equation (10).

Interaction behavior adjusts positions based on neighborhood information, enabling information sharing among individuals and enhancing global search capabilities, as calculated by Equation (11).

After completing the position update, the algorithm determines whether the maximum iteration count has been reached. If the condition is met, the optimal solution is output; otherwise, the process enters the boundary control phase to ensure the agent remains within the feasible solution space and continues iteration.

**The data set features:**

The study utilized two publicly available electricity load datasets as test sources. The first is the UK National Grid Electricity Demand Dataset (UK-NGED), provided by the UK National Grid operator. It covers national electricity load information from 2015 to 2023 at 30-minute intervals and includes multidimensional variables such as air temperature, wind speed, humidity, holiday indicators, and day/night labels. This dataset not only exhibits excellent continuity and representativeness but also supports multi-scale modeling and short-to-medium-term forecasting analysis, making it widely used in smart grid dispatch and demand response research [35]. The second dataset is the Électricité de France Smart Grid Dataset (EDF-SGD), released by Électricité de France. It contains detailed load records for over 20,000 residential customers and thousands of commercial and industrial users, with data granularity at the hourly and daily levels, spanning the period from 2017 to 2022. Beyond load time series, this dataset provides temperature, humidity, solar irradiance, electricity usage type labels, and penetration rates of renewable energy sources like photovoltaics and wind power. It effectively supports modeling electricity consumption behavior and is suitable for forecasting evaluations in high-penetration scenarios [36].

Reviewer #2:

This paper presents a power load forecasting method that combines deep learning models (TCN-LSTM-BiLSTM) with an improved Continuous Learning-based Parrot Optimization (CLPO) algorithm and Error Correction Model (ECM) to enhance prediction accuracy in complex load environments. The authors propose a Combined Deep Learning (CDL) framework that uses weighted reconstruction to integrate multiple model outputs and applies parrot optimization for hyperparameter tuning and joint error correction for improved long-term forecasting performance. While the work addresses a relevant problem in power system management, it suffers from fundamental methodological flaws and lacks rigorous theoretical foundations that severely compromise its scientific contribution.

Comment 1: The theoretical justification for combining TCN, LSTM, and BiLSTM architectures lacks rigorous mathematical foundation and appears to be an ad-hoc integration of existing techniques without demonstrating fundamental advantages over individual components or established ensemble methods. The authors claim that CDL combines parallel computing capability of TCN with long memory advantages of LSTM and bidirectional characteristics of BiLSTM, but they do not provide mathematical analysis of how these architectures complement each other, how information flows between different temporal modeling mechanisms, or why this specific combination is optimal for power load forecasting compared to other sequence modeling approaches.

Reply: Thank you very much for your comments. Regarding the theoretical foundation and mathematical support for the CDL (TCN+LSTM+BiLSTM) combined architecture, the following supplementary explanation is provided:

This model does not simply stack the three networks. Instead, it employs a mathematical framework based on time series decomposition and weighted information fusion to decompose load data into three typical components: short-term fluctuations, long-term trends, and bidirectional dependencies. These components are then adaptively fused under a loss minimization objective. The modification is as follows:

Compared to traditional RNN models, the proposed CDL architecture is not a simple concatenation but is built upon the mathematical foundations of time series decomposition and weighted information fusion. Let the input sequence be denoted as x(t), which can be decomposed into three typical components, as shown in Equation (19).

$x\left(t\right)\text{ }=\text{ }xs\left(t\right)\text{ }+\text{ }xl\left(t\right)\text{ }+\text{ }xb\left(t\right)$ (19)

In Equation (19), $xs\left(t\right)$ represents the short-term fluctuating component, which is modeled in parallel within a finite receptive field via dilated convolutions in the TCN; $xl\left(t\right)$ represents the long-term trend component, which is memorized through the gating mechanism of the LSTM via cell state propagation; $xb\left(t\right)$ represents the bidirectional dependency component, jointly captured by the forward and backward hidden states of the BiLSTM. During the feature output stage, the hidden states generated by the three submodels are further weighted and fused as shown in Equation (20).

$h\left(t\right)\text{ }=\text{ }\omega_{TCN}\cdot h_{TCN}\left(t\right)\text{ }+\text{ }\omega{}_{LSTM}\cdot h_{LSTM}\left(t\right)\text{ }+\text{ }\omega_{BiLSTM}\cdot h_{BiLSTM}\left(t\right)$ (20)

In equation (20), where In equation (20), where A represents the final composite representation, and $\omega_{TCN}$, $\omega{}_{LSTM}$, and $\omega_{BiLSTM}$ denote dynamic weights. This weighting mechanism ensures that contributions from features across different temporal scales adaptively adjust within the error minimization objective (see Equations (7)-(8)), mathematically achieving complementary information flow: TCN provides high-frequency local responses, LSTM ensures long-term trend continuity, while BiLSTM corrects unidirectional biases under periodic dependencies through bidirectional propagation. represents the final composite representation, and B, C, and D denote dynamic weights. This weighting mechanism ensures that contributions from features across different temporal scales adaptively adjust within the error minimization objective (see Equations (7)-(8)), mathematically achieving complementary information flow: TCN provides high-frequency local responses, LSTM ensures long-term trend continuity, while BiLSTM corrects unidirectional biases under periodic dependencies through bidirectional propagation.

Comment 2: The experimental validation methodology exhibits critical limitations that undermine the credibility of the reported performance improvements, particularly the absence of comprehensive baseline comparisons and inadequate statistical validation across diverse load scenarios. The authors claim prediction accuracy exceeding 0.85 after 200 iterations but do not provide detailed experimental protocols, cross-validation procedures, or comparison with recent state-of-the-art power forecasting methods including transformer-based approaches, attention mechanisms, or other hybrid optimization techniques that are directly relevant to their architectural choices.

Reply: Thank you for your comments. The third section of the manuscript has been revised, with a new Table 2 illustrating the comparison results between CLPO and State-of-the-art power forecasting methods (including transformer-based approaches, attention mechanisms, or other methods) in terms of convergence and different load scenarios. The statistical test p-values have also been validated. Additionally, technical aspects such as Transformer models and attention mechanisms have been compared. The revisions are as follows:

To validate the normativity and effectiveness of CLPO, Table 2 presents its comparison results with various benchmark methods in terms of convergence and under different load scenarios.

**Table 2. Comparison of CLPO with baseline methods in convergence and load scenarios**

| Method | Convergence Iterations (UK/FR) | Peak Load Accuracy (%) | Seasonal Stability (%) | RMSE in Extreme Weather (kW) | *p*-value | Reference |
| --- | --- | --- | --- | --- | --- | --- |
| CDL-PO | ~350 / ~370 | 86.2 | 82.5 | 5.72 | <0.01 | [37] |
| CDL-CLPO (ours) | 200–250 / 210–240 | 92.4 | 89.7 | 4.95 | / | / |
| Transformer-DGNN | ~280 / ~290 | 90.1 | 87.1 | 5.38 | 0.04 | [38] |
| EEMD-Attention | ~310 / ~320 | 88.3 | 84.9 | 5.61 | <0.01 | [39] |
| VMD-ResNet | ~400 / ~410 | 85.7 | 81.6 | 6.73 | <0.001 | [40] |

As shown in Table 2, CLPO converges in approximately 200-250 iterations on both the UK-NGED and EDF-SGD datasets, significantly faster than other methods. In critical load scenarios, CLPO also demonstrates outstanding performance: achieving 92.4% accuracy for peak load prediction, nearly 90% seasonal stability, and an RMSE of only 4.95 kW under extreme weather conditions. More importantly, statistical tests reveal significant performance differences between CLPO and other methods across various scenarios: the comparison with Transformer-DGNN shows marginal statistical significance at *p*=0.04; while comparisons with CDL-PO, EEMD-Attention, and VMD-ResNet revealed even more pronounced differences (*p*<0.01 or even *p*<0.001). These findings demonstrate that CLPO not only outperforms baseline methods in convergence efficiency and prediction accuracy but also exhibits statistically significant advantages, thereby further validating its applicability and robustness in complex load forecasting environments.

Comment 3: The Continuous Learning-based Parrot Optimization algorithm lacks detailed algorithmic specification and convergence analysis that are essential for understanding its practical implementation and theoretical guarantees. Pl follow the work The authors mention using CLPO for dynamic weight adjustment and parameter optimization but do not provide mathematical formulation of the optimization objective function, convergence criteria, or analysis of how the parrot optimization metaheuristic specifically adapts to the power load forecasting problem. The relationship between optimization iterations and forecasting performance improvement is not rigorously established.

Reply: Thank you for your comments. The algorithm specification and convergence analysis for the Parrot Optimization Algorithm based on continuous learning have been supplemented. This includes explanations of the mathematical formulation of the optimization objective function, convergence criteria, and the approach or principles by which the Parrot Optimization Agent heuristic adapts to power load forecasting problems. Additionally, the relationship between optimization iterations and improvements in forecasting performance is elucidated. Detailed modifications are as follows:

**Algorithm specification and convergence analysis of parrot optimization algorithm based on continuous learning：**

As shown in Table 2, CLPO converges in approximately 200-250 iterations on both the UK-NGED and EDF-SGD datasets, significantly faster than other methods. In critical load scenarios, CLPO also demonstrates outstanding performance: achieving 92.4% accuracy for peak load prediction, nearly 90% seasonal stability, and an RMSE of only 4.95 kW under extreme weather conditions. More importantly, statistical tests reveal significant performance differences between CLPO and other methods across various scenarios: the comparison with Transformer-DGNN shows marginal statistical significance at *p*=0.04; while comparisons with CDL-PO, EEMD-Attention, and VMD-ResNet revealed even more pronounced differences (*p*<0.01 or even *p*<0.001). These findings demonstrate that CLPO not only outperforms baseline methods in convergence efficiency and prediction accuracy but also exhibits statistically significant advantages, thereby further validating its applicability and robustness in complex load forecasting environments.

**The mathematical formula of the objective function and convergence criterion:**

To further enhance standardization, the optimization process of CLPO is formalized into the following objective function as shown in Equation (15).

$J(\omega,\theta)=\sum_{t} \left(y_{t}-\hat{y}_{t}\left(\omega,\theta\right)^{2}+\lambda\left\|\omega\right\|\right)^{2}$ (15)

In Equation (15), $y_{t}$ represents the actual load, while $\hat{y}_{t}\left(\omega,\theta\right)$ denotes the prediction result generated by the CDL framework under parameter $\theta$ and weight $\omega$. The core optimization task involves dynamically updating $\left(\omega,\theta\right)$ in high-dimensional space through CLPO to minimize the objective function $J$. Regarding convergence criteria, CLPO employs a dual-criterion approach: first, reaching the maximum iteration count; second, early termination when the fitness decline falls below a threshold for consecutive generations, ensuring computational efficiency.

**The relationship between optimization iteration and prediction performance improvement:**

To validate the normativity and effectiveness of CLPO, Table 2 presents its comparison results with various benchmark methods in terms of convergence and under different load scenarios.

**Table 2. Comparison of CLPO with baseline methods in convergence and load scenarios**

| Method | Convergence Iterations (UK/FR) | Peak Load Accuracy (%) | Seasonal Stability (%) | RMSE in Extreme Weather (kW) | *p*-value | Reference |
| --- | --- | --- | --- | --- | --- | --- |
| CDL-PO | ~350 / ~370 | 86.2 | 82.5 | 5.72 | <0.01 | [37] |
| CDL-CLPO (ours) | 200–250 / 210–240 | 92.4 | 89.7 | 4.95 | / | / |
| Transformer-DGNN | ~280 / ~290 | 90.1 | 87.1 | 5.38 | 0.04 | [38] |
| EEMD-Attention | ~310 / ~320 | 88.3 | 84.9 | 5.61 | <0.01 | [39] |
| VMD-ResNet | ~400 / ~410 | 85.7 | 81.6 | 6.73 | <0.001 | [40] |

Comment 4: The weighted reconstruction mechanism for combining multiple model outputs lacks principled design criteria and theoretical analysis of how different models contribute to the final prediction under varying load conditions. The authors describe using dynamic weight adjustment through equation (7) but do not provide analysis of how weights are learned during training, how the system handles conflicting predictions from different models, or how the weighting strategy adapts to different load patterns such as peak demand periods, seasonal variations, or emergency scenarios that are critical in power system operations. Pl follow the study efficient deepfake detection via layer-frozen assisted dual attention network for consumer imaging devices.

Reply: Thank you for your comments. There is a lack of fundamental design principles for weighted reconstruction mechanisms that integrate multiple model outputs, as well as theoretical analysis of how different models contribute to the final prediction under varying load conditions. Additionally, the analysis of learning weights in Equation (7) has been supplemented to clarify how the system handles conflicting predictions from different models and how the weighting strategy adapts to distinct load patterns—such as peak demand periods, seasonal variations, or emergency situations. The specific modifications are as follows:

**There is no principled design criterion for the weighted reconstruction mechanism of multiple model outputs:**

To ensure the process adheres to fundamental principles, WR is designed around two core guidelines: “multi-scale complementarity + risk minimization.” On one hand, it requires the weight vector to remain within the probability simplex, guaranteeing the sum of contributions from the three submodels always equals 1. On the other hand, it decomposes the risk function into a prediction error term and a stability penalty term, achieving a theoretically Pareto-optimal equilibrium.

**Theoretical analysis of the contribution of different models to the final prediction under different load conditions:**

TCN weight increases during short-term spikes and abrupt changes, LSTM dominates in seasonal trends or long-term forecasting, while BiLSTM provides bidirectional correction during intraday double peaks and periodic alternation phases.

**Learning weight analysis:**

Furthermore, WR's weight optimization is typically determined by minimizing prediction error—achieved through weight allocation via an optimization objective function, as shown in Equation (8).

$\Gamma(w)=\sum_{t=1}^{T} \left(y_{t}-\sum_{i=1}^{N} \omega_{i}f_{i}(x_{t})\right)^{2}+\lambda\sum_{i=1}^{N} \omega_{i}^{2}$ (8)

In equation (8), $\Gamma(w)$ is the weighted error loss function. $y_{t}$ is the actual power load value. $T$ is the time step. $\lambda$ is a regularization term used to prevent overfitting. The regularization parameter not only prevents overfitting but also directly determines the system's ability to maintain prediction stability under varying load dynamics. Its mathematical relationship can be expressed as follows: when $\lambda$ is large, the regularization term $\lambda\sum_{i=1}^{N} \omega_{i}^{2}$ suppresses extreme deviations in the weight distribution, promoting more balanced contributions from multiple submodels. Consequently, during periods of intense load fluctuations, the upper bound of prediction variance is suppressed, resulting in more stable overall outputs. When $\lambda$ is small, the model relies more heavily on a single submodel. While this may improve local accuracy, stability decreases. Therefore, the magnitude of $\lambda$ positively correlates with the system's predictive stability under complex load conditions. Selecting an appropriate value for $\lambda$ achieves a balance between prediction accuracy and stability.

**The prediction of mutual conflict between different models shows that:**

To address potential conflicting predictions among TCN, LSTM, and BiLSTM during load fluctuations—such as one model emphasizing short-term volatility while another prioritizes long-term trends, leading to inconsistent prediction directions or magnitudes—WR dynamically optimizes the weight vector using a regularized least-squares method. During optimization, if outputs from different submodels diverge significantly, the algorithm automatically reduces the weight of the more volatile submodel. This enhances the overall smoothness and stability of the prediction results.

**Emergency modes for different load patterns:**

Furthermore, WR dynamically adjusts the contribution of each submodel by integrating historical residuals with real-time operational characteristics (such as power fluctuation rate and meteorological abruptness index), enabling quantification of the actual roles of different models during peak loads, seasonal variations, and unexpected events:

Comment 5: The Error Correction Model integration appears disconnected from the main deep learning framework and lacks detailed analysis of how long-term equilibrium relationships are established and maintained during dynamic load forecasting. The authors mention using ECM for joint error correction but do not provide mathematical formulation of how short-term prediction errors are corrected using long-term dependencies, how the error correction mechanism interacts with the neural network training process, or how this approach handles non-stationary load patterns that violate traditional error correction assumptions. Please follow the work bilateral feature fusion with hexagonal attention for robust saliency detection under uncertain environments.

Reply: Thank you for your comments. The mechanism for incorporating long-run equilibrium terms into recursive transmission has been described, elucidating how short-run forecast errors participate in corrections via long-run equilibrium relationships. Methods for addressing non-stationary load patterns that violate traditional error correction assumptions have been modified as follows:

**The mechanism of recursively introducing long-run equilibrium terms is described as follows:**

It is important to emphasize that the introduction of the Error Correction Model (ECM) does not operate in isolation from the deep learning framework, but rather forms a joint modeling approach with the BiLSTM output layer. Let the short-term prediction error be defined as shown in Equation (17).

 (17)

In Equation (17), $y\left(t\right)$ represents the actual load; represents the output of the deep network at time $t$. Based on this, ECM introduces the long-term equilibrium relationship as shown in Equation (18).

$\Delta y\left(t\right)\text{ }=\text{ }a\cdot e\left(t-1\right)\text{ }+\text{ }\beta\cdot Z\left(t\right)\text{ }+\text{ }\varepsilon\left(t\right)$ (18)

In Equation (18), $\Delta y\left(t\right)$ denotes the incrementally forecasted value corrected by the ECM, $a$ represents the coefficient of the error lag term, $Z\left(t\right)$ signifies the long-term equilibrium constraint variable (such as trend terms or seasonal components), and is the disturbance **The way in which short-term prediction errors are corrected by relying on long-term equilibrium relationships:**

In practical computations, the short-term forecast residuals in Equation (17) are recursively fed into the long-term equilibrium term of Equation (18) through gating. This mechanism enables historical residuals to exert a persistent influence on subsequent time steps' forecasts, which are gradually offset by long-term trends after multiple iterations. Consequently, error recursive correction driven by long-term dependencies is achieved. To ensure synergy with neural network training, ECM and BiLSTM share a unified loss function: during backpropagation, the correction term generated by ECM is directly incorporated into the gradient calculation of the total loss. This enables the network to simultaneously consider short-term fitting errors and long-term equilibrium constraints when updating parameters, thereby achieving end-to-end joint optimization.

**Response to non-stationary load patterns that violate conventional error correction assumptions:**

For instance, in extreme scenarios such as high penetration of renewable energy or sudden power outages, the model automatically increases the weight of long-term equilibrium terms to suppress abnormal fluctuations. During holiday periods with sudden load surges, it reduces long-term constraints to enhance short-term responsiveness, thereby ensuring the robustness and flexibility of the forecast.

Comment 6: The practical applicability for real-world power system deployment remains inadequately demonstrated despite claims about efficiency and reliability in complex load environments. The authors do not provide analysis of computational requirements for real-time forecasting, scalability to large-scale power grids, or robustness to data quality issues such as missing measurements, sensor failures, or communication delays that are common in practical power system monitoring. The system’s performance under extreme weather conditions, equipment outages, or other operational disruptions that significantly affect load patterns is not evaluated. Pl follow attention enhanced machine instinctive vision with human-inspired saliency detection.

Reply: Thank you for your comments. The original manuscript has validated the robustness results of different new energy sources after grid connection through Figure 11; verified the prediction capability of the testing model during different time periods—such as morning peak, evening peak, and nighttime—through Figure 12, confirming its stability; and demonstrated the model's performance under extreme and normal weather conditions via Table 4 (formerly Table 3). Therefore, the above tests sufficiently support the arguments regarding system robustness and practicality presented in this paper. This revision focuses solely on optimizing the description of these experimental results to enhance clarity and readability. Take the revisions of Figure 11 as an example, as follows:

As shown in Figure 11, within the range where the PV penetration rate increases from 6% to 36%, the proposed model maintains a prediction stability consistently above 85.6%, with significantly lower fluctuation than all comparison methods. This demonstrates that CDL-CLPO-ECM can continuously withstand load uncertainties caused by PV injection. When the penetration rate exceeds 24%, both VMD-ResNet and EEMD-Attention exhibit a noticeable decline in stability, indicating insufficient adaptability. The model maintains comparable advantages in wind energy scenarios: even with 36% wind penetration, its stability remains no less than 80.4% with minimal curve fluctuations. In contrast, Transformer-DGNN and VMD-ResNet exhibit rapid stability degradation beyond 21% penetration, struggling to handle the strong random fluctuations induced by wind energy. Overall, CLPO's global search combined with ECM's long-term equilibrium correction jointly reduces prediction variance in high-penetration scenarios, endowing the model with enhanced robustness.

Technical Questions:

1. How does the weighted reconstruction mechanism in equation (7) specifically handle the optimization of weights ωi when the three models (TCN, LSTM, BiLSTM) produce conflicting predictions during volatile load periods, and what is the mathematical relationship between the regularization parameter λ and the system’s ability to maintain prediction stability under varying load dynamics?

Reply: Thank you for your comments. The weighted reconstruction mechanism of weight optimization is established, and the stability relationship of regularization parameters is introduced. The revision is as follows:

**Weighted reconstruction mechanism:**

To address potential conflicting predictions among TCN, LSTM, and BiLSTM during load fluctuations—such as one model emphasizing short-term volatility while another prioritizes long-term trends, leading to inconsistent prediction directions or magnitudes—WR dynamically optimizes the weight vector using a regularized least-squares method. During optimization, if outputs from different submodels diverge significantly, the algorithm automatically reduces the weight of the more volatile submodel. This enhances the overall smoothness and stability of the prediction results.

**regularization parameter**

The regularization parameter not only prevents overfitting but also directly determines the system's ability to maintain prediction stability under varying load dynamics. Its mathematical relationship can be expressed as follows: when $\lambda$ is large, the regularization term $\lambda\sum_{i=1}^{N} \omega_{i}^{2}$ suppresses extreme deviations in the weight distribution, promoting more balanced contributions from multiple submodels. Consequently, during periods of intense load fluctuations, the upper bound of prediction variance is suppressed, resulting in more stable overall outputs. When $\lambda$ is small, the model relies more heavily on a single submodel. While this may improve local accuracy, stability decreases. Therefore, the magnitude of $\lambda$ positively correlates with the system's predictive stability under complex load conditions. Selecting an appropriate value for $\lambda$ achieves a balance between prediction accuracy and stability.

2. What is the exact algorithmic specification for the Continuous Learning-based Parrot Optimization process, particularly regarding how the parrot swarm intelligence mechanism adapts to the high-dimensional hyperparameter space of the combined deep learning models, and how does the optimization process ensure convergence while avoiding local minima in the complex loss landscape of power load forecasting?

Reply: Thank you for your comments. This paper clarifies the exact algorithm specification of the parrot optimization process based on continuous learning, and introduces the adaptation method of the high-dimensional hyperparameter space of the parrot swarm intelligent mechanism, as well as the operation to avoid local minima. The modifications are as follows:

**Exact algorithm specification for the parrot optimization process based on continuous learning:**

The optimization of CLPO can be summarized in four steps: ① Initialization via chaotic search generates a uniform initial population in the high-dimensional hyperparameter space encompassing prediction weights, learning rate, error correction coefficient, etc., with fitness calculated using objective function A (Equation 15); ② Dynamic classification of individuals into explorers and exploiters based on fitness, where explorers perform large-step jumps while exploiters fine-tune within the optimal neighborhood; ③ Continuous learning records historical optima, balancing global search and local convergence through decreasing inertia weights and velocity adjustment factors; ④ Iteration terminates when the upper limit is reached or the objective function's decline over B consecutive generations falls below threshold C, outputting optimal parameters.

**Adaptation of high dimensional hyperparameter space for parrot swarm intelligence mechanism:**

When coupled with combinatorial deep learning models, CLPO unifies the core hyperparameters of TCN, LSTM, BiLSTM, and ECM into a high-dimensional search vector. Each position update optimizes this vector holistically, enabling coordinated adjustment across multi-layer submodels.

**Avoid local minima:**

By enhancing the search scope through chaotic perturbation and combining a monotonically non-increasing objective function with dual-criterion convergence criteria, CLPO avoids local minima and ensures convergence in the complex non-convex loss landscape of power load forecasting.
