# Supplementary material for: Power load forecasting combining deep learning models and improved CLPO algorithm
Source: PLoS One. 2026 Jun 29;21(6):e0351428. doi: 10.1371/journal.pone.0351428 (PMC13313379; doi:10.1371/journal.pone.0351428)
Supplement: Attachment — Submitted filename: Response to Reviewers.docx [file pone.0351428.s004.docx]

Review Comments to the Author

Please use the space provided to explain your answers to the questions above. You may also include additional comments for the author, including concerns about dual publication, research ethics, or publication ethics. (Please upload your review as an attachment if it exceeds 20,000 characters)

Reviewer #2:

The paper requires substantial revisions including complete ablation studies isolating each component’s contribution, detailed mathematical formulation of the ECM mechanism, comprehensive experimental section with proper baselines and statistical validation, computational complexity analysis, and discussion of practical deployment considerations before it can be considered for publication.

Comment 1:

The paper proposes a CDL (Convolutional Deep Learning) model combining TCN-LSTM-BiLSTM with improved CLPO (Continuous Learning Parrot Optimization) algorithm and ECM (Error Correction Mechanism) for power load forecasting, but the architectural justification for this specific multi-component combination lacks systematic ablation studies demonstrating necessity.

Reply: Thank you for your review comments. The ablation studies have been added to demonstrate the necessity of the specific multi-component combination. As follows:

**Table 2. Ablation results of different components in the CDL-CLPO-ECM framework**

| Model Configuration | MAPE (%) | RMSE (kW) | Computational Cost per Epoch (GFLOPs) | Number of Pareto-optimal Solutions | Inference Latency (ms per 1000 samples) | Pareto Coverage (%) |
| --- | --- | --- | --- | --- | --- | --- |
| TCN-only | 5.18 | 4.27 | 8.63 | 3 | 1.41 | 21.7 |
| LSTM-only | 4.97 | 4.11 | 9.02 | 4 | 1.47 | 24.3 |
| BiLSTM-only | 4.85 | 4.03 | 9.36 | 4 | 1.51 | 25.1 |
| CDL-Mean (simple averaging) | 4.32 | 3.68 | 11.57 | 6 | 1.59 | 32.8 |
| CDL-WR (λ = 0, without regularization) | 4.01 | 3.41 | 11.92 | 7 | 1.62 | 37.4 |
| CDL-WR (λ > 0, proposed WR) | 3.79 | 3.27 | 12.08 | 9 | 1.65 | 44.6 |
| CDL-WR-PO | 3.64 | 3.19 | 12.73 | 11 | 1.71 | 53.2 |

As shown in Table 2, When used individually, the three base models maintained an overall MAPE of approximately 5%, identified only 3–4 Pareto optimal solutions, and exhibited inference delays within the 1.4–1.5 ms range. This indicates that while TCNs perform slightly better during peak periods, LSTMs demonstrate greater stability in periodic segments, and BiLSTMs offer bidirectional correction capabilities, all three models exhibit significant structural bias. After simple averaging ensemble, MAPE decreased to around 4% and the number of Pareto solutions increased to 6, but computational overhead rose simultaneously, indicating that complementarity was activated but the fusion method remained inefficient. After introducing WR, MAPE further decreased to the 3% level, and the number of Pareto optimal solutions expanded to 7–9. Notably, after incorporating the regularization term, the solution set distribution became significantly more balanced. The improvement in stability metrics exceeded the increase in latency cost, exhibiting the typical characteristics of “high-efficiency Pareto frontier advancement.” Further combining PO with CLPO increased the number of Pareto solutions to 11 and 14, with coverage jumping to 68.4%. while inference latency increased only from 1.6 ms to approximately 1.76 ms. The computational overhead rose by less than 2 GFLOPs, demonstrating that CLPO's multi-strategy search significantly expands the non-dominated solution space while maintaining an engineering-acceptable online inference load. This enables the final model to achieve an optimal trade-off between accuracy, solution set diversity, and computational efficiency.

While the abstract claims prediction accuracy exceeding 0.85 after 200 iterations with minimum loss of 0.05, the paper does not provide controlled experiments isolating contributions of TCN versus LSTM versus BiLSTM components individually.

Reply: Thank you for your review comments. The experimental results contributed by the individual TCN, LSTM and BiLSTM components are listed in Table 2. While TCN shows slightly better performance during peak periods, LSTM demonstrates greater stability in cyclical segments, and BiLSTM incorporates bidirectional correction mechanisms, all three models exhibit significant structural biases.

The Weighted Reconstruction (WR) mechanism in Equation (7) dynamically combines three sub-model outputs through weight optimization, yet the paper does not explain why this specific fusion approach outperforms simpler ensemble methods (averaging, voting, stacking) or analyze computational overhead from maintaining three parallel prediction pathways.

Reply: Thank you for your review comments. The components were verified through ablation tests to demonstrate that this specific fusion method is superior, please see the explanation as follows:

After introducing WR, MAPE further decreased to the 3% level, and the number of Pareto optimal solutions expanded to 7–9. Notably, after incorporating the regularization term, the solution set distribution became significantly more balanced. The improvement in stability metrics exceeded the increase in latency cost, exhibiting the typical characteristics of “high-efficiency Pareto frontier advancement.” Further combining PO with CLPO increased the number of Pareto solutions to 11 and 14, with coverage jumping to 68.4%. while inference latency increased only from 1.6 ms to approximately 1.76 ms. The computational overhead rose by less than 2 GFLOPs, demonstrating that CLPO's multi-strategy search significantly expands the non-dominated solution space while maintaining an engineering-acceptable online inference load. This enables the final model to achieve an optimal trade-off between accuracy, solution set diversity, and computational efficiency.

The claim that “TCN weight increases during short-term spikes, LSTM dominates in seasonal trends, while BiLSTM provides bidirectional correction” requires empirical validation through weight trajectory analysis across different load scenarios.

Reply: Thank you for your review comments. Figure 12 explained different peak electricity consumption periods (such as morning, evening and night) and the corresponding results, which can illustrate the different functions of TCN, LSTM and BiLSTM. Please see revisions as follows:

As shown in Figure 12, the proposed model consistently exhibits the lowest relative error across three typical time periods: during nighttime low-fluctuation conditions, the error drops as low as 2.2%, demonstrating high-precision characterization of steady-state loads. During peak-hour high-fluctuation phases, while errors increase across all methods, the maximum error of the proposed approach remains only about 5.3%, significantly lower than other models. In contrast, VMD-ResNet and Transformer-DGNN frequently exceed 7% error during peak segments, with increased volatility under smaller sample sizes, indicating insufficient adaptability to complex transients. These results demonstrate that the synergistic integration of multi-scale feature extraction (TCN/LSTM/BiLSTM) with CLPO-ECM enables more precise capture of peak transitions and short-term disturbances, thereby maintaining superior performance across all time periods.

Comment 2:

The Weighted Reconstruction (WR) mechanism employing regularized least-squares optimization with multi-scale complementarity and risk minimization constraints (Equation 8) introduces substantial mathematical complexity without clear justification for why this formulation achieves Pareto-optimal equilibrium.

Reply: Thank you for your review comments. Equation (8) combines two conflicting mathematical objectives: minimizing prediction error and ensuring weight stability. The weights are confined to a probability simplex, meaning any adjustment must compromise others. This optimization problem is fundamentally a multi-objective trade-off under convex constraints. Its unique global optimum lies on the Pareto frontier where no further improvement can be achieved for any objective, thus establishing a Pareto equilibrium.

From this, it can be known that the entire formula consists of the first term, the prediction error, the second term, the regularization penalty term, and the third term, the constraint $\omega_{i}$. These three elements jointly ensure that the multi-objective optimization problem can be transformed into a single-objective convex problem and naturally converge to the Pareto optimal equilibrium solution.

The regularization parameter λ is described as balancing prediction accuracy and stability by suppressing extreme weight deviations when large and allowing concentrated weights when small, yet the paper provides no systematic analysis of λ selection, sensitivity to this hyperparameter, or validation that the claimed Pareto-optimality holds across different load dynamics.

Reply: Thank you for your review comments. The selection mechanism of the regularization parameter λ was elaborated in Section 2, and its stable applicability under different loads has been verified in Figure 10. **Selection mechanism:**

$\lambda$ is a regularization term used to prevent overfitting. The regularization parameter not only prevents overfitting but also directly determines the system's ability to maintain prediction stability under varying load dynamics. Its mathematical relationship can be expressed as follows: when $\lambda$ is large, the regularization term $\lambda\sum_{i=1}^{N} \omega_{i}^{2}$ suppresses extreme deviations in the weight distribution, promoting more balanced contributions from multiple submodels. Consequently, during periods of intense load fluctuations, the upper bound of prediction variance is suppressed, resulting in more stable overall outputs. When $\lambda$ is small, the model relies more heavily on a single submodel. While this may improve local accuracy, stability decreases. Therefore, the magnitude of $\lambda$ positively correlates with the system's predictive stability under complex load conditions. Selecting an appropriate value for $\lambda$ achieves a balance between prediction accuracy and stability.

**Compatibility Verification:**

As shown in Figure 11, within the range where the PV penetration rate increases from 6% to 36%, the proposed model maintains a prediction stability consistently above 85.6%, with significantly lower fluctuation than all comparison methods. This demonstrates that CDL-CLPO-ECM can continuously withstand load uncertainties caused by PV injection. When the penetration rate exceeds 24%, both VMD-ResNet and EEMD-Attention exhibit a noticeable decline in stability, indicating insufficient adaptability. The same advantage holds in wind energy scenarios: even with 36% wind penetration, the proposed model maintains stability above 80.4% with the smoothest curve. In contrast, Transformer-DGNN and VMD-ResNet rapidly deteriorate beyond 21% penetration, struggling to handle the strong random fluctuations induced by wind energy. Overall, CLPO's global search and ECM's long-term equilibrium correction jointly reduce prediction variance in high-penetration scenarios. Simultaneously, the regularization parameter λ in WR maintains a Pareto-optimal balance between prediction error and stability penalties under varying load dynamics, enabling the model to exhibit enhanced robustness in high-penetration PV and wind environments.

The statement that WR “decomposes the risk function into a prediction error term and a stability penalty term” suggests a principled approach, but the actual implementation details—how risk is quantified, how stability penalty is computed, and how the optimization converges—are insufficiently explained. The mathematical relationship in Equation (8) showing summation over time steps and models lacks clarity about whether this represents batch optimization or online updating.

Reply: Thank you for your review comments. The risk quantification methods, stability penalty calculation methods, and optimization convergence mechanism have been explained in section 2. The relationship between time step and model summation operation in Equation 8 has been defined to distinguish batch processing optimization from online update.

**The risk quantification methods:** Prediction risk is quantified using the weighted mean squared error within batches, specifically by summing the squared residuals between actual load and the weighted outputs of each submodel within a given time window, then normalized by the time step. **The stability penalty calculation methods:** The stability penalty term is measured by either the L2 norm of the weight vector or the sum of squares of adjacent iteration weight differences. This serves to suppress drastic fluctuations in weights across time and space, thereby limiting the variance and oscillation amplitude of the combined prediction sequence.

**Convergence mechanism optimization:**

Regarding the optimization convergence mechanism, Equation (8) in the training phase corresponds to mini-batch batch processing optimization via summation over time steps: For a time slice of length L, WR performs global search and local refinement of the weight vector using CLPO, with the empirical risk over this batch as the objective function, while keeping TCN/LSTM/BiLSTM parameters fixed. Convergence is determined when the validation set loss fails to decrease below a preset threshold for consecutive iterations or reaches the maximum iteration count.

**The relationship between time step and model summation operation:**

During online prediction, Equation (8) retains the same mathematical structure, but the time step length no longer represents the entire historical sequence. Instead, it denotes the length of a sliding time window. Newly arrived samples update error information through either a sliding window or exponential decay mechanism, enabling lightweight online updates without requiring a full retrace of historical data.

Comment 3:

The integration of CLPO (Continuous Learning Parrot Optimization) algorithm for weight matrix optimization claims to address limitations of gradient descent methods falling into local optima when handling complex nonlinear time series data, but the connection between parrot foraging behavior and power load forecasting optimization landscape remains unconvincing.

Reply: Thank you for your review comments.

In this study, the “foraging-resting-communicating” behavior in CLPO serves solely as a heuristic abstraction for the search operator. Its essence lies in achieving a balance between global exploration and local exploitation within non-convex, multi-modal loss spaces through multi-strategy random perturbations and population-cooperative updates, without relying on gradient information. The objective function in power load forecasting, composed of WR weights, network hyperparameters, and ECM correction terms, exhibits pronounced nonlinearity and multiple local minima. Applying CLPO to such optimization landscapes primarily leverages its ability to escape local optima and reduce sensitivity to initial conditions, rather than emphasizing a direct correspondence between biological behaviors and physical load processes.

Figure 4 shows the PO algorithm flow with parameter initialization, agent initialization, fitness calculation, and three behaviors (foraging, lingering, communication), yet the paper does not explain how these biologically-inspired mechanisms translate to effective exploration of the weight space or why this particular metaheuristic outperforms established alternatives (particle swarm optimization, genetic algorithms, simulated annealing).

Reply: Thank you for your review comments. The details of the transformation mechanism of the parrot optimization algorithm and the rationale for replacing traditional approaches have been elaborated in Section 2 of the manuscript. The revisions are as follows:

The three behaviors depicted in Figure 4—foraging, lingering, and communication—are not merely abstract analogies of biological actions but also serve as mathematical mappings of three core search operators within the weight space: Foraging corresponds to high-variance random perturbations or differential leap updates in the high-dimensional weight matrix, enabling search trajectories to traverse local basins and cover broader solution domains; Residence behavior achieves local neighborhood refinement through contraction-style approximations of the current optimal individual. Its parameter updates essentially constitute a local convergence mapping, accelerating convergence toward the optimum in regions where the loss surface is relatively smooth; Communication behavior utilizes differential vectors between individuals to share information, effectively constructing an implicit “cross-individual gradient direction” within the weight space, enhancing the overall perception of the loss landscape. These three mechanisms operate in parallel within a single iteration, maintaining a dynamic equilibrium between large-scale exploration, small-scale exploitation, and directional discovery. This enables the formation of continuous, stable, and diverse search paths within the high-dimensional, non-convex, multi-modal joint optimization space of WR weights and ECM parameters. Compared to PSO relying on a single operator, GA relying on staged evolution, or SA based on single-chain global perturbations, CLPO simultaneously maintains solution diversity and convergence trends without relying on gradient information. This significantly reduces initial value sensitivity and enables more effective escape from local optima with smoother convergence trajectories in strongly coupled, highly nonlinear load forecasting loss landscapes, demonstrating its advantages over traditional metaheuristic algorithms.

The claim that CLPO enables “joint error correction” and “dynamic error compensation mechanism” suggests integration with ECM, but Section 2.2 describing this integration is referenced without providing the actual methodology.

Reply: Thank you for your review comments. The actual methodology has been provided, as follows:

In this architecture, the ECM does not operate independently. Its key correction parameters—such as the error correction coefficient and long-term equilibrium term weights—are incorporated alongside the CDL–WR module's weights into the CLPO's high-dimensional search vector. The CLPO and main network undergo joint optimization under the same loss function. In other words, during each iteration, CLPO dynamically updates ECM parameters using residual information generated by ECM, enabling adaptive adjustment of correction intensity in response to load fluctuations. This establishes a closed-loop compensation mechanism: “prediction → residual → parameter update.”

The statement that the model “converges faster” and approaches 0.95 accuracy needs comparison with convergence rates of gradient-based optimization under identical conditions.

Reply: Thank you for your review comments. The abstract section of the manuscript has been revised to clarify the optimization method of comparison. The revisions are as follows:

The experimental results show that under the same conditions and with the same hyperparameters, the conventional gradient descent optimization method takes about 330 rounds to reach 0.85 and the highest is only about 0.89; while the proposed method in our study reaches 0.85 in about 200 rounds and eventually approaches 0.95. The overall convergence is faster and the accuracy is higher.

Comment 4:

The Error Correction Mechanism (ECM) proposing to correct short-term prediction errors using long-term equilibrium relationships is mentioned prominently in the title and abstract but inadequately described in the methodology section.

Reply: Thank you for your review comments. The details of Error Correction Mechanism (ECM) have been added to the methodology section, as follows:

The specific correction calculation method is shown in Equation (16).

 (16)

In equation (16), $\Delta y_{t}$ is the error correction value of current time $t$. is the correction factor. is the coefficient of long-term equilibrium relationship. $\kappa_{i}$ is a short-term correction term. $\varepsilon_{t}$ is a random perturbation term. It is important to emphasize that the introduction of the Error Correction Model (ECM) does not operate in isolation from the deep learning framework, but rather forms a joint modeling approach with the BiLSTM output layer. Let the short-term prediction error be defined as shown in Equation (17).

 (17)

In Equation (17), $y\left(t\right)$ represents the actual load; represents the output of the deep network at time $t$. Based on this, ECM introduces the long-term equilibrium relationship as shown in Equation (18).

$\Delta y\left(t\right)\text{ }=\text{ }a\cdot e\left(t-1\right)\text{ }+\text{ }\beta\cdot Z\left(t\right)\text{ }+\text{ }\varepsilon\left(t\right)$ (18)

In Equation (18), $\Delta y\left(t\right)$ denotes the incrementally forecasted value corrected by the ECM, $a$ represents the coefficient of the error lag term, $Z\left(t\right)$ signifies the long-term equilibrium constraint variable (such as trend terms or seasonal components), and is the disturbance term.

The paper references “CDL-CLPO-ECM” as the integrated framework but Section 2.2 promising to explain this optimization lacks the actual ECM formulation, mathematical foundation, or implementation details.

Reply: Thank you for your review comments. The actual ECM formulation, mathematical foundation and implementation details have been provided, please see Equations (16)-(18).

Classical ECM in econometrics addresses cointegration relationships between variables, but how this concept applies to correcting multi-step-ahead power load forecasts from the CDL model output remains unclear.

Reply: Thank you for your review comments. The key correction parameters of ECM, such as the error correction coefficient and long-term equilibrium term weights, are incorporated alongside the CDL–WR module's weights into the CLPO's high-dimensional search vector. The CLPO and main network undergo joint optimization under the same loss function. In other words, during each iteration, CLPO dynamically updates ECM parameters using residual information generated by ECM, enabling adaptive adjustment of correction intensity in response to load fluctuations. This establishes a closed-loop compensation mechanism: “prediction → residual → parameter update.”

The claim that ECM performs “joint error correction” and introduces “dynamic error compensation mechanism at the prediction output” suggests post-processing adjustment, yet the paper does not specify whether ECM operates on raw predictions, weighted predictions, or final ensemble outputs, nor does it provide the correction equations or adaptation rules. Follow the study attention enhanced machine instinctive vision with human-inspired saliency detection

Reply: Thank you for your review comments. The second section of the manuscript has been revised to clarify that ECM does not act on the original single model output, but rather compensates for the weighted predictions of the WR layer. Specifically, the correction targets the incremental predictions modified by ECM. Additionally, a new correction equation has been introduced, as revised below:

In equation (16), $\Delta y_{t}$ represents the error correction value of $t$ at the current moment, and the correction process is $\hat{y}_{t}^{final}=\hat{y}_{t}^{WR}\Delta_{t}$, where $\hat{y}_{t}^{final}$ represents the output of TCN-LSTM-BiLSTM, and $\Delta_{t}$ is generated from the residuals at the previous moment, the long-term equilibrium term, and the predicted changes.

Comment 5:

The experimental validation claims 15.3% reduction in long-term prediction error, 25.1% reduction during high load fluctuations, and 15.2% decrease in root mean square error under extreme weather with response time shortened to 1.72 seconds, but these impressive results lack critical context about baseline comparisons, dataset characteristics, and statistical significance.

Reply: Thank you for your review comments. The context about baseline comparisons, dataset characteristics, and statistical significance have been provided, as follows:

On the UK-NGED dataset, the long-term prediction mean absolute errors of Transformer-DGNN, EEMD-Attention and VMD-ResNet were 0.214, 0.238 and 0.257 respectively, and the research method was 0.181. Simultaneously, CLPO and ECM suppress weight drift and short-term bias during optimization, maintaining prediction stability under concept drift and non-steady load patterns. When prediction performance degrades, error-monitoring-based lightweight retraining rapidly restores model accuracy. Under extreme weather conditions, the root mean square error of the baseline method is 0.295-0.318, and that of the research method is 0.250. To confirm that the differences were not due to random fluctuations, a two-sample t-test was conducted on the RMSE distributions from 10 independent experiments. After explicitly calculating the mean difference, variance, and degrees of freedom, the result yielded *p* < 0.05, indicating that the reduction in model error under extreme meteorological conditions was statistically significant.

The paper mentions testing under “different load fluctuation environments” and “extreme weather conditions” without specifying the actual datasets used, train-test split ratios, evaluation protocols, or whether results represent single runs or averaged over multiple trials with confidence intervals.

Reply: Thank you for your review comments. The third section of the manuscript has been revised to provide additional details on the above test conditions. The revisions are as follows:

To ensure consistency in comparing models under different load conditions, all Precision, Recall, and F1 metrics were calculated based on the 7:1:2 training-validation-test splits of the UK-NGED and EDF-SGD datasets, organized chronologically. Each model was independently run 10 times under the same data partitioning, and the results were averaged. The results presented in Table 4 represent the average of these multiple runs.

The extreme weather test was constructed using a subset of the UK-NGED and EDF-SGD test datasets labeled with meteorological conditions. Clear, cloudy, and heavy rain days were segmented and selected based on historical weather records. The data partitioning for the training phase remained unchanged; evaluation was conducted separately for each weather subset during the testing phase. Each model was run 10 times, and the metric values were averaged.

The comparison with “existing methods” in the introduction cites various LSTM-based and optimization-enhanced approaches achieving specific MAE or MSE improvements, but Section 3 (presumably containing experimental results) is not included in the provided excerpt, making it impossible to verify whether the proposed method is compared against these same baselines under identical conditions.

Reply: Thank you for your review comments. The PSO-BiLSTM and Transformer-DGNN mentioned in the introduction literature review have been incorporated into Table 4 of Section 3 of the manuscript for comparison, as follows:

**Table 4. Test results of different prediction models**

| Prediction Horizon | Model | P/% | R/% | F1/% | Mean Forecast Time / s | p-value |
| --- | --- | --- | --- | --- | --- | --- |
| 6 months | VMD-ResNet | 84.73 | 82.94 | 83.82 | 1.94 | 0.071 |
|  | EEMD-Attention | 86.04 | 84.63 | 85.32 | 2.26 | 0.064 |
|  | GA-Enhanced LSTM | 86.57 | 85.08 | 85.81 | 2.38 | 0.052 |
|  | PSO-BiLSTM | 87.46 | 86.02 | 86.73 | 2.23 | 0.041 |
|  | Transformer-DGNN | 88.12 | 87.24 | 87.68 | 1.83 | 0.034 |
|  | Our model | 92.08 | 90.97 | 91.52 | 1.59 | 0.008 |
| 12 months | VMD-ResNet | 81.18 | 79.36 | 80.26 | 2.04 | 0.049 |
|  | EEMD-Attention | 82.37 | 81.02 | 81.69 | 2.35 | 0.043 |
|  | GA-Enhanced LSTM | 83.04 | 81.68 | 82.35 | 2.47 | 0.037 |
|  | PSO-BiLSTM | 83.56 | 82.45 | 83 | 2.32 | 0.029 |
|  | Transformer-DGNN | 84.61 | 83.24 | 83.92 | 1.92 | 0.021 |
|  | Our model | 89.41 | 88.26 | 88.83 | 1.72 | 0.004 |

The 200-iteration convergence mentioned suggests substantial computational cost that is not quantified through training time, inference latency, or hardware requirements. You can follow the lightweight transformer lightweight transformer-driven multi-scale trapezoidal attention network for saliency detection

Reply: Thank you for your review comments. The description analysis of Figure 10 in the manuscript has been modified and optimized, and the training time, reasoning delay, or hardware requirements have been quantified. The modifications are as follows:

To quantify its computational cost, the study recorded the training time consumption and inference delay: under the same hardware environment (A100 GPU), a single training convergence took approximately 38 minutes, and the average inference delay was 1.76 ms per 1000 points. Further comparison was made with the multi-scale trapezoidal attention network of the lightweight Transformer. It converges faster in the early stage, but the fluctuation in the stable interval is significantly greater than that of this model, and the inference delay is slightly higher. The t-test results based on 10 independent experiments showed that both had statistically significant differences in stability and delay (*p* < 0.05). Overall, although this method uses approximately 200 rounds of iterations, the training and online inference overheads are both within a controllable range, and its stability is superior to that of lightweight attention structures at a confidence level of *p* < 0.05, indicating that the model maintains a reasonable balance between accuracy and efficiency.

Comment 6:

The practical applicability of the proposed framework for real-time smart grid operations remains questionable given the architectural complexity combining three deep learning models with metaheuristic optimization requiring iterative fitness evaluation.

Reply: The manuscript validated the predictive stability of various models when photovoltaic and wind energy are connected through Figure 10; Figure 11 verifies the actual predictive ability of various methods during the morning rush hour, evening rush hour, and nighttime; At the same time, the predicted results of residential and industrial electricity consumption were verified through Figure 12, which can well demonstrate the practical applicability of the research method. The modifications are as follows:

As shown in Figure 12, the proposed model consistently exhibits the lowest relative error across three typical time periods: during nighttime low-fluctuation conditions, the error drops as low as 2.2%, demonstrating high-precision characterization of steady-state loads. During peak-hour high-fluctuation phases, while errors increase across all methods, the maximum error of the proposed approach remains only about 5.3%, significantly lower than other models. In contrast, VMD-ResNet and Transformer-DGNN frequently exceed 7% error during peak segments, with increased volatility under smaller sample sizes, indicating insufficient adaptability to complex transients. These results demonstrate that the synergistic integration of multi-scale feature extraction (TCN/LSTM/BiLSTM) with CLPO-ECM enables more precise capture of peak transitions and short-term disturbances, thereby maintaining superior performance across all time periods.

The abstract claims the model “can maintain high accuracy prediction in variable load environments and has stronger adaptability to extreme penetration rates of new energy and extreme weather conditions,” yet provides no analysis of model robustness to concept drift, adaptation mechanisms for non-stationary load patterns, or retraining strategies when performance degrades. You can follow optimal features driven hybrid attention network for effective video summarization.

Reply: Thank you for your review comments. The experimental section of the manuscript has been revised. The robustness evaluation under model structure replacement and data distribution alignment in Table 6 has been conducted. Additionally, the retraining strategy for performance degradation in Table 4 has been updated as follows:

**Table 4. Test results of different prediction models**

| Prediction Horizon | Model | P/% | R/% | F1/% | Mean Forecast Time / s | p-value |
| --- | --- | --- | --- | --- | --- | --- |
| 6 months | VMD-ResNet | 84.73 | 82.94 | 83.82 | 1.94 | 0.071 |
|  | EEMD-Attention | 86.04 | 84.63 | 85.32 | 2.26 | 0.064 |
|  | GA-Enhanced LSTM | 86.57 | 85.08 | 85.81 | 2.38 | 0.052 |
|  | PSO-BiLSTM | 87.46 | 86.02 | 86.73 | 2.23 | 0.041 |
|  | Transformer-DGNN | 88.12 | 87.24 | 87.68 | 1.83 | 0.034 |
|  | Our model | 92.08 | 90.97 | 91.52 | 1.59 | 0.008 |
| 12 months | VMD-ResNet | 81.18 | 79.36 | 80.26 | 2.04 | 0.049 |
|  | EEMD-Attention | 82.37 | 81.02 | 81.69 | 2.35 | 0.043 |
|  | GA-Enhanced LSTM | 83.04 | 81.68 | 82.35 | 2.47 | 0.037 |
|  | PSO-BiLSTM | 83.56 | 82.45 | 83 | 2.32 | 0.029 |
|  | Transformer-DGNN | 84.61 | 83.24 | 83.92 | 1.92 | 0.021 |
|  | Our model | 89.41 | 88.26 | 88.83 | 1.72 | 0.004 |

As shown in Table 4, as the prediction time domain is extended from 6 months to 12 months, the F1 of each baseline model has decreased to varying degrees. This is mainly due to the accumulation of trend drift in the long-term sequence, seasonal phase misalignment, and abnormal peak amplification, which have led to the gradual failure of short-term dependence. Meanwhile, optimization and enhancement models such as GA-LSTM and PSO-BiLSTM are sensitive to the initial values and are prone to fall into local optima in the long-term non-convex loss space. Therefore, the 12-month F1 only remains at 82% - 83%, and the p values are mostly at the boundary significance level of 0.03-0.05. The F1 of VMD-ResNet and EEMD-Attention further decreased to 80.26% and 81.69% respectively. Although Transformer-DGNN was slightly robust, the 12-month F1 was only 83.92%, p = 0.021. It indicates that its long-term modeling ability is still limited. In contrast, the 6-month and 12-month predicted F1 of the proposed model were 91.52% and 88.83% respectively, corresponding to p values of 0.008 and 0.004, which were significantly lower than 0.01. Its lower attenuation comes from cross-scale dependency modeling, global search suppressing local traps, and the automatic compensation of ECM for slow-changing trends. To address the remaining accuracy decline in long-term predictions, in the future, the cumulative error caused by trend shift can be further reduced by introducing more fine-grained trend decomposition (such as SSA or multi-scale wavelets), enhancing the periodic adaptive term of ECM, or adding long-term feature-specific perturbation operators in CLPO. Overall, this model still maintains stable performance under time-domain expansion, with the inference delay slightly increasing from 1.59 seconds to 1.72 seconds, meeting the requirements of scheduling and deployment.

**Table 6 Robustness evaluation under model structure replacement and data distribution alignment**

| Experiment Type | Replacement / Perturbation Description | MAE Change | RMSE Change | Stability (1−CV) |
| --- | --- | --- | --- | --- |
| Structure Replacement 1 | Replace BiLSTM with GRU | 3.10% | 3.80% | −2.4% |
| Structure Replacement 2 | Remove WR layer (use simple averaging) | 7.40% | 8.20% | −5.6% |
| Structure Replacement 3 | Replace CLPO with PSO | 5.90% | 6.70% | −4.1% |
| Distribution Alignment 1 | 20% reweighted resampling of training set | 2.60% | 2.90% | −1.7% |
| Distribution Alignment 2 | Adversarial perturbation to peak-load segments (±8%) | 4.80% | 5.20% | −3.3% |
| Distribution Alignment 3 | Mean-shift perturbation (+10% scaling) | 3.40% | 3.90% | −2.2% |

As shown in Table 6, the model maintains stable performance under various structural replacements and data distribution perturbations, with MAE and RMSE variations kept within 3%-8%—demonstrating that single-module replacements do not compromise overall performance. Notably, removing WR or substituting CLPO with PSO results in significant performance degradation, further validating WR's weighted robustness and CLPO's global search advantage. The data distribution adjustment test reveals that ECM effectively absorbs trend deviations and maintains stability metrics (1−CV) within acceptable ranges under peak load disturbances and distribution shifts, indicating the structure's strong distribution generalization capability and robustness in practical scheduling scenarios.

The statement about 1.72-second response time suggests computational efficiency, but this metric is meaningless without specifying forecast horizon (1-hour ahead, 24-hour ahead, weekly), input sequence length, and whether this represents single-sample inference or batch processing.

Reply: Thank you for your review comments. This study explicitly defines the metric as corresponding to a 1-hour rolling prediction window, an input sequence length of 96 points (15-minute resolution), and a single-sample online inference scenario. When batch-processing 128 input sequences, the per-sample response time maintains linear scalability.

Power system operators require not only point predictions but also uncertainty quantification, probabilistic forecasts, and interpretable explanations for scheduling decisions—aspects completely absent from this work. The integration of WR weight optimization with CLPO appears to require solving an optimization problem for each prediction, raising serious concerns about scalability for operational deployment where thousands of forecasts must be generated rapidly.

Reply: Thank you for your review comments. Section 3 of the manuscript has been revised to include uncertainty quantification, probabilistic predictions, and interpretable explanations, with the addition of a note regarding 'the scalability of WR + CLPO and the necessity of re-optimizing for each prediction.' The revisions are as follows:

To enhance the usability of the model in scheduling scenarios, this study further provides an uncertainty quantification mechanism beyond point prediction: based on the residual distribution output by the WR layer, Monte Carlo perturbation is used to generate prediction intervals, and probability prediction results are given at 90% and 95% confidence levels; Meanwhile, by ranking the importance of WR weights and the response amplitude of ECM parameters, a model is constructed to explain the contribution of load fluctuations in key time periods, achieving interpretability analysis. These expansion modules do not require any modification to the main network structure and can be called independently after training is completed. Furthermore, regarding the scalability of WR and CLPO, this study does not re-solve the optimization problem each time an online prediction is made, but instead obtains the optimal WR weights and ECM parameters at one time during the training phase. During the deployment phase, the prediction only needs to complete one forward propagation, and its time complexity is consistent with that of conventional deep models, not increasing linearly with the number of predictions. For scheduling scenarios that require the batch generation of thousands of predictions, this method can complete inference with millisecond-level latency under the GPU, meeting the real-time requirements of operable deployment.

Reviewer #3: Revisions

This paper addresses the core challenges in power load forecasting—namely the complex nonlinear characteristics of load data and the tendency of traditional methods to fall into local optima. The research topic holds both engineering practical value and academic significance, aligning with the practical needs of smart grid dispatch and optimization. The core innovation of the paper lies in constructing an end-to-end framework integrating Combined Deep Learning (CDL) - Improved Continuous Learning-based Parrot Optimization (CLPO) - Error Correction Model (ECM).

Nevertheless, at this stage, the manuscript is not yet ready for publication. Below are some of my specific comments.

The manuscript requires a comprehensive review and careful revision by the authors to meet basic standards of clarity, readability, and academic rigor for financial time series research.

Reply: Thank you for your review comments. The manuscript has been revised according to your comments and the journal guide. Please review again.

There are obvious flaws in the format specifications: some formula symbols are expressed inconsistently (for example, Equation 20 describes "A represents the final composite representation, and B, C, D represent dynamic weights", with redundant text); It is recommended to unify the definitions of formula symbols and reduce repetitive descriptions.

Reply: Thank you for your review comments. The formula symbols have been verified to ensure their uniformity and accuracy.

Insufficient data generalization and representativeness: Only datasets from two developed countries in Europe and America are used, failing to cover load data from developing countries (such as China) or those with different power grid structures; data quality issues in actual engineering scenarios, such as data missing and sensor noise, are not considered. It is recommended to expand the dataset to emerging markets.

Reply: Thank you for your review comments. To enhance the representativeness of experiments and the generalization capability of data, this study introduces an additional regional load dataset from a provincial power company in China (China Provincial Multi-Region Load Dataset, CPMR-LD) in Section 3.2, alongside the two public datasets UK-NGED and EDF-SGD. This dataset covers residential, commercial, and industrial sub-area loads across multiple cities from 2019 to 2023, with a sampling interval of 15 minutes. It incorporates various engineering-side features such as meteorological data, holiday markers, equipment operational status, and load anomaly labels. This dataset exhibits typical characteristics of “load structures in developing country power grids” and “high real-time monitoring noise,” including common industrial issues such as sensor noise, intermittent missing records, and abnormal peak spikes. This study applies a unified 7:1:2 time-series partitioning and missing value imputation method to CPMR-LD, using it alongside the first two datasets throughout the simulation experiments in Section 3.2.

The dimension of robustness testing is single: existing robustness tests focus on hyperparameter selection and ablation experiments, and do not involve tests on model structure perturbations or changes in data distribution; they also do not verify the performance degradation of the model in ultra-long-term predictions (such as quarterly or annual predictions). It is recommended to supplement robustness tests involving model structure replacement and data distribution adjustment; add ultra-long-term prediction experiments, analyze the laws of performance degradation, and propose optimization schemes.

Reply: Thank you for your review comments. In the revised paper, Table 4 (covering ultra-long-term projections for 6 and 12 months) validated the model's robustness, Table 6 assessed the robustness of structural substitutions and data distribution adjustments. Please see the revisions as follows:

**Table 4. Test results of different prediction models**

| Prediction Horizon | Model | P/% | R/% | F1/% | Mean Forecast Time / s | p-value |
| --- | --- | --- | --- | --- | --- | --- |
| 6 months | VMD-ResNet | 84.73 | 82.94 | 83.82 | 1.94 | 0.071 |
|  | EEMD-Attention | 86.04 | 84.63 | 85.32 | 2.26 | 0.064 |
|  | GA-Enhanced LSTM | 86.57 | 85.08 | 85.81 | 2.38 | 0.052 |
|  | PSO-BiLSTM | 87.46 | 86.02 | 86.73 | 2.23 | 0.041 |
|  | Transformer-DGNN | 88.12 | 87.24 | 87.68 | 1.83 | 0.034 |
|  | Our model | 92.08 | 90.97 | 91.52 | 1.59 | 0.008 |
| 12 months | VMD-ResNet | 81.18 | 79.36 | 80.26 | 2.04 | 0.049 |
|  | EEMD-Attention | 82.37 | 81.02 | 81.69 | 2.35 | 0.043 |
|  | GA-Enhanced LSTM | 83.04 | 81.68 | 82.35 | 2.47 | 0.037 |
|  | PSO-BiLSTM | 83.56 | 82.45 | 83 | 2.32 | 0.029 |
|  | Transformer-DGNN | 84.61 | 83.24 | 83.92 | 1.92 | 0.021 |
|  | Our model | 89.41 | 88.26 | 88.83 | 1.72 | 0.004 |

As shown in Table 4, as the prediction time domain is extended from 6 months to 12 months, the F1 of each baseline model has decreased to varying degrees. This is mainly due to the accumulation of trend drift in the long-term sequence, seasonal phase misalignment, and abnormal peak amplification, which have led to the gradual failure of short-term dependence. Meanwhile, optimization and enhancement models such as GA-LSTM and PSO-BiLSTM are sensitive to the initial values and are prone to fall into local optima in the long-term non-convex loss space. Therefore, the 12-month F1 only remains at 82% - 83%, and the p values are mostly at the boundary significance level of 0.03-0.05. The F1 of VMD-ResNet and EEMD-Attention further decreased to 80.26% and 81.69% respectively. Although Transformer-DGNN was slightly robust, the 12-month F1 was only 83.92%, p = 0.021. It indicates that its long-term modeling ability is still limited. In contrast, the 6-month and 12-month predicted F1 of the proposed model were 91.52% and 88.83% respectively, corresponding to p values of 0.008 and 0.004, which were significantly lower than 0.01. Its lower attenuation comes from cross-scale dependency modeling, global search suppressing local traps, and the automatic compensation of ECM for slow-changing trends. To address the remaining accuracy decline in long-term predictions, in the future, the cumulative error caused by trend shift can be further reduced by introducing more fine-grained trend decomposition (such as SSA or multi-scale wavelets), enhancing the periodic adaptive term of ECM, or adding long-term feature-specific perturbation operators in CLPO. Overall, this model still maintains stable performance under time-domain expansion, with the inference delay slightly increasing from 1.59 seconds to 1.72 seconds, meeting the requirements of scheduling and deployment.

**Table 6 Robustness evaluation under model structure replacement and data distribution alignment**

| Experiment Type | Replacement / Perturbation Description | MAE Change | RMSE Change | Stability (1−CV) |
| --- | --- | --- | --- | --- |
| Structure Replacement 1 | Replace BiLSTM with GRU | 3.10% | 3.80% | −2.4% |
| Structure Replacement 2 | Remove WR layer (use simple averaging) | 7.40% | 8.20% | −5.6% |
| Structure Replacement 3 | Replace CLPO with PSO | 5.90% | 6.70% | −4.1% |
| Distribution Alignment 1 | 20% reweighted resampling of training set | 2.60% | 2.90% | −1.7% |
| Distribution Alignment 2 | Adversarial perturbation to peak-load segments (±8%) | 4.80% | 5.20% | −3.3% |
| Distribution Alignment 3 | Mean-shift perturbation (+10% scaling) | 3.40% | 3.90% | −2.2% |

As shown in Table 6, the model maintains stable performance under various structural replacements and data distribution perturbations, with MAE and RMSE variations kept within 3%-8%—demonstrating that single-module replacements do not compromise overall performance. Notably, removing WR or substituting CLPO with PSO results in significant performance degradation, further validating WR's weighted robustness and CLPO's global search advantage. The data distribution adjustment test reveals that ECM effectively absorbs trend deviations and maintains stability metrics (1−CV) within acceptable ranges under peak load disturbances and distribution shifts, indicating the structure's strong distribution generalization capability and robustness in practical scheduling scenarios.

Non-standard use of academic terms: some terms lack clear definitions; some expressions are inconsistent. It is suggested to clarify the definitions and boundaries of core terms, unify the annotation format for the first occurrence of terms (such as full name + abbreviation), and improve the academic standardization of the paper.

Reply: Thank you for your review comments. The academic terms have been verified. Such as:

To this end, the study selected Temporal Convolutional Networks (TCN), Long Short-Term Memory (LSTM), and Bidirectional LSTM (BiLSTM) to propose a Combinatorial Deep Learning (CDL) model through Weighted Reconstruction (WR). Simultaneously, by integrating the Continuous Learning-based Parrot Optimization (CLPO) algorithm with a Long-Term Equilibrium Error Correction Model (ECM), an end-to-end power load forecasting framework was constructed.

Reviewer #4: (No Response)

Reply: Thank you
