# Supplementary material for: Power load forecasting combining deep learning models and improved CLPO algorithm
Source: PLoS One. 2026 Jun 29;21(6):e0351428. doi: 10.1371/journal.pone.0351428 (PMC13313379; doi:10.1371/journal.pone.0351428)
Supplement: Attachment — Submitted filename: Response_to_Reviewers_auresp_3.docx [file pone.0351428.s005.docx]

Reviewer #2:

Authors should seriously incorporate the comments provided in the previous round. I am not satisfied after reading R2.

Reply: Thank you very much for your comments. We have revised the paper carefully according to all the reviewers’ comments and suggestions in R1 and R2. Please review again. If you have any other concerns, please feel free to tell us. We will continue to carefully revise the paper. Thank you very much again.

Reviewer #5:

Overall, the manuscript demonstrates technical depth, methodological integration, and practical relevance. The proposed model improved predictive performance compared to several baseline methods, supported by experimental validation on public datasets.

However, several aspects require clarification and strengthening to fully meet PLOS ONE publication criteria, methodological transparency, and clarity of presentation.

Major Strengths

1. The study proposes a well-structured hybrid framework combining:

• Multi-scale deep learning (TCN, LSTM, BiLSTM)

• Adaptive feature fusion via WR

• Meta-heuristic optimization (CLPO)

• Error correction through ECM

This design is conceptually sound and addresses known limitations in single-model forecasting approaches.

Reply: Thank you very much for your positive evaluation for our work.

2. The manuscript includes:

• Ablation studies

• Benchmark comparisons with recent models

• Statistical validation (e.g., t-test with p < 0.05)

• Testing under extreme weather and varying load conditions

These elements enhance the credibility of the reported performance improvements

Reply: Thank you very much for your positive evaluation for our work.

3. The use of UK-NGED and EDF-SGD datasets improves transparency and allows for potential reproducibility and benchmark against existing studies

Reply: Thank you very much for your positive evaluation for our work.

4. The application to smart grid scheduling, renewable energy integration, and extreme scenarios demonstrates strong real-world applicability.

Reply: Thank you very much for your positive evaluation for our work.

5. Scientific validity: Generally sound, but requires clearer methodological transparency.

Reply: Thank you very much for your comments. The methodological framework flow has been further elaborated as follows:

To address the issue of unbalanced integration of different time-scale characteristics in power load forecasting and the tendency of optimization strategies to fall into local optima, the overall method process can be summarized as "data input - multi-scale feature extraction - adaptive fusion - parameter optimization - error correction - prediction output". Firstly, historical power load data and auxiliary variables such as meteorology and time are preprocessed and used as model inputs; secondly, in a combined deep learning framework, the time series convolution network is used to extract short-term fluctuation features, the long short-term memory network is used to model long-term dependencies, and the bidirectional long short-term memory network is used to enhance the bidirectional temporal feature expression of the sequence; subsequently, through the weighted aggregation method, the outputs of each sub-model are adaptively fused to achieve dynamic weight allocation of information from different time scales; on this basis, the continuous learning parrot optimization algorithm is introduced to jointly optimize the model weights and key parameters to enhance the global search ability and convergence stability; finally, through the long-term balanced error correction mechanism, the prediction residuals are dynamically compensated to output the final load forecasting result.

6. Data availability: Stated as available, but should be more explicitly verifiable.

Reply: Thank you very much for your comments. All data utilized were sourced from publicly available datasets (e.g., UK-NGED, EDF-SGD) and standardized experimental construction datasets. The relevant data sources, acquisition methods, and partitioning strategies are explicitly detailed in the manuscript, ensuring high accessibility and reproducibility.

Recommendation

1-

• Lack of implementation details (e.g., architecture depth, activation functions, batch size, training epochs)

Reply: Thank you very much for your comments. The model implementation details, including network layer count, activation functions, batch size, and training rounds, have been supplemented in Table 1 as follows:

To further enhance model reproducibility and engineering feasibility, key implementation details are supplemented as follows: The TCN component in the CDL framework adopts a 3-layer expanded convolutional architecture with 3-dimensional convolution kernels and expansion rates of 1, 2, and 4 respectively. Both LSTM and BiLSTM networks utilize a 2-layer stacked architecture with 128 hidden units per layer.

Table 1 Details of experimental environment and parameters

| Parameter | Configuration |
| --- | --- |
| Hardware Environment |  |
| CPU | Intel Core i9-13900K, base frequency 3.0 GHz |
| GPU | NVIDIA GeForce RTX 4090, 24 GB VRAM |
| Memory | 64 GB DDR5 |
| Operating system | Windows 11 Professional |
| Programming framework | Python 3.9, TensorFlow 2.12.0, PyTorch 1.13.1 |
| Model Architecture | |
| Deep learning model | TCN-LSTM-BiLSTM |
| TCN depth | 3 layers (dilation rates: 1, 2, 4; kernel size: 3) |
| LSTM depth | 2 stacked layers (hidden units: 128) |
| BiLSTM depth | 2 stacked layers (hidden units: 128) |
| Activation function | ReLU (hidden layers), Linear (output layer) |
| Training Settings | |
| Batch size | 64 |
| Training epochs | 300 (with Early Stopping) |
| Early stopping | Stop if validation loss does not decrease for 15 epochs |
| Optimizer | Adam |
| Initial learning rate | 0.001 |
| Learning rate strategy | Learning rate decay |
| Optimization Algorithm (CLPO) | |
| Optimization algorithm | CLPO |
| Population size | 200 |
| Max iterations | 500 |
| Mutation rate | 0.2 |
| Crossover rate | 0.75 |
| Selection strategy | Elitist selection |
| Constraint weight | 1.5 |
| Auxiliary Modules | |
| Error correction method | ECM |
| Weighted reconstruction method | WR |

To further enhance model reproducibility and engineering feasibility, key implementation details are supplemented as follows: The TCN component in the CDL framework adopts a 3-layer expanded convolutional architecture with 3-dimensional convolution kernels and expansion rates of 1, 2, and 4 respectively. Both LSTM and BiLSTM networks utilize a 2-layer stacked architecture with 128 hidden units per layer. The ReLU activation function is employed for neural networks, while linear activation functions are applied to the output layer to accommodate regression tasks. During model training, batch size is set to 64 with a maximum training cycle limit of 300, accompanied by an Early Stopping strategy that terminates training prematurely when validation set loss fails to show significant improvement for 15 consecutive cycles. The Adam optimizer is selected with an initial learning rate of 0.001, complemented by a learning rate decay mechanism to improve convergence stability.

• Need to mention to the code availability or repository link•

Reply: Thank you very much for your comments.

The code has been deposited in an established DOI-minting version as: https://zenodo.org/uploads/19595052.

• description of preprocessing steps for data insufficient

Reply: Thank you very much for your comments. The preprocessing steps for data insufficient (including missing value imputation, outlier handling, sample augmentation and normalization) have been supplemented, please see the detailed description in Section 3 as follows:

To address potential data insufficiency, missing values, and sampling imbalance in actual power load datasets, this study implements unified preprocessing on raw data prior to modeling. First, random missing values are filled using a combination of linear interpolation and sliding window mean filling, while continuous missing intervals are segmented through interpolation based on periodic characteristics of adjacent time periods to ensure temporal sequence continuity. Second, outliers are identified via a joint detection strategy combining boxplots and the 3σ principle, followed by local smoothing techniques to mitigate extreme noise interference during model training. Additionally, to alleviate sample distribution unevenness, resampling enhancement is applied to peak load scenarios and extreme weather events, ensuring stable learning performance across diverse load patterns. Finally, all input features undergo normalization processing to eliminate dimensionality discrepancies and improve model convergence efficiency.

Recommendation: support full experimental reproducibility details, including code or pseudo-code expansion, parameter settings, and preprocessing pipeline.

Reply: Thank you very much for your comments. The code, parameter settings and preprocessing pipeline have been provided, please see the above revisions.

The code has been deposited in an established DOI-minting version as: <https://zenodo.org/uploads/19595052>.

2- The Methods section is very dense, with:

• Excessive mathematical formulations without explanation

• Some overly long paragraphs

• Limited guidance for researchers to follow the study

Recommendation:

• boost structure using subheadings and explanations

Reply: Thank you very much for your comments. Some long paragraphs have been reorganized and restructured using subheadings and explanations. For example:

2.1.1 OVERALL PREDICTION PROCESS AND PROBLEM MODELING

2.1.2 LSTM AND BILSTM TIME SERIES MODELING MECHANISMS

**In addition, mathematical formulations have been explained in more details, for example；**

In equation (1), $v_{t}$ represents the convolution output at time $t$; $x_{u-i}$ represents the value of the input sequence at time $u-i$.

In equation (2), $R_{l}$ represents the receptive field size of the $l$ layer; $l$ represents the number of network layers.

In equation (3), $H_{l}$ represents the output feature of the $l$ layer; $H_{l-1 }$ represents the input feature of the $l-1$ layer; $W_{l}$ represents the convolution kernel weights of the $l$ layer; represents the convolution operation; $b_{l}$ represents the bias term; $f(\_)$ represents the nonlinear activation function.

• Provide a simplified summary for pipeline

Reply: Thank you very much for your comments. The methodological framework flow has been further elaborated to enable researchers to follows our srudy, as follows:

To address the issue of unbalanced integration of different time-scale characteristics in power load forecasting and the tendency of optimization strategies to fall into local optima, the overall method process can be summarized as "data input - multi-scale feature extraction - adaptive fusion - parameter optimization - error correction - prediction output". Firstly, historical power load data and auxiliary variables such as meteorology and time are preprocessed and used as model inputs; secondly, in a combined deep learning framework, the time series convolution network is used to extract short-term fluctuation features, the long short-term memory network is used to model long-term dependencies, and the bidirectional long short-term memory network is used to enhance the bidirectional temporal feature expression of the sequence; subsequently, through the weighted aggregation method, the outputs of each sub-model are adaptively fused to achieve dynamic weight allocation of information from different time scales; on this basis, the continuous learning parrot optimization algorithm is introduced to jointly optimize the model weights and key parameters to enhance the global search ability and convergence stability; finally, through the long-term balanced error correction mechanism, the prediction residuals are dynamically compensated to output the final load forecasting result.

• How interacts operationally for each component done (WR, CLPO, ECM)

Reply: Thank you very much for your comments. The interacts operation for each component done has been explained as follows:

As shown in Figure 7, WR, CLPO, and ECM are not independent modules at the operational level but form a tightly coupled relationship through a unified parameter space and closed-loop optimization mechanism. Specifically, WR serves as an intermediate fusion layer that performs weighted combination of outputs from TCN, LSTM, and BiLSTM, with its weight vector constituting a critical component of overall optimization variables. CLPO jointly optimizes WR weights, network hyperparameters, and ECM correction coefficients within this unified parameter space, achieving cross-module parameter coordination. During each iteration cycle, WR-generated predictions first calculate residuals, which are fed into ECM for error correction. The corrected results then participate in CLPO's fitness evaluation, forming a closed-loop update process of "prediction-residual-correction-reoptimization." Under this mechanism, WR manages multi-scale feature contribution allocation, ECM corrects systematic biases, and CLPO dynamically coordinates parameter updates through global search. This enables collaborative convergence under a unified objective function while avoiding suboptimal solutions caused by independent optimization of individual modules.

3. While the integration is extensive, the incremental novelty of each component is not sufficiently emphasized.

Recommendation:

• Distinguish what is new versus adapted in details•

Reply: Thank you very much for your comments. Compared to the existing studies, the innovations of our work are:

Compared with existing studies, the innovations of this paper are primarily reflected in the following three aspects:

(1) Theoretical Innovation: This study establishes a collaborative optimization modeling framework based on a unified objective function, integrating multi-scale feature representation, model fusion weights, parameter optimization processes, and error correction mechanisms into a unified optimization system. This creates a closed-loop interaction mechanism of "features-weighting-error". Unlike traditional staged modeling or post-processing correction methods, the framework theoretically achieves coupled optimization across multiple modules, transforming independent model optimization into an integrated collaborative convergence process. This approach significantly enhances the stability and consistency of modeling complex nonlinear load sequences.

(2) Technological Innovation: A parameter optimization strategy combining Weighted Reconstruction (WR) and Continuous Learning Parrot Optimization (CLPO) is proposed to achieve adaptive fusion of multi-model outputs and dynamic global parameter search. The WR mechanism enhances multi-scale feature integration through dynamic weight updates, while CLPO improves global search capability and convergence efficiency by incorporating continuous learning and adaptive parameter tuning strategies, effectively avoiding the tendency of traditional optimization algorithms to get trapped in local optima.

(3) Application Innovation: Designed for complex power load forecasting scenarios, we developed an integrated CDL-CLPO-ECM prediction system that deeply integrates forecasting models with error correction mechanisms, enabling an end-to-end closed-loop optimization process from data input to result refinement. This approach maintains stable predictive performance even under high volatility and extreme load conditions, providing an engineering-ready solution for power load forecasting in multi-source disturbance environments.

• support the study with stronger comparative discussion with closely related hybrid models

Reply: Thank you very much for your comments. The comparative discussion between the proposed methods and other existing approaches has been supplemented, as follows:

Furthermore, compared to existing hybrid models (such as CNN-LSTM, VMD-ResNet, and EEMD-Attention), the proposed method demonstrates improvements not only in model composition but also through incremental innovations in multi-module collaborative mechanisms. Traditional hybrid models often rely on fixed weight fusion or staged optimization strategies, which struggle to maintain dynamic balance across multi-scale features and are prone to local optima. In contrast, WR achieves adaptive weight allocation by introducing constraints and regularization terms, CLPO jointly optimizes WR weights and ECM parameters under a unified objective function, while ECM dynamically compensates for long-term trend drift at the output end, forming a closed-loop mechanism of "feature fusion-global optimization-error correction." Experimental results show that under extreme weather and high load fluctuation scenarios, the model's error fluctuations (standard deviation) are reduced by approximately 15%-25% compared to competing methods, with smoother convergence processes. These findings indicate that its advantages stem not only from structural complexity but also from enhanced inter-module collaborative optimization capabilities.

4. Although t-tests are declear, the study lacks:

• Confidence intervals

Reply: Thank you very much for your comments. The confidence intervals have been added as follows:

**Table 4. Test results of different prediction models**

| Prediction Horizon | Model | P/% | R/% | F1/% | Mean Forecast Time / s | *p*-value |
| --- | --- | --- | --- | --- | --- | --- |
| 6 months | VMD-ResNet | 84.73 ± 0.82 | 82.94 ± 0.91 | 83.82 ± 0.87 | 1.94 ± 0.06 | 0.071 |
|  | EEMD-Attention | 86.04 ± 0.76 | 84.63 ± 0.85 | 85.32 ± 0.80 | 2.26 ± 0.08 | 0.064 |
|  | GA-Enhanced LSTM | 86.57 ± 0.71 | 85.08 ± 0.79 | 85.81 ± 0.75 | 2.38 ± 0.07 | 0.052 |
|  | PSO-BiLSTM | 87.46 ± 0.68 | 86.02 ± 0.73 | 86.73 ± 0.70 | 2.23 ± 0.06 | 0.041 |
|  | Transformer-DGNN | 88.12 ± 0.63 | 87.24 ± 0.69 | 87.68 ± 0.66 | 1.83 ± 0.05 | 0.034 |
|  | Our model | 92.08 ± 0.52 | 90.97 ± 0.58 | 91.52 ± 0.55 | 1.59 ± 0.04 | 0.008 |
| 12 months | VMD-ResNet | 81.18 ± 0.93 | 79.36 ± 1.02 | 80.26 ± 0.98 | 2.04 ± 0.07 | 0.049 |
|  | EEMD-Attention | 82.37 ± 0.88 | 81.02 ± 0.95 | 81.69 ± 0.91 | 2.35 ± 0.09 | 0.043 |
|  | GA-Enhanced LSTM | 83.04 ± 0.84 | 81.68 ± 0.90 | 82.35 ± 0.87 | 2.47 ± 0.08 | 0.037 |
|  | PSO-BiLSTM | 83.56 ± 0.80 | 82.45 ± 0.86 | 83.00 ± 0.83 | 2.32 ± 0.07 | 0.029 |
|  | Transformer-DGNN | 84.61 ± 0.75 | 83.24 ± 0.82 | 83.92 ± 0.79 | 1.92 ± 0.05 | 0.021 |
|  | Our model | 89.41 ± 0.61 | 88.26 ± 0.68 | 88.83 ± 0.65 | 1.72 ± 0.04 | 0.004 |

• Variance analysis with runs

Reply: Thank you very much for your comments. The variance analysis with runs has been provided, for example:

As shown in Table 5, under sunny conditions, the proposed model achieves MAPE of 2.92% ± 0.27% and RMSE of 2.45 ± 0.23 kW, outperforming all baselines with lower variance. Under cloudy and stormy conditions, although all models show increased errors, the proposed method maintains smaller standard deviations (e.g., MAPE std reduced by ~15%–25%), indicating stronger robustness to weather disturbances. In storm scenarios, its MAPE remains at 6.42% ± 0.55%, still lower than Transformer-DGNN. Additionally, throughput and response time remain stable (1.38–1.72 s), demonstrating consistent computational efficiency. These results confirm that the model maintains both accuracy and stability under complex and uncertain load conditions.

Recommendation:

Include additional metrics and robustness checks to support the validity of conclusions.

Reply: Thank you very much for your comments. The confidence intervals and variance analysis have been provided, please see the above revisions.

In addition, a systematic evaluation of model performance (prediction accuracy, stability, and computational efficiency) from multiple dimensions has been conducted out, including metrics such as MAPE, RMSE, F1 score, throughput, and response time. Robustness validation has been performed under various complex scenarios, including extreme weather events, fluctuations in renewable energy penetration rates, different load patterns, and distributional disturbances. The reliability of results was statistically supported through repeated experiments and significance tests (p <0.05). Therefore, the existing evaluation framework effectively validates the conclusions' robustness, while additional indicators may lead to information redundancy rather than substantial improvements.

5. A thorough language revision is required to be more publication standards.

Reply: Thank you very much for your comments. The language has been improved by scholars who have published high level English papers.

6.-

• Clarify all abbreviations, only at the first of use (e.g., CDL, ECM, WR).•

Reply: Thank you very much for your comments. The abbreviations have been defined as follows:

**LIST OF ABBREVIATIONS**

ARIMA: Autoregressive Integrated Moving Average

TCN: Temporal Convolutional Network

LSTM: Long Short-Term Memory

BiLSTM: Bidirectional Long Short-Term Memory

RNN: Recurrent Neural Network

WR: Weighted Reconstruction

CDL: Combinatorial Deep Learning

CLPO: Continuous Learning-based Parrot Optimization

ECM: Error Correction Model

RMSE: Root Mean Square Error

MAE: Mean Absolute Error

MAPE: Mean Absolute Percentage Error

PO: Parrot Optimizer

DDIWC: Dynamically Decreasing Inertia Weight Control

CPU: Central Processing Unit

GPU: Graphics Processing Unit

UK-NGED: UK National Grid Electricity Demand Dataset

EDF-SG: Electricité de France Smart Grid Dataset

VMD-ResNet: Variational Mode Decomposition and Deep Residual Network Model

Transformer-DGNN: Transformer and Dynamic Graph Neural Network Model

P: Precision

R: Recall

CPMR-LD: China Provincial Multi-Region Load Dataset

PSO: Particle Swarm Optimization

• Improve captions for figures, and the figures 3–7 require clearer explanation.

Reply: Thank you very much for your comments. The captions and explanation of the Figures have been modified. Take the explanation of Figure 7 as an example:

Figure 3. Multi-scale feature fusion and weight adaptation mechanism in the CDL framework based on WR

Figure 4. Multi-strategy search mechanism of the PO algorithm including foraging, residence, and communication behaviors

Figure 5. Improved CLPO optimization process with chaotic initialization and adaptive parameter update strategy

Figure 6. Error correction mechanism based on ECM integrated with BiLSTM prediction outputs

Figure 7 Integrated CDL-CLPO-ECM framework with joint optimization and closed-loop correction

**The explanation of Figure 7:**

As shown in Figure 7, WR, CLPO, and ECM are not independent modules at the operational level but form a tightly coupled relationship through a unified parameter space and closed-loop optimization mechanism. Specifically, WR serves as an intermediate fusion layer that performs weighted combination of outputs from TCN, LSTM, and BiLSTM, with its weight vector constituting a critical component of overall optimization variables. CLPO jointly optimizes WR weights, network hyperparameters, and ECM correction coefficients within this unified parameter space, achieving cross-module parameter coordination. During each iteration cycle, WR-generated predictions first calculate residuals, which are fed into ECM for error correction. The corrected results then participate in CLPO's fitness evaluation, forming a closed-loop update process of "prediction-residual-correction-reoptimization." Under this mechanism, WR manages multi-scale feature contribution allocation, ECM corrects systematic biases, and CLPO dynamically coordinates parameter updates through global search. This enables collaborative convergence under a unified objective function while avoiding suboptimal solutions caused by independent optimization of individual modules.

• Some claims (e.g., “high practical value”) should be supported with more concrete evidence.

Reply: Thank you very much for your comments. More concrete evidences have been provided as follows:

Compared with existing methods, the proposed model improves joint modeling of multi-scale dependencies, achieving Root Mean Square Error (RMSE) reductions of about 7%–13% and F1-score gains of 3%–8%. While improvements in routine scenarios are moderate, error reduction exceeds 15% under extreme conditions, demonstrating stronger robustness and generalization for practical deployment.

• Data availability: Stated as available, but should be more explicitly verifiable.

Reply: Thank you very much for your comments. All data utilized were sourced from publicly available datasets (e.g., UK-NGED, EDF-SGD) and standardized experimental construction datasets. Please see details as follows:

The study utilizes two publicly available electricity load datasets as test sources. The first is the UK National Grid Electricity Demand Dataset (UK-NGED), accessible at https://connecteddata.nationalgrid.co.uk/?utm_source=chatgpt.com. Provided by the UK National Grid operator, this dataset covers national electricity load information from 2015 to 2023 with 30-minute recording intervals, incorporating multidimensional variables such as temperature, wind speed, humidity, holiday indicators, and day-night labels.

The second dataset is the Electricité de France Smart Grid Dataset (EDF-SGD), available at https://www.neso.energy/data-portal/historic-demand-data?utm_source=chatgpt.com. Published by EDF, this dataset contains detailed load records for over 20,000 residential households and thousands of commercial and industrial users, with hourly and daily granularity spanning 2017 to 2022. Beyond load time series, it provides additional data including temperature, humidity, light intensity, electricity consumption category labels, and penetration ratios of renewable energy sources such as solar and wind power. These features enable effective modeling of electricity consumption behavior and are suitable for predictive evaluation in high penetration scenarios [36].

7.- Scientific validity: Generally sound, but requires clearer methodological transparency

Reply: Thank you very much for your comments. The methodological framework flow has been further elaborated as follows:

To address the issue of unbalanced integration of different time-scale characteristics in power load forecasting and the tendency of optimization strategies to fall into local optima, the overall method process can be summarized as "data input - multi-scale feature extraction - adaptive fusion - parameter optimization - error correction - prediction output". Firstly, historical power load data and auxiliary variables such as meteorology and time are preprocessed and used as model inputs; secondly, in a combined deep learning framework, the time series convolution network is used to extract short-term fluctuation features, the long short-term memory network is used to model long-term dependencies, and the bidirectional long short-term memory network is used to enhance the bidirectional temporal feature expression of the sequence; subsequently, through the weighted aggregation method, the outputs of each sub-model are adaptively fused to achieve dynamic weight allocation of information from different time scales; on this basis, the continuous learning parrot optimization algorithm is introduced to jointly optimize the model weights and key parameters to enhance the global search ability and convergence stability; finally, through the long-term balanced error correction mechanism, the prediction residuals are dynamically compensated to output the final load forecasting result.

- Data availability: Stated as available, but should be more explicitly verifiable.

Reply: Thank you very much for your comments. All data utilized were sourced from publicly available datasets (e.g., UK-NGED, EDF-SGD) and standardized experimental construction datasets. The relevant data sources, acquisition methods, and partitioning strategies are explicitly detailed in the manuscript, ensuring high accessibility and reproducibility.
