# Supplementary material for: Power load forecasting combining deep learning models and improved CLPO algorithm
Source: PLoS One. 2026 Jun 29;21(6):e0351428. doi: 10.1371/journal.pone.0351428 (PMC13313379; doi:10.1371/journal.pone.0351428)
Supplement: Peer review history

# Peer review: Decision Letter 0

**DOI:** 10.1371/journal.pone.0351428.r001

**Authors:** Zeyar Aung

5 Sep 2025

PONE-D-25-31195

Power Load Forecasting Combining Deep Learning Models and Improved CLPO Algorithm

PLOS ONE

Dear Dr. Wang,

Thank you for submitting your manuscript to PLOS ONE. After careful consideration, we feel that it has merit but does not fully meet PLOS ONE’s publication criteria as it currently stands. Therefore, we invite you to submit a revised version of the manuscript that addresses the points raised during the review process.

==============================

**Major revision:**

The paper needs improvements in its technical contents and its presentation.

Please address the comments by the reviewers.

==============================

Please submit your revised manuscript by Oct 20 2025 11:59PM. If you will need more time than this to complete your revisions, please reply to this message or contact the journal office at plosone@plos.org. When you're ready to submit your revision, log on to https://www.editorialmanager.com/pone/ and select the 'Submissions Needing Revision' folder to locate your manuscript file.

Please include the following items when submitting your revised manuscript:

- A rebuttal letter that responds to each point raised by the academic editor and reviewer(s). You should upload this letter as a separate file labeled 'Response to Reviewers'.
- A marked-up copy of your manuscript that highlights changes made to the original version. You should upload this as a separate file labeled 'Revised Manuscript with Track Changes'.
- An unmarked version of your revised paper without tracked changes. You should upload this as a separate file labeled 'Manuscript'.

If you would like to make changes to your financial disclosure, please include your updated statement in your cover letter. Guidelines for resubmitting your figure files are available below the reviewer comments at the end of this letter.

If applicable, we recommend that you deposit your laboratory protocols in protocols.io to enhance the reproducibility of your results. Protocols.io assigns your protocol its own identifier (DOI) so that it can be cited independently in the future. For instructions see: https://journals.plos.org/plosone/s/submission-guidelines#loc-laboratory-protocols. Additionally, PLOS ONE offers an option for publishing peer-reviewed Lab Protocol articles, which describe protocols hosted on protocols.io. Read more information on sharing protocols at https://plos.org/protocols?utm_medium=editorial-email&utm_source=authorletters&utm_campaign=protocols.

We look forward to receiving your revised manuscript.

Kind regards,

Zeyar Aung

Academic Editor

PLOS ONE

Journal Requirements:

When submitting your revision, we need you to address these additional requirements.

1. Please ensure that your manuscript meets PLOS ONE's style requirements, including those for file naming. The PLOS ONE style templates can be found at

https://journals.plos.org/plosone/s/file?id=wjVg/PLOSOne_formatting_sample_main_body.pdf and

https://journals.plos.org/plosone/s/file?id=ba62/PLOSOne_formatting_sample_title_authors_affiliations.pdf.

2. Please note that PLOS One has specific guidelines on code sharing for submissions in which author-generated code underpins the findings in the manuscript. In these cases, we expect all author-generated code to be made available without restrictions upon publication of the work. Please review our guidelines at https://journals.plos.org/plosone/s/materials-and-software-sharing#loc-sharing-code and ensure that your code is shared in a way that follows best practice and facilitates reproducibility and reuse.

3. We note that your Data Availability Statement is currently as follows: [All relevant data are within the manuscript and its Supporting Information files.]

Please confirm at this time whether or not your submission contains all raw data required to replicate the results of your study. Authors must share the “minimal data set” for their submission. PLOS defines the minimal data set to consist of the data required to replicate all study findings reported in the article, as well as related metadata and methods (https://journals.plos.org/plosone/s/data-availability#loc-minimal-data-set-definition).

For example, authors should submit the following data:

- The values behind the means, standard deviations and other measures reported;

- The values used to build graphs;

- The points extracted from images for analysis.

Authors do not need to submit their entire data set if only a portion of the data was used in the reported study.

If your submission does not contain these data, please either upload them as Supporting Information files or deposit them to a stable, public repository and provide us with the relevant URLs, DOIs, or accession numbers. For a list of recommended repositories, please see https://journals.plos.org/plosone/s/recommended-repositories.

If there are ethical or legal restrictions on sharing a de-identified data set, please explain them in detail (e.g., data contain potentially sensitive information, data are owned by a third-party organization, etc.) and who has imposed them (e.g., an ethics committee). Please also provide contact information for a data access committee, ethics committee, or other institutional body to which data requests may be sent. If data are owned by a third party, please indicate how others may request data access.

4 Thank you for stating the following in the Financial Disclosure section:

“The author(s) received no specific funding for this work.”

We note that one or more of the authors are employed by a commercial company

a.        Please provide an amended Funding Statement declaring this commercial affiliation, as well as a statement regarding the Role of Funders in your study. If the funding organization did not play a role in the study design, data collection and analysis, decision to publish, or preparation of the manuscript and only provided financial support in the form of authors' salaries and/or research materials, please review your statements relating to the author contributions, and ensure you have specifically and accurately indicated the role(s) that these authors had in your study. You can update author roles in the Author Contributions section of the online submission form.

Please also include the following statement within your amended Funding Statement.

“The funder provided support in the form of salaries for authors [insert relevant initials], but did not have any additional role in the study design, data collection and analysis, decision to publish, or preparation of the manuscript. The specific roles of these authors are articulated in the ‘author contributions’ section.”

If your commercial affiliation did play a role in your study, please state and explain this role within your updated Funding Statement.

b. Please also provide an updated Competing Interests Statement declaring this commercial affiliation along with any other relevant declarations relating to employment, consultancy, patents, products in development, or marketed products, etc.

Within your Competing Interests Statement, please confirm that this commercial affiliation does not alter your adherence to all PLOS ONE policies on sharing data and materials by including the following statement: "This does not alter our adherence to  PLOS ONE policies on sharing data and materials.” (as detailed online in our guide for authors http://journals.plos.org/plosone/s/competing-interests) . If this adherence statement is not accurate and there are restrictions on sharing of data and/or materials, please state these. Please note that we cannot proceed with consideration of your article until this information has been declared.

Please include both an updated Funding Statement and Competing Interests Statement in your cover letter. We will change the online submission form on your behalf.

5. If the reviewer comments include a recommendation to cite specific previously published works, please review and evaluate these publications to determine whether they are relevant and should be cited. There is no requirement to cite these works unless the editor has indicated otherwise.

[Note: HTML markup is below. Please do not edit.]

Reviewers' comments:

Reviewer's Responses to Questions

**Comments to the Author**

1. Is the manuscript technically sound, and do the data support the conclusions?

The manuscript must describe a technically sound piece of scientific research with data that supports the conclusions. Experiments must have been conducted rigorously, with appropriate controls, replication, and sample sizes. The conclusions must be drawn appropriately based on the data presented.

Reviewer #1: Yes

Reviewer #2: Yes

**********

2. Has the statistical analysis been performed appropriately and rigorously?

Reviewer #1: Yes

Reviewer #2: N/A

**********

3. Have the authors made all data underlying the findings in their manuscript fully available?

The PLOS Data policy requires authors to make all data underlying the findings described in their manuscript fully available without restriction, with rare exception (please refer to the Data Availability Statement in the manuscript PDF file). The data should be provided as part of the manuscript or its supporting information, or deposited to a public repository. For example, in addition to summary statistics, the data points behind means, medians and variance measures should be available. If there are restrictions on publicly sharing data—e.g. participant privacy or use of data from a third party—those must be specified.

Reviewer #1: Yes

Reviewer #2: Yes

**********

4. Is the manuscript presented in an intelligible fashion and written in standard English?

PLOS ONE does not copyedit accepted manuscripts, so the language in submitted articles must be clear, correct, and unambiguous. Any typographical or grammatical errors should be corrected at revision, so please note any specific errors here.

Reviewer #1: Yes

Reviewer #2: Yes

**********

5. Review Comments to the Author

Please use the space provided to explain your answers to the questions above. You may also include additional comments for the author, including concerns about dual publication, research ethics, or publication ethics. (Please upload your review as an attachment if it exceeds 20,000 characters)

Reviewer #1: While the technical depth and innovation are commendable, a few minor changes are recommended to strengthen the manuscript.

First, given the complexity of the model, the paper would benefit from a short discussion on model interpretability or potential explainability strategies, especially since power grid operators may need insights into prediction rationale.

Second, although the paper discusses inference efficiency, there is limited commentary on training time or computational overhead introduced by the model's multi-layered structure. Clarifying this aspect would improve understanding of its scalability for real-time or embedded deployment.

Third, the feature set focuses on technical inputs such as load, weather, and time indicators. The inclusion or future consideration of behavioral or economic variables could be acknowledged, as such factors are increasingly used in modern load forecasting.

Finally, minor language revisions are suggested to enhance readability and clarity, especially in sections describing algorithmic flow and dataset characteristics.

Reviewer #2: This paper presents a power load forecasting method that combines deep learning models (TCN-LSTM-BiLSTM) with an improved Continuous Learning-based Parrot Optimization (CLPO) algorithm and Error Correction Model (ECM) to enhance prediction accuracy in complex load environments. The authors propose a Combined Deep Learning (CDL) framework that uses weighted reconstruction to integrate multiple model outputs and applies parrot optimization for hyperparameter tuning and joint error correction for improved long-term forecasting performance. While the work addresses a relevant problem in power system management, it suffers from fundamental methodological flaws and lacks rigorous theoretical foundations that severely compromise its scientific contribution.

Comment 1: The theoretical justification for combining TCN, LSTM, and BiLSTM architectures lacks rigorous mathematical foundation and appears to be an ad-hoc integration of existing techniques without demonstrating fundamental advantages over individual components or established ensemble methods. The authors claim that CDL combines parallel computing capability of TCN with long memory advantages of LSTM and bidirectional characteristics of BiLSTM, but they do not provide mathematical analysis of how these architectures complement each other, how information flows between different temporal modeling mechanisms, or why this specific combination is optimal for power load forecasting compared to other sequence modeling approaches.

Comment 2: The experimental validation methodology exhibits critical limitations that undermine the credibility of the reported performance improvements, particularly the absence of comprehensive baseline comparisons and inadequate statistical validation across diverse load scenarios. The authors claim prediction accuracy exceeding 0.85 after 200 iterations but do not provide detailed experimental protocols, cross-validation procedures, or comparison with recent state-of-the-art power forecasting methods including transformer-based approaches, attention mechanisms, or other hybrid optimization techniques that are directly relevant to their architectural choices.

Comment 3: The Continuous Learning-based Parrot Optimization algorithm lacks detailed algorithmic specification and convergence analysis that are essential for understanding its practical implementation and theoretical guarantees. Pl follow the work The authors mention using CLPO for dynamic weight adjustment and parameter optimization but do not provide mathematical formulation of the optimization objective function, convergence criteria, or analysis of how the parrot optimization metaheuristic specifically adapts to the power load forecasting problem. The relationship between optimization iterations and forecasting performance improvement is not rigorously established.

Comment 4: The weighted reconstruction mechanism for combining multiple model outputs lacks principled design criteria and theoretical analysis of how different models contribute to the final prediction under varying load conditions. The authors describe using dynamic weight adjustment through equation (7) but do not provide analysis of how weights are learned during training, how the system handles conflicting predictions from different models, or how the weighting strategy adapts to different load patterns such as peak demand periods, seasonal variations, or emergency scenarios that are critical in power system operations. Pl follow the study efficient deepfake detection via layer-frozen assisted dual attention network for consumer imaging devices.

Comment 5: The Error Correction Model integration appears disconnected from the main deep learning framework and lacks detailed analysis of how long-term equilibrium relationships are established and maintained during dynamic load forecasting. The authors mention using ECM for joint error correction but do not provide mathematical formulation of how short-term prediction errors are corrected using long-term dependencies, how the error correction mechanism interacts with the neural network training process, or how this approach handles non-stationary load patterns that violate traditional error correction assumptions. Please follow the work bilateral feature fusion with hexagonal attention for robust saliency detection under uncertain environments.

Comment 6: The practical applicability for real-world power system deployment remains inadequately demonstrated despite claims about efficiency and reliability in complex load environments. The authors do not provide analysis of computational requirements for real-time forecasting, scalability to large-scale power grids, or robustness to data quality issues such as missing measurements, sensor failures, or communication delays that are common in practical power system monitoring. The system’s performance under extreme weather conditions, equipment outages, or other operational disruptions that significantly affect load patterns is not evaluated. Pl follow attention enhanced machine instinctive vision with human-inspired saliency detection.

Technical Questions:

1. How does the weighted reconstruction mechanism in equation (7) specifically handle the optimization of weights ωi when the three models (TCN, LSTM, BiLSTM) produce conflicting predictions during volatile load periods, and what is the mathematical relationship between the regularization parameter λ and the system’s ability to maintain prediction stability under varying load dynamics?

1. What is the exact algorithmic specification for the Continuous Learning-based Parrot Optimization process, particularly regarding how the parrot swarm intelligence mechanism adapts to the high-dimensional hyperparameter space of the combined deep learning models, and how does the optimization process ensure convergence while avoiding local minima in the complex loss landscape of power load forecasting?

**********

6. PLOS authors have the option to publish the peer review history of their article (what does this mean?). If published, this will include your full peer review and any attached files.

If you choose “no”, your identity will remain anonymous but your review may still be made public.

**Do you want your identity to be public for this peer review?**  For information about this choice, including consent withdrawal, please see our Privacy Policy.

Reviewer #1: No

Reviewer #2: No

**********

[NOTE: If reviewer comments were submitted as an attachment file, they will be attached to this email and accessible via the submission site. Please log into your account, locate the manuscript record, and check for the action link "View Attachments". If this link does not appear, there are no attachment files.]

While revising your submission, please upload your figure files to the Preflight Analysis and Conversion Engine (PACE) digital diagnostic tool, https://pacev2.apexcovantage.com/. PACE helps ensure that figures meet PLOS requirements. To use PACE, you must first register as a user. Registration is free. Then, login and navigate to the UPLOAD tab, where you will find detailed instructions on how to use the tool. If you encounter any issues or have any questions when using PACE, please email PLOS at figures@plos.org. Please note that Supporting Information files do not need this step.

# Author comment: Author response to Decision Letter 1

**DOI:** 10.1371/journal.pone.0351428.r002

28 Sep 2025

The manuscript has improved the quality according to comments

Attachment Submitted filename: Response to comments.docx

# Peer review: Decision Letter 1

**DOI:** 10.1371/journal.pone.0351428.r003

**Authors:** Zeyar Aung, Zeyar Aung

15 Nov 2025

PONE-D-25-31195R1

Power Load Forecasting Combining Deep Learning Models and Improved CLPO Algorithm

PLOS ONE

Dear Dr. Wang,

Thank you for submitting your manuscript to PLOS ONE. After careful consideration, we feel that it has merit but does not fully meet PLOS ONE’s publication criteria as it currently stands. Therefore, we invite you to submit a revised version of the manuscript that addresses the points raised during the review process.

Please submit your revised manuscript by Dec 30 2025 11:59PM. If you will need more time than this to complete your revisions, please reply to this message or contact the journal office at plosone@plos.org. When you're ready to submit your revision, log on to https://www.editorialmanager.com/pone/ and select the 'Submissions Needing Revision' folder to locate your manuscript file.

Please include the following items when submitting your revised manuscript:

- A rebuttal letter that responds to each point raised by the academic editor and reviewer(s). You should upload this letter as a separate file labeled 'Response to Reviewers'.
- A marked-up copy of your manuscript that highlights changes made to the original version. You should upload this as a separate file labeled 'Revised Manuscript with Track Changes'.
- An unmarked version of your revised paper without tracked changes. You should upload this as a separate file labeled 'Manuscript'.

If you would like to make changes to your financial disclosure, please include your updated statement in your cover letter. Guidelines for resubmitting your figure files are available below the reviewer comments at the end of this letter.

If applicable, we recommend that you deposit your laboratory protocols in protocols.io to enhance the reproducibility of your results. Protocols.io assigns your protocol its own identifier (DOI) so that it can be cited independently in the future. For instructions see: https://journals.plos.org/plosone/s/submission-guidelines#loc-laboratory-protocols. Additionally, PLOS ONE offers an option for publishing peer-reviewed Lab Protocol articles, which describe protocols hosted on protocols.io. Read more information on sharing protocols at https://plos.org/protocols?utm_medium=editorial-email&utm_source=authorletters&utm_campaign=protocols.

We look forward to receiving your revised manuscript.

Kind regards,

Zeyar Aung

Academic Editor

PLOS ONE

Journal Requirements:

1. If the reviewer comments include a recommendation to cite specific previously published works, please review and evaluate these publications to determine whether they are relevant and should be cited. There is no requirement to cite these works unless the editor has indicated otherwise.

Additional Editor Comments:

**Major revision:**

The authors still need to carry out a major revision based on the comments by the reviewers. Thank you.

[Note: HTML markup is below. Please do not edit.]

Reviewer's Responses to Questions

**Comments to the Author**

1. If the authors have adequately addressed your comments raised in a previous round of review and you feel that this manuscript is now acceptable for publication, you may indicate that here to bypass the “Comments to the Author” section, enter your conflict of interest statement in the “Confidential to Editor” section, and submit your "Accept" recommendation.

Reviewer #2: All comments have been addressed

Reviewer #3: (No Response)

Reviewer #4: All comments have been addressed

**********

2. Is the manuscript technically sound, and do the data support the conclusions?

The manuscript must describe a technically sound piece of scientific research with data that supports the conclusions. Experiments must have been conducted rigorously, with appropriate controls, replication, and sample sizes. The conclusions must be drawn appropriately based on the data presented.

Reviewer #2: Yes

Reviewer #3: Yes

Reviewer #4: Yes

**********

3. Has the statistical analysis been performed appropriately and rigorously?

Reviewer #2: N/A

Reviewer #3: Yes

Reviewer #4: Yes

**********

4. Have the authors made all data underlying the findings in their manuscript fully available?

The PLOS Data policy requires authors to make all data underlying the findings described in their manuscript fully available without restriction, with rare exception (please refer to the Data Availability Statement in the manuscript PDF file). The data should be provided as part of the manuscript or its supporting information, or deposited to a public repository. For example, in addition to summary statistics, the data points behind means, medians and variance measures should be available. If there are restrictions on publicly sharing data—e.g. participant privacy or use of data from a third party—those must be specified.

Reviewer #2: Yes

Reviewer #3: Yes

Reviewer #4: Yes

**********

5. Is the manuscript presented in an intelligible fashion and written in standard English?

PLOS ONE does not copyedit accepted manuscripts, so the language in submitted articles must be clear, correct, and unambiguous. Any typographical or grammatical errors should be corrected at revision, so please note any specific errors here.

Reviewer #2: Yes

Reviewer #3: Yes

Reviewer #4: Yes

**********

6. Review Comments to the Author

Please use the space provided to explain your answers to the questions above. You may also include additional comments for the author, including concerns about dual publication, research ethics, or publication ethics. (Please upload your review as an attachment if it exceeds 20,000 characters)

Reviewer #2: The paper requires substantial revisions including complete ablation studies isolating each component’s contribution, detailed mathematical formulation of the ECM mechanism, comprehensive experimental section with proper baselines and statistical validation, computational complexity analysis, and discussion of practical deployment considerations before it can be considered for publication.

Comment 1: The paper proposes a CDL (Convolutional Deep Learning) model combining TCN-LSTM-BiLSTM with improved CLPO (Continuous Learning Parrot Optimization) algorithm and ECM (Error Correction Mechanism) for power load forecasting, but the architectural justification for this specific multi-component combination lacks systematic ablation studies demonstrating necessity. While the abstract claims prediction accuracy exceeding 0.85 after 200 iterations with minimum loss of 0.05, the paper does not provide controlled experiments isolating contributions of TCN versus LSTM versus BiLSTM components individually. The Weighted Reconstruction (WR) mechanism in Equation (7) dynamically combines three sub-model outputs through weight optimization, yet the paper does not explain why this specific fusion approach outperforms simpler ensemble methods (averaging, voting, stacking) or analyze computational overhead from maintaining three parallel prediction pathways. The claim that “TCN weight increases during short-term spikes, LSTM dominates in seasonal trends, while BiLSTM provides bidirectional correction” requires empirical validation through weight trajectory analysis across different load scenarios.

Comment 2: The Weighted Reconstruction (WR) mechanism employing regularized least-squares optimization with multi-scale complementarity and risk minimization constraints (Equation 8) introduces substantial mathematical complexity without clear justification for why this formulation achieves Pareto-optimal equilibrium. The regularization parameter λ is described as balancing prediction accuracy and stability by suppressing extreme weight deviations when large and allowing concentrated weights when small, yet the paper provides no systematic analysis of λ selection, sensitivity to this hyperparameter, or validation that the claimed Pareto-optimality holds across different load dynamics. The statement that WR “decomposes the risk function into a prediction error term and a stability penalty term” suggests a principled approach, but the actual implementation details—how risk is quantified, how stability penalty is computed, and how the optimization converges—are insufficiently explained. The mathematical relationship in Equation (8) showing summation over time steps and models lacks clarity about whether this represents batch optimization or online updating.

Comment 3: The integration of CLPO (Continuous Learning Parrot Optimization) algorithm for weight matrix optimization claims to address limitations of gradient descent methods falling into local optima when handling complex nonlinear time series data, but the connection between parrot foraging behavior and power load forecasting optimization landscape remains unconvincing. Figure 4 shows the PO algorithm flow with parameter initialization, agent initialization, fitness calculation, and three behaviors (foraging, lingering, communication), yet the paper does not explain how these biologically-inspired mechanisms translate to effective exploration of the weight space or why this particular metaheuristic outperforms established alternatives (particle swarm optimization, genetic algorithms, simulated annealing). The claim that CLPO enables “joint error correction” and “dynamic error compensation mechanism” suggests integration with ECM, but Section 2.2 describing this integration is referenced without providing the actual methodology. The statement that the model “converges faster” and approaches 0.95 accuracy needs comparison with convergence rates of gradient-based optimization under identical conditions.

Comment 4: The Error Correction Mechanism (ECM) proposing to correct short-term prediction errors using long-term equilibrium relationships is mentioned prominently in the title and abstract but inadequately described in the methodology section. The paper references “CDL-CLPO-ECM” as the integrated framework but Section 2.2 promising to explain this optimization lacks the actual ECM formulation, mathematical foundation, or implementation details. Classical ECM in econometrics addresses cointegration relationships between variables, but how this concept applies to correcting multi-step-ahead power load forecasts from the CDL model output remains unclear. The claim that ECM performs “joint error correction” and introduces “dynamic error compensation mechanism at the prediction output” suggests post-processing adjustment, yet the paper does not specify whether ECM operates on raw predictions, weighted predictions, or final ensemble outputs, nor does it provide the correction equations or adaptation rules. Follow the study attention enhanced machine instinctive vision with human-inspired saliency detection

Comment 5: The experimental validation claims 15.3% reduction in long-term prediction error, 25.1% reduction during high load fluctuations, and 15.2% decrease in root mean square error under extreme weather with response time shortened to 1.72 seconds, but these impressive results lack critical context about baseline comparisons, dataset characteristics, and statistical significance. The paper mentions testing under “different load fluctuation environments” and “extreme weather conditions” without specifying the actual datasets used, train-test split ratios, evaluation protocols, or whether results represent single runs or averaged over multiple trials with confidence intervals. The comparison with “existing methods” in the introduction cites various LSTM-based and optimization-enhanced approaches achieving specific MAE or MSE improvements, but Section 3 (presumably containing experimental results) is not included in the provided excerpt, making it impossible to verify whether the proposed method is compared against these same baselines under identical conditions. The 200-iteration convergence mentioned suggests substantial computational cost that is not quantified through training time, inference latency, or hardware requirements. You can follow the lightweight transformer lightweight transformer-driven multi-scale trapezoidal attention network for saliency detection

Comment 6: The practical applicability of the proposed framework for real-time smart grid operations remains questionable given the architectural complexity combining three deep learning models with metaheuristic optimization requiring iterative fitness evaluation. The abstract claims the model “can maintain high accuracy prediction in variable load environments and has stronger adaptability to extreme penetration rates of new energy and extreme weather conditions,” yet provides no analysis of model robustness to concept drift, adaptation mechanisms for non-stationary load patterns, or retraining strategies when performance degrades. You can follow optimal features driven hybrid attention network for effective video summarization. The statement about 1.72-second response time suggests computational efficiency, but this metric is meaningless without specifying forecast horizon (1-hour ahead, 24-hour ahead, weekly), input sequence length, and whether this represents single-sample inference or batch processing. Power system operators require not only point predictions but also uncertainty quantification, probabilistic forecasts, and interpretable explanations for scheduling decisions—aspects completely absent from this work. The integration of WR weight optimization with CLPO appears to require solving an optimization problem for each prediction, raising serious concerns about scalability for operational deployment where thousands of forecasts must be generated rapidly.

Reviewer #3: Revisions

This paper addresses the core challenges in power load forecasting—namely the complex nonlinear characteristics of load data and the tendency of traditional methods to fall into local optima. The research topic holds both engineering practical value and academic significance, aligning with the practical needs of smart grid dispatch and optimization. The core innovation of the paper lies in constructing an end-to-end framework integrating Combined Deep Learning (CDL) - Improved Continuous Learning-based Parrot Optimization (CLPO) - Error Correction Model (ECM).

Nevertheless, at this stage, the manuscript is not yet ready for publication. Below are some of my specific comments. The manuscript requires a comprehensive review and careful revision by the authors to meet basic standards of clarity, readability, and academic rigor for financial time series research.

There are obvious flaws in the format specifications: some formula symbols are expressed inconsistently (for example, Equation 20 describes "A represents the final composite representation, and B, C, D represent dynamic weights", with redundant text); It is recommended to unify the definitions of formula symbols and reduce repetitive descriptions.

Insufficient data generalization and representativeness: Only datasets from two developed countries in Europe and America are used, failing to cover load data from developing countries (such as China) or those with different power grid structures; data quality issues in actual engineering scenarios, such as data missing and sensor noise, are not considered. It is recommended to expand the dataset to emerging markets.

The dimension of robustness testing is single: existing robustness tests focus on hyperparameter selection and ablation experiments, and do not involve tests on model structure perturbations or changes in data distribution; they also do not verify the performance degradation of the model in ultra-long-term predictions (such as quarterly or annual predictions). It is recommended to supplement robustness tests involving model structure replacement and data distribution adjustment; add ultra-long-term prediction experiments, analyze the laws of performance degradation, and propose optimization schemes.

Non-standard use of academic terms: some terms lack clear definitions; some expressions are inconsistent. It is suggested to clarify the definitions and boundaries of core terms, unify the annotation format for the first occurrence of terms (such as full name + abbreviation), and improve the academic standardization of the paper.

Reviewer #4: (No Response)

**********

7. PLOS authors have the option to publish the peer review history of their article (what does this mean?). If published, this will include your full peer review and any attached files.

If you choose “no”, your identity will remain anonymous but your review may still be made public.

**Do you want your identity to be public for this peer review?**  For information about this choice, including consent withdrawal, please see our Privacy Policy.

Reviewer #2: No

Reviewer #3: No

Reviewer #4: No

**********

[NOTE: If reviewer comments were submitted as an attachment file, they will be attached to this email and accessible via the submission site. Please log into your account, locate the manuscript record, and check for the action link "View Attachments". If this link does not appear, there are no attachment files.]

To ensure your figures meet our technical requirements, please review our figure guidelines: https://journals.plos.org/plosone/s/figures

You may also use PLOS’s free figure tool, NAAS, to help you prepare publication quality figures: https://journals.plos.org/plosone/s/figures#loc-tools-for-figure-preparation.

NAAS will assess whether your figures meet our technical requirements by comparing each figure against our figure specifications.

# Author comment: Author response to Decision Letter 2

**DOI:** 10.1371/journal.pone.0351428.r004

4 Dec 2025

The manuscript has been revised according to comments.

Attachment Submitted filename: Response to Reviewers.docx

# Peer review: Decision Letter 2

**DOI:** 10.1371/journal.pone.0351428.r005

**Authors:** Zeyar Aung, Zeyar Aung, Zeyar Aung

25 Mar 2026

PONE-D-25-31195R2

Power Load Forecasting Combining Deep Learning Models and Improved CLPO Algorithm

PLOS One

Dear Dr. Wang,

Thank you for submitting your manuscript to PLOS ONE. After careful consideration, we feel that it has merit but does not fully meet PLOS ONE’s publication criteria as it currently stands. Therefore, we invite you to submit a revised version of the manuscript that addresses the points raised during the review process.

**Major revision:**

The paper still needs improvements.

Please seriously address the comments by the reviewers. (One reviewer complained that he/her comments were not fully addressed.)

Please submit your revised manuscript by May 09 2026 11:59PM. If you will need more time than this to complete your revisions, please reply to this message or contact the journal office at plosone@plos.org. When you're ready to submit your revision, log on to https://www.editorialmanager.com/pone/ and select the 'Submissions Needing Revision' folder to locate your manuscript file.

Please include the following items when submitting your revised manuscript:

- A letter that responds to each point raised by the academic editor and reviewer(s). You should upload this letter as a separate file labeled 'Response to Reviewers'.
- A marked-up copy of your manuscript that highlights changes made to the original version. You should upload this as a separate file labeled 'Revised Manuscript with Track Changes'.
- An unmarked version of your revised paper without tracked changes. You should upload this as a separate file labeled 'Manuscript'.

If you would like to make changes to your financial disclosure, please include your updated statement in your cover letter. Guidelines for resubmitting your figure files are available below the reviewer comments at the end of this letter.

If applicable, we recommend that you deposit your laboratory protocols in protocols.io to enhance the reproducibility of your results. Protocols.io assigns your protocol its own identifier (DOI) so that it can be cited independently in the future. For instructions see: https://journals.plos.org/plosone/s/submission-guidelines#loc-laboratory-protocols. Additionally, PLOS ONE offers an option for publishing peer-reviewed Lab Protocol articles, which describe protocols hosted on protocols.io. Read more information on sharing protocols at https://plos.org/protocols?utm_medium=editorial-email&utm_source=authorletters&utm_campaign=protocols.

As the corresponding author, your ORCID iD is verified in the submission system and will appear in the published article. PLOS supports the use of ORCID, and we encourage all coauthors to register for an ORCID iD and use it as well. Please encourage your coauthors to verify their ORCID iD within the submission system before final acceptance, as unverified ORCID iDs will not appear in the published article. *Only*  the individual author can complete the verification step; PLOS staff *cannot*  verify ORCID iDs on behalf of authors.

We look forward to receiving your revised manuscript.

Kind regards,

Zeyar Aung

Academic Editor

PLOS One

Journal Requirements:

If the reviewer comments include a recommendation to cite specific previously published works, please review and evaluate these publications to determine whether they are relevant and should be cited. There is no requirement to cite these works unless the editor has indicated otherwise.

[Note: HTML markup is below. Please do not edit.]

Reviewers' comments:

Reviewer's Responses to Questions

**Comments to the Author**

1. If the authors have adequately addressed your comments raised in a previous round of review and you feel that this manuscript is now acceptable for publication, you may indicate that here to bypass the “Comments to the Author” section, enter your conflict of interest statement in the “Confidential to Editor” section, and submit your "Accept" recommendation.

Reviewer #2: (No Response)

Reviewer #5: (No Response)

**********

2. Is the manuscript technically sound, and do the data support the conclusions?

The manuscript must describe a technically sound piece of scientific research with data that supports the conclusions. Experiments must have been conducted rigorously, with appropriate controls, replication, and sample sizes. The conclusions must be drawn appropriately based on the data presented.

Reviewer #2: Yes

Reviewer #5: Yes

**********

3. Has the statistical analysis been performed appropriately and rigorously?

Reviewer #2: N/A

Reviewer #5: Yes

**********

4. Have the authors made all data underlying the findings in their manuscript fully available?

The PLOS Data policy requires authors to make all data underlying the findings described in their manuscript fully available without restriction, with rare exception (please refer to the Data Availability Statement in the manuscript PDF file). The data should be provided as part of the manuscript or its supporting information, or deposited to a public repository. For example, in addition to summary statistics, the data points behind means, medians and variance measures should be available. If there are restrictions on publicly sharing data—e.g. participant privacy or use of data from a third party—those must be specified.

Reviewer #2: Yes

Reviewer #5: Yes

**********

5. Is the manuscript presented in an intelligible fashion and written in standard English?

PLOS ONE does not copyedit accepted manuscripts, so the language in submitted articles must be clear, correct, and unambiguous. Any typographical or grammatical errors should be corrected at revision, so please note any specific errors here.

Reviewer #2: Yes

Reviewer #5: Yes

**********

6. Review Comments to the Author

Please use the space provided to explain your answers to the questions above. You may also include additional comments for the author, including concerns about dual publication, research ethics, or publication ethics. (Please upload your review as an attachment if it exceeds 20,000 characters)

Reviewer #2: Authors should seriously incorporate the comments provided in the previous round. I am not satisfied after reading R2

Reviewer #5: Overall, the manuscript demonstrates technical depth, methodological integration, and practical relevance. The proposed model improved predictive performance compared to several baseline methods, supported by experimental validation on public datasets.

However, several aspects require clarification and strengthening to fully meet PLOS ONE publication criteria, methodological transparency, and clarity of presentation.

Major Strengths

1. The study proposes a well-structured hybrid framework combining:

• Multi-scale deep learning (TCN, LSTM, BiLSTM)

• Adaptive feature fusion via WR

• Meta-heuristic optimization (CLPO)

• Error correction through ECM

This design is conceptually sound and addresses known limitations in single-model forecasting approaches.

2. The manuscript includes:

• Ablation studies

• Benchmark comparisons with recent models

• Statistical validation (e.g., t-test with p < 0.05)

• Testing under extreme weather and varying load conditions

These elements enhance the credibility of the reported performance improvements

3. The use of UK-NGED and EDF-SGD datasets improves transparency and allows for potential reproducibility and benchmark against existing studies

4. The application to smart grid scheduling, renewable energy integration, and extreme scenarios demonstrates strong real-world applicability.

5• Scientific validity: Generally sound, but requires clearer methodological transparency

6• Data availability: Stated as available, but should be more explicitly verifiable.

— — —

Recommendation

1-

• Lack of implementation details (e.g., architecture depth, activation functions, batch size, training epochs)

• Need to mention to the code availability or repository link

• description of preprocessing steps for data insufficient

Recommendation: support full experimental reproducibility details, including code or pseudo-code expansion, parameter settings, and preprocessing pipeline.

2- The Methods section is very dense, with:

• Excessive mathematical formulations without explanation

• Some overly long paragraphs

• Limited guidance for researchers to follow the study

Recommendation:

• boost structure using subheadings and explanations

• Provide a simplified summary for pipeline

• How interacts operationally for each component done (WR, CLPO, ECM)

3. While the integration is extensive, the incremental novelty of each component is not sufficiently emphasized.

Recommendation:

• Distinguish what is new versus adapted in details

• support the study with stronger comparative discussion with closely related hybrid models

4. Although t-tests are declear, the study lacks:

• Confidence intervals

• Variance analysis with runs

Recommendation:

Include additional metrics and robustness checks to support the validity of conclusions.

5. A thorough language revision is required to be more publication standards.

6.

• Clarify all abbreviations, only at the first of use (e.g., CDL, ECM, WR).

• Improve captions for figures, and the figures 3–7 require clearer explanation.

• Some claims (e.g., “high practical value”) should be supported with more concrete evidence.

Data availability: Stated as available, but should be more explicitly verifiable.

7.

- Scientific validity: Generally sound, but requires clearer methodological transparency

- Data availability: Stated as available, but should be more explicitly verifiable.

**********

7. PLOS authors have the option to publish the peer review history of their article (what does this mean?). If published, this will include your full peer review and any attached files.

If you choose “no”, your identity will remain anonymous but your review may still be made public.

**Do you want your identity to be public for this peer review?**  For information about this choice, including consent withdrawal, please see our Privacy Policy.

Reviewer #2: No

Reviewer #5: No

**********

[NOTE: If reviewer comments were submitted as an attachment file, they will be attached to this email and accessible via the submission site. Please log into your account, locate the manuscript record, and check for the action link "View Attachments". If this link does not appear, there are no attachment files.]

To ensure your figures meet our technical requirements, please review our figure guidelines: https://journals.plos.org/plosone/s/figures

You may also use PLOS’s free figure tool, NAAS, to help you prepare publication quality figures: https://journals.plos.org/plosone/s/figures#loc-tools-for-figure-preparation.

NAAS will assess whether your figures meet our technical requirements by comparing each figure against our figure specifications.

# Author comment: Author response to Decision Letter 3

**DOI:** 10.1371/journal.pone.0351428.r006

15 Apr 2026

The manuscript has been revised according to comments.

Attachment Submitted filename: Response_to_Reviewers_auresp_3.docx

# Peer review: Decision Letter 3

**DOI:** 10.1371/journal.pone.0351428.r007

**Authors:** Zeyar Aung, Zeyar Aung, Zeyar Aung, Zeyar Aung

27 May 2026

Power Load Forecasting Combining Deep Learning Models and Improved CLPO Algorithm

PONE-D-25-31195R3

Dear Dr. Wang,

We’re pleased to inform you that your manuscript has been judged scientifically suitable for publication and will be formally accepted for publication once it meets all outstanding technical requirements.

Within one week, you’ll receive an e-mail detailing the required amendments. When these have been addressed, you’ll receive a formal acceptance letter and your manuscript will be scheduled for publication.

An invoice will be generated when your article is formally accepted. Please note, if your institution has a publishing partnership with PLOS and your article meets the relevant criteria, all or part of your publication costs will be covered. Please make sure your user information is up-to-date by logging into Editorial Manager at Editorial Manager® and clicking the ‘Update My Information' link at the top of the page. For questions related to billing, please contact billing support.

If your institution or institutions have a press office, please notify them about your upcoming paper to help maximize its impact. If they’ll be preparing press materials, please inform our press team as soon as possible -- no later than 48 hours after receiving the formal acceptance. Your manuscript will remain under strict press embargo until 2 pm Eastern Time on the date of publication. For more information, please contact onepress@plos.org.

Kind regards,

Zeyar Aung

Academic Editor

PLOS One

Additional Editor Comments (optional):

Reviewers' comments:

Reviewer's Responses to Questions

**Comments to the Author**

1. If the authors have adequately addressed your comments raised in a previous round of review and you feel that this manuscript is now acceptable for publication, you may indicate that here to bypass the “Comments to the Author” section, enter your conflict of interest statement in the “Confidential to Editor” section, and submit your "Accept" recommendation.

Reviewer #2: All comments have been addressed

Reviewer #5: All comments have been addressed

**********

2. Is the manuscript technically sound, and do the data support the conclusions?

The manuscript must describe a technically sound piece of scientific research with data that supports the conclusions. Experiments must have been conducted rigorously, with appropriate controls, replication, and sample sizes. The conclusions must be drawn appropriately based on the data presented.

Reviewer #2: Yes

Reviewer #5: Yes

**********

3. Has the statistical analysis been performed appropriately and rigorously?

Reviewer #2: N/A

Reviewer #5: Yes

**********

4. Have the authors made all data underlying the findings in their manuscript fully available?

The PLOS Data policy requires authors to make all data underlying the findings described in their manuscript fully available without restriction, with rare exception (please refer to the Data Availability Statement in the manuscript PDF file). The data should be provided as part of the manuscript or its supporting information, or deposited to a public repository. For example, in addition to summary statistics, the data points behind means, medians and variance measures should be available. If there are restrictions on publicly sharing data—e.g. participant privacy or use of data from a third party—those must be specified.

Reviewer #2: Yes

Reviewer #5: Yes

**********

5. Is the manuscript presented in an intelligible fashion and written in standard English?

PLOS ONE does not copyedit accepted manuscripts, so the language in submitted articles must be clear, correct, and unambiguous. Any typographical or grammatical errors should be corrected at revision, so please note any specific errors here.

Reviewer #2: Yes

Reviewer #5: Yes

**********

6. Review Comments to the Author

Please use the space provided to explain your answers to the questions above. You may also include additional comments for the author, including concerns about dual publication, research ethics, or publication ethics. (Please upload your review as an attachment if it exceeds 20,000 characters)

Reviewer #2: All of my raised points and concerns are now properly addressed. Well done authors and I will cite these findings in my future papers

Reviewer #5: (No Response)

**********

7. PLOS authors have the option to publish the peer review history of their article (what does this mean?). If published, this will include your full peer review and any attached files.

If you choose “no”, your identity will remain anonymous but your review may still be made public.

**Do you want your identity to be public for this peer review?**  For information about this choice, including consent withdrawal, please see our Privacy Policy.

Reviewer #2: No

Reviewer #5: No

**********

# Editor report: Acceptance letter

**DOI:** 10.1371/journal.pone.0351428.r008

**Authors:** Zeyar Aung, Zeyar Aung, Zeyar Aung, Zeyar Aung

PONE-D-25-31195R3

PLOS One

Dear Dr. Wang,

I'm pleased to inform you that your manuscript has been deemed suitable for publication in PLOS One. Congratulations! Your manuscript is now being handed over to our production team.

At this stage, our production department will prepare your paper for publication. This includes ensuring the following:

* All references, tables, and figures are properly cited

* All relevant supporting information is included in the manuscript submission,

* There are no issues that prevent the paper from being properly typeset

You will receive further instructions from the production team, including instructions on how to review your proof when it is ready. Please keep in mind that we are working through a large volume of accepted articles, so please give us a few days to review your paper and let you know the next and final steps.

Lastly, if your institution or institutions have a press office, please let them know about your upcoming paper now to help maximize its impact. If they'll be preparing press materials, please inform our press team within the next 48 hours. Your manuscript will remain under strict press embargo until 2 pm Eastern Time on the date of publication. For more information, please contact onepress@plos.org.

You will receive an invoice from PLOS for your publication fee after your manuscript has reached the completed accept phase. If you receive an email requesting payment before acceptance or for any other service, this may be a phishing scheme. Learn how to identify phishing emails and protect your accounts at https://explore.plos.org/phishing.

If we can help with anything else, please email us at customercare@plos.org.

Thank you for submitting your work to PLOS ONE and supporting open access.

Kind regards,

PLOS ONE Editorial Office Staff

on behalf of

Dr. Zeyar Aung

Academic Editor

PLOS One
